# A General Framework for Tilings, Delone Sets, Functions, and Measures and Their Interrelation

**DOI:** 10.1007/s00454-019-00081-2

**Published:** 2019-07-17

**Authors:** Yasushi Nagai

**Affiliations:** Montanuniversität, Department Mathematik und Informationstechnologie, Lehrstuhl für Mathematik und Statistik, Franz Josef Strasse 18, 8700 Leoben, Austria

**Keywords:** Tiling, Delone set, Almost periodic function, Almost periodic measure

## Abstract

We define a general framework that includes objects such as tilings, Delone sets, functions, and measures. We define local derivability and mutual local derivability (MLD) between any two of these objects in order to describe their interrelation. This is a generalization of the local derivability and MLD (or S-MLD) for tilings and Delone sets which are used in literature, under a mild assumption. We show that several canonical maps in aperiodic order send an object $\mathcal {P}$ to one that is MLD with $\mathcal {P}$. Moreover, we show that, for an object $\mathcal {P}$ and a class $\Sigma$ of objects, a mild condition on them ensures that there exists some $\mathcal {Q}\in \Sigma$ that is MLD with $\mathcal {P}$. As an application, we study pattern-equivariant functions. In particular, we show that the space of all pattern-equivariant functions contains all the information on the original object up to MLD, in a quite general setting.

## Introduction

Objects such as tilings, Delone (multi)sets, measures, and almost periodic functions have been investigated in literature, especially after the discovery of quasicrystals in materials science. Quasicrystals are not periodic but have long-range order, and the above mathematical objects with similar properties are studied intensively. In particular, nonperiodic objects with pure point diffraction measures are interesting. One fundamental problem is which objects have pure point diffraction measures, with a classification of such objects being an ultimate goal.

To study the cohomology of the above-mentioned objects, Kellendonk [7] defined pattern-equivariant functions. There are strongly and weakly pattern-equivariant functions. It is easy to see that the $C^*$-algebra of all weakly pattern-equivariant functions of a finite local complexity (FLC) object $\mathcal {P}$ remembers the original $\mathcal {P}$ up to homeomorphism of the corresponding topological spaces. This is an example of a phenomenon that is often observed in mathematics, viz. that the space of functions that reflect the structure of a mathematical object remembers the original object (for example, consider a locally compact Abelian group and its Pontryagin dual, or a smooth manifold and its space of smooth functions).

Given this, it is natural to ask what the space of strongly pattern-equivariant functions remembers. We use the concepts of “locally derivable” and “mutually locally derivable” (MLD), which were defined in [4]. Recall that, if $\mathcal {P}$ is an object such as a tiling or Delone set, a function *f* is $\mathcal {P}$-equivariant if its value at $x\in {\mathbb {R}}^d$ is determined by the local behavior of $\mathcal {P}$ near *x*, for each *x*. Intuitively, this means that *f* is “locally derivable” from $\mathcal {P}$; that is, $\mathcal {P}\overset{{\mathrm {LD}}}{\rightarrow }f$. Moreover, often there is a function $f_{\mathcal {P}}$ that is “MLD” with $\mathcal {P}$, namely $\mathcal {P}\overset{{\mathrm {MLD}}}{\leftrightarrow }f_{\mathcal {P}}$; the local behavior of $\mathcal {P}$ near *x* is also determined by the local behavior of $f_{\mathcal {P}}$ near *x*, for each $x\in {\mathbb {R}}^d$. Assuming this, we see that, given two objects $\mathcal {P}$ and $\mathcal {Q}$, if the spaces $\Sigma _{\mathcal {P}}=\{f\,{|}\, \mathcal {P}\overset{{\mathrm {LD}}}{\rightarrow }f\}$ and $\Sigma _{\mathcal {Q}}=\{f\,{|}\,\mathcal {Q}\overset{{\mathrm {LD}}}{\rightarrow }f\}$ of their strongly pattern-equivariant functions are the same, then since $f_{\mathcal {Q}}\in \Sigma _{\mathcal {P}}$, we have $\mathcal {P}\overset{{\mathrm {LD}}}{\rightarrow }f_{\mathcal {Q}}\overset{{\mathrm {LD}}}{\rightarrow }\mathcal {Q}$, and so, by transitivity, $\mathcal {P}\overset{{\mathrm {LD}}}{\rightarrow }\mathcal {Q}$. The converse $\mathcal {Q}\overset{{\mathrm {LD}}}{\rightarrow }\mathcal {P}$ is proved in the same way, and we have $\mathcal {P}\overset{{\mathrm {MLD}}}{\leftrightarrow }\mathcal {Q}$. We have $\mathcal {P}\overset{{\mathrm {MLD}}}{\leftrightarrow }\mathcal {Q}$ if and only if $\Sigma _{\mathcal {P}}=\Sigma _{\mathcal {Q}}$.

Strongly pattern-equivariant functions were generalized by Rand [11] to include rotations or $\mathrm{O}(d)$-actions on objects. The same argument works as above, if we replace “MLD” with “S-MLD.”

However, local derivability and MLD are not defined for functions. The goal of this article is to define local derivability and MLD for more general classes of interest, including functions, and to justify the above argument. In particular, we prove the existence of $f_{\mathcal {P}}$ above (with or without $\mathrm{O}(d)$-action). Such an $f_{\mathcal {P}}$ may be constructed in an ad hoc way for each example, but we give a general sufficient condition for its existence.

To define usual local derivability and MLD, all we need to have is an operation of “cutting-off” and a group action of translation and, sometimes, $\mathrm{O}(d)$-action. We first axiomatize the properties that these operations should have, and a set with a cutting-off operation and group action with the axioms is called an “abstract pattern space.” The elements of abstract pattern spaces, such as tilings, Delone sets, and functions, are called “abstract patterns.” We give the axiom for the cutting-off operation in Sect. 2.1. Next, in Sect. 2.2, we give an axiom on the relation between the cutting-off operation and group action. The structure of abstract pattern spaces is enough to define local derivability and MLD, in a more general context. In Sect. 2.3, we define these concepts. (The structure is also enough to define the local matching uniform structures and topologies, and prove its completeness under a mild condition [10].)

We see that the new definition of local derivability and MLD is correct by showing that two objects that are often identified in aperiodic order are MLD, in the latter part of Sect. 2.3; for example, a Delone set and its Dirac comb, and a Delone set and its punctured Voronoi tiling are MLD. Although these are more or less folklore, this is the first attempt to define and prove MLD explicitly. Moreover, we prove MLD in a more general setting than is currently known.

It is easy to prove the transitivity of the relations $\overset{{\mathrm {LD}}}{\rightarrow }$ and $\overset{{\mathrm {MLD}}}{\leftrightarrow }$. It is also easy to prove the existence of $f_{\mathcal {P}}$ if we do not consider the $\mathrm{O}(d)$-actions; in this case, an object $\mathcal {P}$ is often MLD with a Delone multiset *D*, and $f_{\mathcal {P}}$ is obtained by putting a bump function on each point of *D*, the heights of which are dependent on the color of the point. We deal with this case in Sect. 3 and prove Theorem 3.6, which states that the space of pattern-equivariant functions remembers the original object up to MLD. As pointed out by a referee, if $\mathcal {P}$ is an FLC Delone set, this result is known, but we generalize it to drop the assumption of FLC and relative denseness, and allow the ambient space and its symmetry group to be more general. We use the structure of graphs, the vertexes of which are abstract patterns such as tilings and Delone sets, and the edges of which are $\overset{{\mathrm {LD}}}{\rightarrow }$, to prove this theorem; this method itself is new and interesting.

If we consider $\mathrm{O}(d)$-actions, the construction is more complicated, but we can give a sufficient condition for $f_{\mathcal {P}}$ to exist (Theorem 4.40). Section 4 is devoted to setting a terminology to state this theorem. Often there is an ad hoc way to construct such $f_{\mathcal {P}}$, but we give a general construction. Since the proof is long and technical, we postpone it to Appendix A. In Sect. 5, we prove that, under a mild condition, the space of strongly pattern-equivariant functions remembers the original object up to MLD, when we consider $\mathrm{O}(d)$-action.

Finally, let us discuss whether our argument is topological or metrical. Our argument is metrical and depends on the choice of metric. For example, in literature, a subset *D* of ${\mathbb {R}}^d$ is relatively dense if there is a compact subset *K* of ${\mathbb {R}}^d$ such that $D+K={\mathbb {R}}^d$. This definition makes sense if we replace ${\mathbb {R}}^d$ with a locally compact Abelian group. However, for general topological spaces, this does not make sense, since there is no group structure available. We have to assume that the space admits either a metric or a group action in order to define relative denseness. In this article, we assume the existence of metric for the spaces *X* where abstract patterns such as tilings live, and say a subset *D* of *X* is relatively dense if there is $R>0$ such that any balls in *X* of radius *R* contain points in *D*. By using metric structure, we can define other useful notions, such as “uniformly discrete,” which means that the distances between two points in a set $D\subset X$ are bounded from below, and for abstract patterns the notion of “consists of bounded components” (Definition 2.24), which means that the diameters of tiles are bounded from above if the abstract patterns are tilings. We use these metric-dependent notions throughout the article. In particular, we limit the relevance of this article to the case where abstract patterns consist of bounded components.

However, it is desirable to put a topological assumption on the metrics. As pointed out by a referee, if we consider a metric $\rho '(x,y)=\min \{1,\rho (x,y)\}$ of ${\mathbb {R}}^d$, where $\rho$ is the standard Euclidean metric, any nonempty subset *D* of ${\mathbb {R}}^d$ is relatively dense with respect to our definition, which contradicts the standard definition of relative denseness. Thus, *we always assume that the metrics we consider are proper*, a topological condition on the metrics. By this assumption, some definitions that use a metric become equivalent to a topological notion.

### Notation 1.1

For a metric space $(X,\rho )$, the *closed* ball with center $x\in X$ and radius $r>0$ is denoted by *B*(*x*, *r*); that is, $B(x,r)=\{y\in X\,{|}\, \rho (x,y)\leqq r\}$. As mentioned above, every metric we consider on topological spaces is assumed to be proper, which means that all closed balls are compact.^1^

For a positive integer *d*, let $\rho$ be the Euclidean metric for the Euclidean space ${\mathbb {R}}^d$. Let $\mathrm{E}(d)$ be the group of all isometries on the Euclidean space ${\mathbb {R}}^d$ and $\mathrm{O}(d)$ be the orthogonal group. There is a group isomorphism ${\mathbb {R}}^d\rtimes \mathrm{O}(d)\rightarrow \mathrm{E}(d)$, by which we can identify these two groups. Thus, elements of $\mathrm{E}(d)$ are recognized as pairs (*a*, *A*) of $a\in {\mathbb {R}}^d$ and $A\in \mathrm{O}(d)$. For $\mathrm{E}(d)$, define a metric $\rho _{\mathrm{E}(d)}$ by $\rho _{\mathrm{E}(d)}((a,A),(b,B))=\rho (a,b)+\Vert A-B\Vert$, where $\Vert \cdot \Vert$ is the operator norm for the operators on the Banach space ${\mathbb {R}}^d$ with the Euclidean norm. For any closed subgroup $\Gamma$ of $\mathrm{E}(d)$, the restriction $\rho _{\Gamma }$ of $\rho _{\mathrm{E}(d)}$ is a left-invariant metric for $\Gamma$. Moreover, for any $\gamma ,\eta \in \Gamma$, we have

1 $$\begin{aligned} \rho (\gamma 0,\eta 0)\leqq \rho _{\Gamma }(\gamma ,\eta )\leqq \rho (\gamma 0,\eta 0)+2. \end{aligned}$$

We set $$\mathbb {T}=\{z\in \mathbb {C}\,{|}\, |z|=1\}$$.

For any group $\Gamma$ which acts on a set *X*, its isotropy group for a point $x\in X$ is denoted by $\Gamma _x$; That is, $\Gamma _x=\{\gamma \in \Gamma \,{|}\,\gamma x=x\}$. The identity element of any group is denoted by *e*. If $\mathcal {P}$ is an object such as a patch, a function, a measure, or a subset of *X*, its group of symmetry is by definition ${{\,\mathrm{Sym}\,}}_{\Gamma }\mathcal {P}=\{\gamma \in \Gamma \,{|}\,\gamma \mathcal {P}=\mathcal {P}\}$ (a special case of isotropy groups).

## General Theory of Abstract Pattern Spaces

*In this sectionXrepresents a nonempty topological space unless otherwise stated.* First, in Sect. 2.1, we define “abstract pattern space.” Several spaces such as the space of patches and the space of subsets of ${\mathbb {R}}^d$ have an operation of “cutting-off”; for example, for a discrete set $D\subset {\mathbb {R}}^d$ and a subset *C* of ${\mathbb {R}}^d$, we can “cut off” *D* by the window *C* by taking the intersection $D\cap C$. We axiomatize the properties that such a cutting-off operation should have and obtain the notion of abstract pattern spaces. We will see that several spaces of objects such as patches, subsets of ${\mathbb {R}}^d$, functions, and measures are captured in this framework.

In Sect. 2.2, we incorporate the group action into the theory of abstract pattern spaces. These structures are shown to be enough to define local derivability and MLD in Sect. 2.3, where we also show that several pairs of two objects that are frequently identified in aperiodic order are MLD.

### Definition and Examples of Abstract Pattern Spaces

Here, we define the framework of “abstract pattern spaces” for objects such as tilings and Delone sets.

#### Notation 2.1

The set of all closed subsets of *X* is denoted by ${{\,\mathrm{Cl}\,}}(X)$.

#### Definition 2.2

A nonempty set $\Pi$ equipped with a map

2 $$\begin{aligned} \Pi \times {{\,\mathrm{Cl}\,}}(X)\ni (\mathcal {P},C)\mapsto \mathcal {P}\wedge C\in \Pi \end{aligned}$$

such that

1. $(\mathcal {P}\wedge C_1)\wedge C_2=\mathcal {P}\wedge (C_1\cap C_2)$ for any $\mathcal {P}\in \Pi$ and any $C_1,C_2\in {{\,\mathrm{Cl}\,}}(X)$, and
2. for any $\mathcal {P}\in \Pi$ there exists $C_{\mathcal {P}}\in {{\,\mathrm{Cl}\,}}(X)$ such that
  $$\begin{aligned} \mathcal {P}\wedge C=\mathcal {P}\iff C\supset C_{\mathcal {P}}, \end{aligned}$$
  for any $$C\in {{\,\mathrm{Cl}\,}}(X)$$,

is called an *abstract pattern space overX*. The map (2) is called the *cutting-off operation* of the abstract pattern space $\Pi$. The closed set $C_{\mathcal {P}}$ that appears in 2. is unique. It is called the *support* of $\mathcal {P}$ and is denoted by ${{\,\mathrm{supp}\,}}\mathcal {P}$. Elements in $\Pi$ are called *abstract patterns* in $\Pi$.

#### Remark 2.3

Note that the symbol $\cap$ in the first axiom for abstract pattern spaces is the intersection of two sets. Note also that, if $A\supset B$, *A* and *B* may be equal.

#### Remark 2.4

It is sometimes impossible to recover ${{\,\mathrm{supp}\,}}\mathcal {P}$ from the information on ${{\,\mathrm{supp}\,}}(\mathcal {P}\wedge K)$, where *K* runs through the set of all compact subsets of *X*. Consider a noncompact *X* and an abstract pattern space ${{\,\mathrm{Pattern}\,}}(X)$. The definition of this abstract pattern space is given in Definition 2.11, but for now all we need is that a one-point set $\{X\}$ is an abstract pattern in this abstract pattern space, the cutting-off operation is given by

$$\begin{aligned} \{X\}\wedge C={\left\{ \begin{array}{ll} \{X\} &{}\text { if C=X},\\ \emptyset &{}\text { otherwise}, \end{array}\right. } \end{aligned}$$

and ${{\,\mathrm{supp}\,}}\{X\}=X$. For compact $K\subset X$, $\{X\}\wedge K=\emptyset$, so the support is not recovered from the information on supports of abstract patterns given by cutting-off by compact sets.

This phenomenon occurs because the “tile” *X* is too big. If the sizes of  “components” are bounded from above, we can recover the support. We can show that, if $\mathcal {P}$ consists of bounded components (Definition 2.24), we can recover the support ${{\,\mathrm{supp}\,}}\mathcal {P}$.

We now prove a simple lemma on the relation between the support and the cutting-off operation.

#### Lemma 2.5

Let $\Pi$ be an abstract pattern space over *X*. For any $\mathcal {P}\in \Pi$ and $C\in {{\,\mathrm{Cl}\,}}(X)$, we have ${{\,\mathrm{supp}\,}}(\mathcal {P}\wedge C)\subset ({{\,\mathrm{supp}\,}}\mathcal {P})\cap C$.

#### Proof

$$\begin{aligned} \quad \quad \quad \quad \quad \quad (\mathcal {P}\wedge C)\wedge (({{\,\mathrm{supp}\,}}\mathcal {P})\cap C)=(\mathcal {P}\wedge {{\,\mathrm{supp}\,}}\mathcal {P})\wedge C=\mathcal {P}\wedge C. \quad \quad \quad \quad \quad \quad \square \end{aligned}$$

#### Remark 2.6

The inclusion in Lemma 2.5 may be strict. In fact, in the abstract pattern space ${{\,\mathrm{Patch}\,}}(X)$ of all patches (Example 2.9), if compared with the tiles in a patch $\mathcal {P}$, a closed set *C* is too small, then $\mathcal {P}\wedge C=\emptyset$ and so ${{\,\mathrm{supp}\,}}(\mathcal {P}\wedge C)=\emptyset$. On the other hand, ${{\,\mathrm{supp}\,}}\mathcal {P}$ and *C* may have nonempty intersection even if *C* is small. For example, consider the tiling $\mathcal {P}=\{(0,1)^d+x\,{|}\, x\in \mathbb {Z}^d\}$ and $C=B(0,1/2)$; then $\mathcal {P}\wedge C=\emptyset$ and $({{\,\mathrm{supp}\,}}\mathcal {P})\cap C=C$.

We now list several examples of abstract pattern spaces. We will see that objects in aperiodic order are in fact abstract patterns. Of course, the most interesting case is that where $X={\mathbb {R}}^d$, but we deal with general *X*, since this does not change the description.

#### Example 2.7

(*The space of labeled patches* [8, 9]) Let *L* be a set. An *L*-labeled tile is a pair (*T*, *l*) of a compact subset *T* of *X* and $l\in L$ such that $T=\overline{T^{\circ }}$ (the closure of the interior). An *L*-labeled patch is a collection $\mathcal {P}$ of *L*-labeled tiles such that, if $(T,l), (S,k)\in \mathcal {P}$, then either $T^{\circ }\cap S^{\circ }=\emptyset$, or $S=T$ and $l=k$. For an *L*-labeled patch $\mathcal {P}$, define the support of $\mathcal {P}$ via

$$\begin{aligned} {{\,\mathrm{supp}\,}}\mathcal {P}=\overline{\bigcup _{(T,l)\in \mathcal {P}}T}. \end{aligned}$$

An *L*-labeled patch $\mathcal {T}$ with ${{\,\mathrm{supp}\,}}\mathcal {T}=X$ is called an *L*-labeled tiling. Sometimes we suppress *L* and call such tilings labeled tilings.

For an *L*-labeled patch $\mathcal {P}$ and $C\in {{\,\mathrm{Cl}\,}}(X)$, define a cutting-off operation via

$$\begin{aligned} \mathcal {P}\wedge C=\big \{(T,l)\in \mathcal {P}\,{|}\, T\subset C\big \}. \end{aligned}$$

The space ${{\,\mathrm{Patch}\,}}_L(X)$ of all *L*-labeled patches is an abstract pattern space over *X* with this cutting-off operation.

#### Remark 2.8

There is another operation of “cutting off” *L*-labeled patches, which is defined via

$$\begin{aligned} \mathcal {P}\sqcap C=\big \{(T,l)\in \mathcal {P}\,{|}\, T\cap C\ne \emptyset \big \}. \end{aligned}$$

However, it does not define a pattern space structure, since it satisfies neither of the two conditions in the definition. Since we often assume that the diameters of tiles in a tiling are bounded from above, in most cases the two operations $\wedge$ and $\sqcap$ are essentially the same.

Next, we deal with patches and tilings such that the definition of tile is slightly different. The advantage of this new definition will be explained immediately after.

#### Example 2.9

(*The space of patches in a metric space*) Let *X* be a (proper) metric space. An open, nonempty, and bounded subset of *X* is called a tile (in *X*). A set $\mathcal {P}$ of tiles such that, if $S,T\in \mathcal {P}$, then either $S=T$ or $S\cap T=\emptyset$ is called a patch (in *X*). The set of all patches in *X* is denoted by ${{\,\mathrm{Patch}\,}}(X)$. For $\mathcal {P}\in {{\,\mathrm{Patch}\,}}(X)$ and $C\in {{\,\mathrm{Cl}\,}}(X)$, set

3 $$\begin{aligned} \mathcal {P}\wedge C=\big \{T\in \mathcal {P}\,{|}\, T\subset C\big \}. \end{aligned}$$

With this cutting-off operation, ${{\,\mathrm{Patch}\,}}(X)$ becomes an abstract pattern space over *X*. For $\mathcal {P}\in {{\,\mathrm{Patch}\,}}(X)$, its support is

$$\begin{aligned} {{\,\mathrm{supp}\,}}\mathcal {P}=\overline{\bigcup _{T\in \mathcal {P}}T}. \end{aligned}$$

Patches $\mathcal {P}$ with ${{\,\mathrm{supp}\,}}\mathcal {P}=X$ are called tilings.

#### Remark 2.10

This definition of tiles differs from those in literature. Usually, tiles are defined to be (1) a compact set that is the closure of its interior (Example 2.7, compare [5]), or in the Euclidean case, (2) a polygonal subset of ${\mathbb {R}}^d$ [14] or (3) a homeomorphic image of the closed unit ball (for example, see [1]). The advantage of our definition is that we can give punctures to tiles and we do not need to consider labels, thus avoiding slight abuses of language such as “tiles *T* and *S* have disjoint interiors” and simplify the notation. For example, we can define Robinson triangles [6, p. 537] as the following four tiles: (1) the interior of a triangle with side-lengths $\tau , \tau , 1$ (where $\tau =\frac{1+\sqrt{5}}{2}$) with one point on the left-hand side removed, (2) the similar open set but with one point on the right-hand side removed, (3) the interior of a triangle with side-lengths $1,1,\tau$ with one point on the right-hand side removed, and (4) the similar open set but with one point on the left-hand side removed. Giving punctures is also useful when we construct Voronoi tilings in Sect. 2.3, since in this case giving punctures is simpler than giving labels.

The usual labeled tilings (Example 2.7) are often MLD with tilings with open tiles (Example 2.9), so in this article we mainly deal with tilings with open tiles.

In literature, local derivability and MLD are defined for patterns, so we recall the definition, in order to see that our definition of local derivability and MLD is a generalization of the conventional definition.

#### Example 2.11

(*The space of patterns*) A set of nonempty subsets of *X* is called a pattern [2, p. 127].^2^ The set of all patterns in *X* is denoted by ${{\,\mathrm{Pattern}\,}}(X)$. ${{\,\mathrm{Pattern}\,}}(X)$ is an abstract pattern space over *X* by the cutting-off operation defined via (3).

There is another operation of “cutting-off” pattern $\mathcal {P}$, as follows:

#### Notation 2.12

For a pattern $\mathcal {P}$ in ${\mathbb {R}}^d$ and $C\subset {\mathbb {R}}^d$, we set

$$\begin{aligned} \mathcal {P}\sqcap C=\big \{T\in \mathcal {P}\,{|}\, T\cap C\ne \emptyset \big \}. \end{aligned}$$

However, this operation does not define a pattern space, since neither of the two conditions in the definition are satisfied.

Next we deal with point sets.

#### Example 2.13

(*The space of all locally finite subsets of a metric space*) Let *X* be a metric space. Let ${{\,\mathrm{LF}\,}}(X)$ be the set of all locally finite subsets of *X*; that is,

$$\begin{aligned} {{\,\mathrm{LF}\,}}(X)=\bigl \{D\subset X\mid \text {for all }x\in X\text { and }r>0, D\cap B(x,r)\text { is finite}\bigr \}. \end{aligned}$$

With the usual intersection ${{\,\mathrm{LF}\,}}(X)\times {{\,\mathrm{Cl}\,}}(X)\ni (D,C)\mapsto D\cap C\in {{\,\mathrm{LF}\,}}(X)$ of two subsets of *X* as a cutting-off operation, ${{\,\mathrm{LF}\,}}(X)$ is an abstract pattern space over *X*. For any $D\in {{\,\mathrm{LF}\,}}(X)$, its support is *D* itself.

#### Example 2.14

(*The space of all uniformly discrete subsets*) We say, for $r>0$, that a subset *D* of a metric space $(X,\rho )$ is *r*-uniformly discrete if $\rho (x,y)>r$ for any $x,y\in D$ with $x\ne y$. The set ${{\,\mathrm{UD}\,}}_r(X)$ of all *r*-uniformly discrete subsets of *X* is an abstract pattern space over *X* by the usual intersection as a cutting-off operation. If *D* is *r*-uniformly discrete for some $r>0$, we say that *D* is uniformly discrete. The set ${{\,\mathrm{UD}\,}}(X)=\bigcup _{r>0}{{\,\mathrm{UD}\,}}_r(X)$ of all uniformly discrete subsets of *X* is also an abstract pattern space over *X*.

Subsets *D* of *X* that are uniformly discrete and relatively dense in *X* are called *Delone sets*. “Relatively dense” is defined as follows: For $R>0$, a subset $D\subset X$ is *R*-relatively dense if $D\cap B(x,R)^{\circ }\ne \emptyset$ for each $x\in X$. A subset *D* of *X* is relatively dense if it is *R*-relatively dense for some *R*. For $X={\mathbb {R}}^d$ with the standard Euclidean metric, this definition is equivalent to the usual one [2, p. 12].

It is easy to see that any subset of *X* admits a cutting-off operation, as follows. Since this abstract pattern space includes ${{\,\mathrm{LF}\,}}(X)$ and ${{\,\mathrm{UD}\,}}(X)$, it is useful to introduce it.

#### Example 2.15

With the usual intersection of two subsets of *X* as a cutting-off operation, the set $2^X$ of all subsets of *X* and ${{\,\mathrm{Cl}\,}}(X)$ are abstract pattern spaces over *X*. For $A\in 2^X$, the support ${{\,\mathrm{supp}\,}}A$ is the closure of *A*.

For example, the union of all Ammann bars [6] in a Penrose tiling is an abstract pattern.

The following example plays an important role when we discuss pattern-equivariant functions in Sect. 3, because via this abstract pattern space structure on the set of functions, we can see that pattern-equivariant functions are functions that are locally derivable from the original abstract pattern. We later assume that *Y* has a topology and consider the space of all continuous maps from *X* to *Y*, but we need to consider the space of all maps as follows, since after cutting-off, continuous maps may become discontinuous.

The prototype of the following definition is the set of all mappings from ${\mathbb {R}}^d$ to $\mathbb {C}$. Given such a map *f* and a subset $C\subset {\mathbb {R}}^d$, we set the multiplication $f1_C$, where $1_C$ is the characteristic function for *C*, as the cutting-off $f\wedge C$. If we replace $\mathbb {C}$ with another set, we do not have such a multiplication operation, so we define as follows:

#### Example 2.16

(*The space of maps*) Let *Y* be a nonempty set. Take one element $y_0\in Y$ and fix it. The abstract pattern space ${{\,\mathrm{Map}\,}}(X,Y,y_0)$ is defined as follows: as a set the space is equal to ${{\,\mathrm{Map}\,}}(X,Y)$ of all mappings from *X* to *Y*; for $f\in {{\,\mathrm{Map}\,}}(X,Y,y_0)$ and $C\in {{\,\mathrm{Cl}\,}}(X)$, the cutting-off operation is defined by

$$\begin{aligned} (f\wedge C)(x)= {\left\{ \begin{array}{ll} f(x)&{}\text {if }x\in C,\\ y_0&{}\text {if }x\notin C. \end{array}\right. } \end{aligned}$$

With this operation, ${{\,\mathrm{Map}\,}}(X,Y,y_0)$ is an abstract pattern space over *X* and for $f\in {{\,\mathrm{Map}\,}}(X,Y,y_0)$ its support is ${{\,\mathrm{supp}\,}}f=\overline{\{x\in X\,{|}\, f(x)\ne y_0\}}$.

It is also desirable to be able to deal with measures such as the sum $\sum _{x\in D}\delta _x$ of Dirac measures and $f\,d\mu$, where *f* is a uniformly continuous bounded function on a locally compact Abelian group and $\mu$ is a Haar measure. We want to define a cutting-off operation such that $\left( \sum _{x\in D}\delta _x\right) \wedge C=\sum _{x\in D\cap C}\delta _x$. We do that in the following way.

Although these measures are interesting, they are often non-FLC, and our interest is mainly in FLC abstract patterns. (MLD is often useless for non-FLC objects.) Thus, the following example may be skipped on a first reading.

#### Example 2.17

(*The space of measures*) Let *X* be a locally compact $\sigma$-compact metric space. Let $C_c(X)$ be the space of all continuous and complex-valued functions on *X* which have compact supports.

Its dual space $C_c(X)^*$ with respect to the inductive limit topology consists of Radon charges, that is, the maps $\Phi :C_c(X)\rightarrow \mathbb {C}$ such that there is a unique positive Borel measure *m* and a Borel-measurable map $u:X\rightarrow \mathbb {T}$ with

$$\begin{aligned} \Phi (\varphi )=\int _{X}\varphi u\,dm \end{aligned}$$

for all $\varphi \in C_c(X)$. For such $\Phi$ and $C\in {{\,\mathrm{Cl}\,}}(X)$, set

$$\begin{aligned} (\Phi \wedge C)(\varphi )=\int _C \varphi u\,dm \end{aligned}$$

for each $\varphi \in C_c(X)$. Then, the new functional $\Phi \wedge C$ is a Radon charge. With this operation $C_c(X)^*\times {{\,\mathrm{Cl}\,}}(X)\ni (\Phi ,C)\mapsto \Phi \wedge C\in C_c(X)^*$, the space $C_c(X)^*$ becomes an abstract pattern space over *X*.

Note that, if *m* is a positive measure on *X* and $u:X\rightarrow \mathbb {C}$ is a bounded Borel map (not necessarily $\mathbb {T}$-valued), then $\Phi :C_c(X)\ni \varphi \mapsto \int \varphi u\,dm$ is a Radon charge. If $C\in {{\,\mathrm{Cl}\,}}(X)$, then

$$\begin{aligned} (\Phi \wedge C)(\varphi )=\int _C\varphi u\,dm \end{aligned}$$

for each $$\varphi \in C_c(X)$$.

Note also that, if *X* is second-countable and $\mu$ is a positive measure, the topological support of $\mu$ coincides with the support of the functional $C_c(X)\ni \varphi \mapsto \int \varphi \, d\mu$ as an abstract pattern.

Next we investigate abstract pattern subspaces. The relation between an abstract pattern space and its abstract pattern subspaces is similar to that between a set with a group action and its invariant subsets.

#### Definition 2.18

Let $\Pi$ be an abstract pattern space over *X*. Suppose that a nonempty subset $\Pi '$ of $\Pi$ satisfies the condition

$$\begin{aligned} \mathcal {P}\in \Pi '\text { and }C\in {{\,\mathrm{Cl}\,}}(X)\Rightarrow \mathcal {P}\wedge C\in \Pi '. \end{aligned}$$

Then $\Pi '$ is called an *abstract pattern subspace* of $\Pi$.

#### Remark 2.19

If $\Pi '$ is an abstract pattern subspace of an abstract pattern space $\Pi$, then $\Pi '$ becomes an abstract pattern space by restricting the cutting-off operation.

#### Example 2.20

Let *X* be a topological space. Then ${{\,\mathrm{Cl}\,}}(X)$ is an abstract pattern subspace of $2^X$. If *X* is a metric space, then ${{\,\mathrm{LF}\,}}(X)$ is an abstract pattern subspace of ${{\,\mathrm{Cl}\,}}(X)$ and ${{\,\mathrm{UD}\,}}_r(X)$ is an abstract pattern subspace of ${{\,\mathrm{UD}\,}}(X)$ for each $r>0$. Since we assume that the metrics we consider are proper, ${{\,\mathrm{UD}\,}}(X)$ is an abstract pattern subspace of ${{\,\mathrm{LF}\,}}(X)$.

Next we investigate a way to construct new abstract pattern spaces from old ones, taking a product.

#### Lemma 2.21

Let $\Lambda$ be an index set and $\Pi _{\Lambda },\lambda \in \Lambda$ a family of abstract pattern spaces over *X*. The direct product $\prod _{\lambda }\Pi _{\lambda }$ becomes an abstract pattern space over *X* with the cutting-off operation defined via

$$\begin{aligned} (\mathcal {P}_{\lambda })_{\lambda \in \Lambda }\wedge C=(\mathcal {P}_{\lambda }\wedge C)_{\lambda \in \Lambda } \end{aligned}$$

for $(\mathcal {P}_{\lambda })_{\lambda }\in \prod _{\lambda }\Pi _{\lambda }$ and $C\in {{\,\mathrm{Cl}\,}}(X)$. The support is given by ${{\,\mathrm{supp}\,}}(\mathcal {P}_{\lambda })_{\lambda }=\overline{\bigcup _{\lambda }{{\,\mathrm{supp}\,}}\mathcal {P}_{\lambda }}$.

#### Definition 2.22

Under the same condition as in Lemma 2.21, we call $\prod \Pi _{\lambda }$ the *product abstract pattern space* of $(\Pi _{\lambda })_{\lambda }$.

This construction of product abstract pattern spaces will be used in Proposition 4.28. The following construction of a Delone multiset, which uses product, is also essential.

#### Example 2.23

(*Uniformly discrete multiset* [9]) Let *I* be a set. Consider the abstract pattern subspace ${{\,\mathrm{UD}\,}}^I(X)$ of $\prod _{i\in I}{{\,\mathrm{UD}\,}}(X)$, defined via

$$\begin{aligned} {{\,\mathrm{UD}\,}}^I(X)=\Bigl \{(D_i)_{i\in I} \ {\big |} \ \bigcup _i D_i\in {{\,\mathrm{UD}\,}}(X) \Bigr \}. \end{aligned}$$

Elements of ${{\,\mathrm{UD}\,}}^I(X)$ are called uniformly discrete multisets. A uniformly discrete multiset $(D_i)_i\in {{\,\mathrm{UD}\,}}^I(X)$ is called a Delone multiset if each $D_i$ is a Delone set.

Next, we define a notion which will be useful later. In this article, we mainly deal with abstract patterns such that the sizes of the “components” of each abstract pattern are bounded from above. For example, we do not deal with tilings of ${\mathbb {R}}^d$ that contain translates of $(0,n)^d$ for all $n=1,2,\dots$ In such a tiling $\mathcal {T}$, if we fix $R>0$ and vary $x\in {\mathbb {R}}^d$, the cutting-offs $\mathcal {T}\wedge B(x,R)$ do not contain translates of $(0,n)^d$, for large *n*. We use this observation to define abstract patterns with bounded components: these are abstract patterns $\mathcal {P}$ such that, if $R>0$ is large enough, then any “component” of $\mathcal {P}$ near $x\in X$ is contained in *B*(*x*, *R*), so that if $x\in {{\,\mathrm{supp}\,}}\mathcal {P}$, then $x\in {{\,\mathrm{supp}\,}}(\mathcal {P}\wedge B(x,R))$. The above $\mathcal {T}$ does *not* consist of bounded components. Technically, we have to consider the same condition for any $\mathcal {P}\wedge C$ for $C\in {{\,\mathrm{Cl}\,}}(X)$, and the definition is given below.

Maps and elements of $2^X$ (and so uniformly discrete subsets of *X*) always satisfy this condition; a patch (and so a tiling) satisfies this condition if and only if the diameters of tiles in that patch are bounded from above.

#### Definition 2.24

Let $\Pi$ be an abstract pattern space over a metric space *X*. For any element $\mathcal {P}\in \Pi$, we say $\mathcal {P}$*consists of bounded components* if there is $R_{\mathcal {P}}>0$ such that, for any $C\in {{\,\mathrm{Cl}\,}}(X)$ and $x\in {{\,\mathrm{supp}\,}}(\mathcal {P}\wedge C)$, we have $x\in {{\,\mathrm{supp}\,}}(\mathcal {P}\wedge C\wedge B(x,R_{\mathcal {P}}))$.

### $\Gamma$-Abstract Pattern Spaces over *X*, or Abstract Pattern Spaces over $(X,\Gamma )$

Here, we incorporate group actions into the theory of abstract pattern spaces. First, we define abstract pattern spaces over $(X,\Gamma )$, or $\Gamma$-abstract pattern spaces over *X*, where *X* is a topological space and a group $\Gamma$ acts on *X* by homeomorphisms. We require that there is an action of the group $\Gamma$ on such an abstract pattern space and that the cutting-off operation is equivariant. In the next subsection, we define local derivation by using the structure of $\Gamma$-abstract pattern spaces.

#### Setting 1

In this subsection, unless otherwise stated, *X* is a topological space, $\Gamma$ is a group that acts on *X* as homeomorphisms, and $\Pi$ is an abstract pattern space over *X*.

#### Definition 2.25

Given a group action $\Gamma \curvearrowright \Pi$ such that, for each $\mathcal {P}\in \Pi , C\in {{\,\mathrm{Cl}\,}}(X)$ and $\gamma \in \Gamma$, we have $(\gamma \mathcal {P})\wedge (\gamma C)=\gamma (\mathcal {P}\wedge C)$, that is, the cutting-off operation is equivariant, we say $\Pi$ is a $\Gamma$*-abstract pattern space* or an *abstract pattern space over*$(X,\Gamma )$.

For an abstract pattern space $\Pi$ over $(X,\Gamma )$, a nonempty subset $\Sigma$ of $\Pi$ such that $\mathcal {P}\in \Sigma$ and $\gamma \in \Gamma$ imply $\gamma \mathcal {P}\in \Sigma$ is called a *subshift* of $\Pi$.

Examples are given after the next few lemmas. First, we describe the relation between the support and the group action $\Gamma \curvearrowright \Pi$.

#### Lemma 2.26

Let $\Pi$ be an abstract pattern space over $(X,\Gamma )$. For $\mathcal {P}\in \Pi$ and $\gamma \in \Gamma$, then $\gamma {{\,\mathrm{supp}\,}}\mathcal {P}={{\,\mathrm{supp}\,}}(\gamma \mathcal {P})$.

Next, we prove a lemma on taking products of $\Gamma$-abstract pattern spaces.

#### Lemma 2.27

Let $\Lambda$ be a set and $(\Pi _{\lambda })_{\lambda \in \Lambda }$ be a family of abstract pattern spaces over $(X,\Gamma )$. Then $\Gamma$ acts on the product space $\prod _{\lambda }\Pi _{\lambda }$ by $\gamma (\mathcal {P}_{\lambda })_{\lambda }=(\gamma \mathcal {P}_{\lambda })_{\lambda }$, and by this action $\prod _{\lambda }\Pi _{\lambda }$ is an abstract pattern space over $(X,\Gamma )$.

#### Proof

That $\prod \Pi _{\lambda }$ is an abstract pattern space is proved in Lemma 2.21. For $\gamma \in \Gamma , (\mathcal {P}_{\lambda })\in \prod \Pi _{\lambda }$, and $C\in {{\,\mathrm{Cl}\,}}(X)$, $\gamma ((\mathcal {P}_{\lambda })_{\lambda }\wedge C)=(\gamma (\mathcal {P}_{\lambda })_{\lambda })\wedge \gamma C$ by a straightforward computation. $\square$

#### Definition 2.28

The abstract pattern space $\prod \Pi _{\lambda }$ is called the *product*$\Gamma$*-abstract pattern space*.

We use the structure of $\Gamma$-abstract pattern spaces on the product in Proposition 4.28.

We now collect examples of abstract pattern spaces over $(X,\Gamma )$.

#### Example 2.29

Suppose *X* is a metric space and the action $\Gamma \curvearrowright X$ is isometric. For $\mathcal {P}\in {{\,\mathrm{Patch}\,}}(X)$ and $\gamma \in \Gamma$, set $\gamma \mathcal {P}=\{\gamma T\,{|}\, T\in \mathcal {P}\}$. This defines an action of $\Gamma$ on ${{\,\mathrm{Patch}\,}}(X)$ and makes ${{\,\mathrm{Patch}\,}}(X)$ an abstract pattern space over $(X,\Gamma )$.

#### Example 2.30

Let *X* be a metric space and let a group $\Gamma$ act on *X* as isometries. $2^X$ (Example 2.15) is an abstract pattern space over $(X,\Gamma )$. Similarly, the spaces ${{\,\mathrm{LF}\,}}(X)$ (Example 2.13), ${{\,\mathrm{UD}\,}}(X)$, and ${{\,\mathrm{UD}\,}}_r(X)$ (Example 2.14, $r>0$) are all abstract pattern spaces over $(X,\Gamma )$.

By taking the product of ${{\,\mathrm{UD}\,}}(X)$’s, we see that the abstract pattern space ${{\,\mathrm{UD}\,}}^I(X)$ of uniformly discrete multisets (Example 2.23) is a $\Gamma$-abstract pattern space and that the space of all Delone multiset is its subshift.

Next, we define a group action on ${{\,\mathrm{Map}\,}}(X,Y,y_0)$ (Example 2.16) that makes it a $\Gamma$-abstract pattern space. We can just define $(\gamma f)(x)=f(\gamma ^{-1}x)$ for $f\in {{\,\mathrm{Map}\,}}(X,Y,y_0), \gamma \in \Gamma$, and $x\in X$, but later we also need an action with “twisting,” which is defined as follows. This anticipates an application of our theory to the theory of pattern-equivariant functions, which were defined in [7] and [11]. By this group action, pattern equivariance becomes equivalent to local derivability. See Sect. 3 and Sect. 5.

#### Example 2.31

Take a nonempty set *Y*, an element $y_0\in Y$, and an action $\phi :\Gamma \curvearrowright Y$ that fixes $y_0$. As mentioned above (Example 2.16), ${{\,\mathrm{Map}\,}}(X,Y,y_0)$ is an abstract pattern space over *X*. Define an action of $\Gamma$ on ${{\,\mathrm{Map}\,}}(X,Y,y_0)$ by

$$\begin{aligned} (\gamma f)(x)=\phi (\gamma )(f(\gamma ^{-1}x)). \end{aligned}$$

For each $f\in {{\,\mathrm{Map}\,}}(X,Y,y_0)$, $\gamma \in \Gamma$, and $C\in {{\,\mathrm{Cl}\,}}(X)$,

$$\begin{aligned} (\gamma f)\wedge (\gamma C)(x)=&{\left\{ \begin{array}{ll} (\gamma f)(x)&{}\text {if }x\in \gamma C,\\ y_0 &{}\text {otherwise} \end{array}\right. }\\ =&{\left\{ \begin{array}{ll} \phi (\gamma )(f(\gamma ^{-1}x))&{} \text {if }\gamma ^{-1}x\in C,\\ \phi (\gamma )y_0&{}\text {otherwise} \end{array}\right. }\\ =&\;\phi (\gamma )(f\wedge C)(\gamma ^{-1}x)\\ =&\;\gamma (f\wedge C)(x), \end{aligned}$$

for each $x\in X$, so ${{\,\mathrm{Map}\,}}(X,Y,y_0)$ is an abstract pattern space over $(X,\Gamma )$. This $\Gamma$-abstract pattern space is denoted by ${{\,\mathrm{Map}\,}}_{\phi }(X,Y,y_0)$. If $\phi$ sends every group element to the identity, we denote the corresponding space by ${{\,\mathrm{Map}\,}}(X,Y,y_0)$.

We also construct the abstract pattern spaces of measures of $\Gamma$-abstract pattern spaces.

#### Example 2.32

Let *X* be a locally compact $\sigma$-compact space and let a group $\Gamma$ act on *X* as homeomorphisms. The dual space $C_c(X)^{*}$ with respect to the inductive limit topology is an abstract pattern space over *X* (Example 2.17). For $\varphi \in C_c(X)$ and $\gamma \in \Gamma$, set $(\gamma \varphi )(x)=\varphi (\gamma ^{-1}x)$. For $\Phi \in C_c(X)^*$ and $\gamma \in \Gamma$, set $\gamma \Phi (\varphi )=\Phi (\gamma ^{-1}\varphi )$. Then $C_c(X)^{*}$ is an abstract pattern space over $(X,\Gamma )$.

We have introduced various examples of abstract pattern spaces over $(X,\Gamma )$. Next, we mention three examples of subshifts.

#### Example 2.33

For a (proper) metric space *X*, the set ${{\,\mathrm{Del}\,}}(X)$ of all Delone sets in *X* (Example 2.14) is a subshift of ${{\,\mathrm{UD}\,}}(X)$.

#### Example 2.34

For a metric space *X*, the space of all tilings is a subshift of ${{\,\mathrm{Patch}\,}}(X)$.

#### Example 2.35

In Example 2.31, assume that *Y* is a topological space and that each $\phi (\gamma )$ is continuous. The space *C*(*X*, *Y*) of all continuous maps is a subshift of ${{\,\mathrm{Map}\,}}_{\phi }(X,Y,y_0)$. This is not necessarily an abstract pattern subspace, since after cutting off, a continuous function may become discontinuous and thus leave *C*(*X*, *Y*).

### Local Derivability

#### Setting 2

In this subsection, *X*, *Y*, and *Z* are nonempty proper metric spaces and $\Gamma$ is a group which acts on *X*, *Y*, and *Z* as isometries.

Local derivability was defined in [4] for tilings or more generally for patterns in ${\mathbb {R}}^d$. Here, we define local derivability for two abstract patterns $\mathcal {P}_1$ and $\mathcal {P}_2$. Note that these $\mathcal {P}_1$ and $\mathcal {P}_2$ may be in different abstract pattern spaces $\Pi _1$ and $\Pi _2$, and these $\Pi _1$ and $\Pi _2$ may be over different metric spaces *X* and *Y*. However, we assume that $\Pi _1$ and $\Pi _2$ are $\Gamma$-abstract pattern spaces for the same group $\Gamma$.

Our definition is equivalent to the original definition in [4] (see also [2], p. 133) for patterns under an assumption (Lemma 2.37).

#### Definition 2.36

Let $\Pi _1$ be an abstract pattern space over $(X,\Gamma )$ and $\Pi _2$ be an abstract pattern space over $(Y,\Gamma )$. Take two abstract patterns $\mathcal {P}_1\in \Pi _1$ and $\mathcal {P}_2\in \Pi _2$. If for any compact $K\subset Y$, there is a compact $K'\subset X$ such that $\gamma ,\eta \in \Gamma$ and

$$\begin{aligned} (\gamma \mathcal {P}_1)\wedge K'=(\eta \mathcal {P}_1)\wedge K' \end{aligned}$$

imply

$$\begin{aligned} (\gamma \mathcal {P}_2)\wedge K=(\eta \mathcal {P}_2)\wedge K, \end{aligned}$$

then we say $\mathcal {P}_2$ is *locally derivable from*$\mathcal {P}_1$ and write $\mathcal {P}_1\overset{{\mathrm {LD}}}{\rightarrow }\mathcal {P}_2$. If both $\mathcal {P}_1\overset{{\mathrm {LD}}}{\rightarrow }\mathcal {P}_2$ and $\mathcal {P}_2\overset{{\mathrm {LD}}}{\rightarrow }\mathcal {P}_1$ hold, we say $\mathcal {P}_1$ and $\mathcal {P}_2$ are *mutually locally derivable* (MLD) and write $\mathcal {P}_1\overset{{\mathrm {MLD}}}{\leftrightarrow }\mathcal {P}_2$.

We next prove that, under a mild assumption, our definition of local derivability (LD) is equivalent to the original one in [4].

#### Lemma 2.37

Assume that the action $\Gamma \curvearrowright X$ is transitive. Take $x\in X$ and two patterns $\mathcal {P}_1,\mathcal {P}_2\in {{\,\mathrm{Pattern}\,}}(X)$ (Example 2.11). Assume $\sup _{T\in \mathcal {P}_2}{{\,\mathrm{diam}\,}}T<\infty$. Then the following two conditions are equivalent:

- $$\mathcal {P}_1\overset{{\mathrm {LD}}}{\rightarrow }\mathcal {P}_2$$.
- There exists a compact $K\subset X$ such that, if $\gamma ,\eta \in \Gamma$ and $(\gamma \mathcal {P}_1)\sqcap K=(\eta \mathcal {P}_1)\sqcap K$, then $(\gamma \mathcal {P}_2)\sqcap \{x\}=(\eta \mathcal {P}_2)\sqcap \{x\}$.

#### Proof

Take $L>\sup _{T\in \mathcal {P}_2}{{\,\mathrm{diam}\,}}T$. We first assume condition (a) and prove condition (b). Take a compact $K\subset X$. If $\gamma ,\eta \in \Gamma$ and

$$\begin{aligned} (\gamma \mathcal {P}_1)\sqcap K=(\eta \mathcal {P}_1)\sqcap K, \end{aligned}$$

then

$$\begin{aligned} (\gamma \mathcal {P}_1)\wedge K=(\eta \mathcal {P}_1)\wedge K. \end{aligned}$$

If *K* is large enough, this implies that

$$\begin{aligned} (\gamma \mathcal {P}_2)\wedge B(x,L)=(\eta \mathcal {P}_2)\wedge B(x,L). \end{aligned}$$

By the definition of *L*, we have $(\gamma \mathcal {P}_2)\sqcap \{x\}=(\eta \mathcal {P}_2)\sqcap \{x\}$.

Next, we assume condition (b) and prove condition (a). There exists *K* as in condition (b). Since *K* is compact, we can take $R_0\geqq 0$ such that $K\subset B(x,R_0)$. To prove condition (a), take a compact subset of *X*. We may assume that it is of the form *B*(*x*, *R*) for some $R\geqq 0$. If $\gamma ,\eta \in \Gamma$ and $(\gamma \mathcal {P}_1)\wedge B(x,R_0+L+R)=(\eta \mathcal {P}_1)\wedge B(x,R_0+L+R)$, then since the action is transitive, for each $y\in B(x,R)$, there exists $\xi \in \Gamma$ such that $\xi x=y$. By $B(x,R_0+L)\subset B(\xi ^{-1}x,R_0+L+R)$, we have

$$\begin{aligned} (\xi ^{-1}\gamma \mathcal {P}_1)\wedge B(x,R_0+L)=(\xi ^{-1}\eta \mathcal {P}_1)\wedge B(x,R_0+L) \end{aligned}$$

and

$$\begin{aligned} (\xi ^{-1}\gamma \mathcal {P}_1)\sqcap K=(\xi ^{-1}\eta \mathcal {P}_1)\sqcap K. \end{aligned}$$

By the definition of *K*, we have

$$\begin{aligned} (\xi ^{-1}\gamma \mathcal {P}_2)\sqcap \{x\}=(\xi ^{-1}\eta \mathcal {P}_2)\sqcap \{x\} \end{aligned}$$

and $(\gamma \mathcal {P}_2)\sqcap \{y\}=(\eta \mathcal {P}_2)\sqcap \{y\}$. Since *y* is arbitrary, we have $(\gamma \mathcal {P}_2)\sqcap B(x,R)=(\eta \mathcal {P}_2)\sqcap B(x,R)$ and $(\gamma \mathcal {P}_2)\wedge B(x,R)=(\eta \mathcal {P}_2)\wedge B(x,R)$. $\square$

#### Corollary 2.38

Suppose that the action $\Gamma \curvearrowright X$ is transitive and $\mathcal {P}_1$ and $\mathcal {P}_2$ are patches in *X*. If $\mathcal {P}_2$ consists of bounded components, then our definition of $\mathcal {P}_1\overset{{\mathrm {LD}}}{\rightarrow }\mathcal {P}_2$ coincides with the original definition (condition (b) in Lemma 2.37).

#### Proof

A patch $\mathcal {P}$ consists of bounded components iff $\sup _{T\in \mathcal {P}}{{\,\mathrm{diam}\,}}T<\infty$. $\square$

The following two lemmas are easy to prove. First we show that $\overset{{\mathrm {MLD}}}{\leftrightarrow }$ is an equivalence relation.

#### Lemma 2.39

1. Let $\mathcal {P}$ be an abstract pattern in an abstract pattern space over $(X,\Gamma )$. Then $\mathcal {P}\overset{{\mathrm {MLD}}}{\leftrightarrow }\mathcal {P}$.
2. Let $\mathcal {P},\mathcal {Q}$ and $\mathcal {R}$ be abstract patterns in abstract pattern spaces over $(X,\Gamma )$, $(Y,\Gamma )$, and $(Z,\Gamma )$, respectively. If $\mathcal {P}\overset{{\mathrm {LD}}}{\rightarrow }\mathcal {Q}$ and $\mathcal {Q}\overset{{\mathrm {LD}}}{\rightarrow }\mathcal {R}$, then $\mathcal {P}\overset{{\mathrm {LD}}}{\rightarrow }\mathcal {R}$. Consequently, if $\mathcal {P}\overset{{\mathrm {MLD}}}{\leftrightarrow }\mathcal {Q}$ and $\mathcal {Q}\overset{{\mathrm {MLD}}}{\leftrightarrow }\mathcal {R}$, then $\mathcal {P}\overset{{\mathrm {MLD}}}{\leftrightarrow }\mathcal {R}$.

Next, we investigate a relation between $\overset{{\mathrm {LD}}}{\rightarrow }$ and the group action $\Gamma \curvearrowright \Pi$.

#### Lemma 2.40

Let $\Pi _1$ be an abstract pattern space over $(X,\Gamma )$ and $\Pi _2$ be an abstract pattern space over $(Y,\Gamma )$. Take two abstract patterns $\mathcal {P}_1\in \Pi _1$ and $\mathcal {P}_2\in \Pi _2$ and suppose $\mathcal {P}_1\overset{{\mathrm {LD}}}{\rightarrow }\mathcal {P}_2$. Then, for any $\gamma \in \Gamma$, we have $\gamma \mathcal {P}_1\overset{{\mathrm {LD}}}{\rightarrow }\gamma \mathcal {P}_2$.

We finish this subsection by showing that several canonical maps in aperiodic order send an abstract pattern $\mathcal {P}$ to one which is MLD with $\mathcal {P}$. This is important because, in many cases, the local matching uniform structures, which are defined for abstract pattern spaces over $(X,\Gamma )$, are complete [10], so MLD implies topological conjugacy between the corresponding dynamical systems.

It is common to convert a Delone set into the Dirac comb, that is, the measure consisting of Dirac measure on each point [2, Ex. 8.6]. We show that these abstract patterns are MLD.

#### Proposition 2.41

Let *X* be a locally compact proper metric space on which a group $\Gamma$ acts as isometries. Let *D* be a uniformly discrete subset of *X* and set $\mu =\sum _{x\in D}\delta _x$ (the Dirac comb, the convergence is with respect to the vague topology). If we regard *D* as an abstract pattern of ${{\,\mathrm{UD}\,}}(X)$ (Example 2.30) and $\mu$ as an abstract pattern of $C_c(X)^{*}$ (Example 2.32), we have the following:

1. $$\mu \wedge C=\sum _{x\in D\cap C}\delta _x$$ for each $$C\in {{\,\mathrm{Cl}\,}}(X)$$,
2. $$\gamma \mu =\sum _{x\in \gamma D}\delta _x$$, and
3. $$\mu \overset{{\mathrm {MLD}}}{\leftrightarrow }D$$.

#### Proof

The first two are clear by definition, and the third condition follows from the first two conditions. $\square$

It is common to identify a continuous bounded function *f* on a locally compact Abelian group and $f\,d\mu$, $\mu$ being a Haar measure. See for example [3, Prop. 4.10.5, Lem. 5.4.6]. We show that these are MLD.

#### Proposition 2.42

Let $\Gamma$ be a $\sigma$-compact locally compact Abelian group and $\mu$ its Haar measure. Let *f* be a complex-valued continuous bounded function on $\Gamma$. If we regard *f* as an abstract pattern in ${{\,\mathrm{Map}\,}}(\Gamma ,\mathbb {C},0)$ (Example 2.31) and $f\,d\mu$ as an element of $C_c(\Gamma )^*$ (Example 2.32) that sends $\varphi \in C_c(\Gamma )$ to $\int \varphi f\,d\mu$, we have $f\overset{{\mathrm {MLD}}}{\leftrightarrow }f\,d\mu$.

#### Proof

Take $R>0$ and $s,t\in \Gamma$ and assume

4 $$\begin{aligned} (f-s)\wedge B(e,R)=(f-t)\wedge B(e,R). \end{aligned}$$

Here, $f-t$ and $f-s$ denote the image of *f* by the group action. For each $\varphi \in C_c(\Gamma )$, the image by $(f\,d\mu -s)\wedge B(e,R)$ is $\int _{B(e,R)}\varphi (x)f(x+s)\,d\mu$ and the image by $(f\,d\mu -t)\wedge B(e,R)$ is $\int _{B(e,R)}\varphi (x)f(x+t)\,d\mu$. By (4), for each $x\in B(e,R)$,

$$\begin{aligned} f(x+t)= & {} (f\wedge B(t,R))(x+t)=((f-t)\wedge B(0,R))(x)\\= & {} ((f-s)\wedge B(e,R))(x)=f(x+s), \end{aligned}$$

so the images of $\varphi$ by $(f\,d\mu -s)\wedge B(e,R)$ and $(f\,d\mu -t)\wedge B(e,R)$ are the same, and thus these two maps are the same.

Conversely, suppose $R>0$, $s,t\in \Gamma$, and

$$\begin{aligned} (f\,d\mu -s)\wedge B(e,R+1)=(f\,d\mu -t)\wedge B(e,R+1). \end{aligned}$$

For any $\varphi \in C_c(\Gamma )$ with ${{\,\mathrm{supp}\,}}\varphi \subset B(e,R+1)$, we have

$$\begin{aligned} \int \varphi (x)f(x+s)\,d\mu (x)=\int \varphi (x)f(x+t)\,d\mu (x), \end{aligned}$$

so for any $x\in B(e,R)$, we have $f(x+s)=f(x+t)$ and

$$\begin{aligned} (f-s)\wedge B(e,R)=(f-t)\wedge B(e,R). \square \end{aligned}$$

For the rest of this subsection, $({\mathbb {R}}^d,\rho )$ is the Euclidean space with the Euclidean metric and *D* is a Delone subset (Example 2.14) of ${\mathbb {R}}^d$ which is *R*-relatively dense and *r*-uniformly discrete for some $R,r>0$.

It is sometimes useful to convert *D* in ${\mathbb {R}}^d$ into a tiling. This is done by constructing Voronoi cells and Voronoi tilings [12]. The set $V_x$ below (or its closure) is called the Voronoi cell of *D* at *x*. The set of all the Voronoi cells $V_x$, $x\in D$, forms a tiling called a Voronoi tiling or Voronoi tessellation, but the original Delone set *D* is not necessarily locally derivable from the tiling. For example, consider the Delone set $D=\bigl \{a+n\,{|}\, a\in \bigl \{\frac{1}{5},-\frac{1}{5}\bigr \},n\in \mathbb {Z}\bigr \}$ in $\mathbb {R}$. The set of all $V_x$’s forms a tiling $\bigl \{\bigl (0,\frac{1}{2}\bigr )+n\,{|}\, n\in \frac{1}{2}\mathbb {Z}\bigr \}$, but the symmetry group of the tiling is $\frac{1}{2}\mathbb {Z}$, which is strictly larger than the symmetry group $\mathbb {Z}$ of the original *D*. The symmetry group is preserved under MLD, so *D* and this tiling are not MLD. We circumvent this problem by considering punctured Voronoi cells $U_x$.

Although the construction is well known, we do not omit it and prove MLD with or without rotation.

#### Definition 2.43

For each $x\in D$, we denote by $V_x$ the set

$$\begin{aligned} V_x=\bigl \{y\in {\mathbb {R}}^d\mid \rho (x,y)<\rho (x',y)\text { for any }x'\in D\setminus \{x\}\bigr \}. \end{aligned}$$

#### Lemma 2.44

For each $x\in D$, $V_x$ is nonempty and $V_x\subset B(x,R)^{\circ }$. Moreover,

5 $$\begin{aligned} V_x=\bigl \{y\in B(x,R)^{\circ }\mid \rho (x,y)<\rho (x',y)\text { for each }x'\in D'\bigr \} \end{aligned}$$

for each $D'$ with $D\setminus \{x\}\cap B(x,2R)\subset D' \subset D\setminus \{x\}$. In particular, $V_x$ is open for each $x\in D$.

#### Proof

Since $x\in V_x$, $V_x\ne \emptyset$. If $y\in {\mathbb {R}}^d\setminus B(x,R)^{\circ }$, then since there is $x'\in D\cap B(y,R)^{\circ }$, we have $\rho (x',y)<R\leqq \rho (x,y)$ and so $y\notin V_x$.

Assume $y\in B(x,R)^{\circ }$ and $\rho (x,y)<\rho (x',y)$ for each $x'\in (D\setminus \{x\})\cap B(x,2R)$. If $x'\in D\setminus \{x\}$ and $\rho (x,x')>2R$, then $\rho (x',y)\geqq \rho (x,x')-\rho (x,y)>R>\rho (x,y)$ and so $y\in V_x$. This observation shows the equality (5). $\square$

#### Definition 2.45

For each $x\in D$, set $U_x=V_x\setminus \{x\}$. Set $\mathcal {T}=\{U_x\,{|}\, x\in D\}$.

#### Lemma 2.46

$\mathcal {T}$ is a tiling of ${\mathbb {R}}^d$.

#### Proof

By Lemma 2.44, $U_x$ is open, bounded, and nonempty. By the definition of $V_x$, if $x\ne x'$ we have $U_x\cap U_{x'}=\emptyset$. Next, we take $y\in {\mathbb {R}}^d$ and show that there is $x\in D$ such that $y\in \overline{U_x}$. To this purpose, we may assume that $y\ne x$ for any $x\in D$. Since $\{x\in D\,{|}\, \rho (x,y)<R\}$ is finite and nonempty, $F=\{x\in D\,{|}\, \rho (x,y)\leqq \rho (x',y)\text { for any }x'\in D\}$ is nonempty and finite. Take $x\in F$. For each $t\in (0,1)$, set $y_t=tx+(1-t)y$. Then $\rho (x,y_t)=\Vert (1-t)(y-x)\Vert$. If $x'\in D$ and $\{y-x,y-x'\}$ is linearly independent, we have

$$\begin{aligned} \rho (x',y_t)&=\Vert (1-t)y+tx-x'\Vert >\Vert y-x'\Vert -t\Vert y-x\Vert \\&\geqq (1-t)\Vert y-x\Vert =\rho (x,y_t). \end{aligned}$$

If $x'\in D\setminus \{x\}$ and $\{y-x,y-x'\}$ is linearly dependent, then there is $\lambda \in \mathbb {R}$ such that $x'-y=\lambda (x-y)$. Since $\lambda >1$ or $\lambda \leqq -1$, we see that $\rho (y_t,x)<\rho (y_t,x')$. By these observations, we see that $y_t\in V_x$, so $y\in \overline{V_x}=\overline{U_x}$. $\square$

#### Remark 2.47

There is $s>0$ such that $B(x,s)\subset U_x\cup \{x\}$. Conversely, if $y\in {\mathbb {R}}^d\setminus U_x$ and there is $s>0$ such that $B(y,s)\subset U_x\cup \{y\}$, then $x=y$. Thus, if $x,y\in D$, $\gamma ,\eta \in \Gamma$, and $\gamma U_x=\eta U_y$, then $\gamma x=\eta y$.

#### Proposition 2.48

Let $\Gamma$ be a closed subgroup of  $\mathrm{E}(d)$. If we regard *D* as an element of ${{\,\mathrm{UD}\,}}({\mathbb {R}}^d)$, which is an abstract pattern space over $({\mathbb {R}}^d,\Gamma )$, and $\mathcal {T}$ as an element of ${{\,\mathrm{Patch}\,}}({\mathbb {R}}^d)$, which is also an abstract pattern space over $({\mathbb {R}}^d,\Gamma )$, we have $D\overset{{\mathrm {MLD}}}{\leftrightarrow }\mathcal {T}$.

#### Proof

Take $L>0$ and $\gamma ,\eta \in \Gamma$ and assume

6 $$\begin{aligned} (\gamma D)\cap B(0,L+2R)=(\eta D)\cap B(0,L+2R). \end{aligned}$$

Suppose $x\in D$ and $\gamma U_x\subset B(0,L)$. Since $\gamma x\in B(0,L)$, by (6), we see $\gamma x\in \eta D$ and $y=\eta ^{-1}\gamma x\in D$. By setting $D'=(D\setminus \{x\})\cap B(\gamma ^{-1}0,L+2R)$ in Lemma 2.44, we have

$$\begin{aligned} \gamma U_x= & {} \gamma \bigl \{z\in B(x,R)^{\circ }\mid \rho (x,z)<\rho (x',z)\\&\qquad \qquad \qquad \qquad \qquad \text { for any } x'\in (D\setminus \{x\})\cap B(\gamma ^{-1}0,L+2R)\bigr \}\\= & {} \bigl \{z\in B(\gamma x,R)^{\circ }\mid \rho (\gamma x,z)<\rho (x',z)\\&\qquad \qquad \qquad \qquad \qquad \text { for any } x'\in '(\gamma D)\cap B(0,L+2R)\setminus \{\gamma x\}\bigr \}\\= & {} \bigl \{z\in B(\eta y,R)^{\circ }\mid \rho (\eta y,z)<\rho (x',z)\\&\qquad \qquad \qquad \qquad \qquad \text { for any } x'\in (\eta D)\cap B(0,L+2R)\setminus \{\eta y\})\bigr \}\\= & {} \eta U_y, \end{aligned}$$

so $\gamma U_x\in \eta \mathcal {T}$. We have shown that $(\gamma \mathcal {T})\wedge B(0,L)\subset \eta \mathcal {T}$, and by symmetry this implies that $(\gamma \mathcal {T})\wedge B(0,L)=(\eta \mathcal {T})\wedge B(0,L)$.

Conversely, assume $L>0$, $\eta ,\gamma \in \Gamma$, and

7 $$\begin{aligned} (\gamma \mathcal {T})\wedge B(0,L+R)=(\eta \mathcal {T})\wedge B(0,L+R). \end{aligned}$$

If $x\in D$ and $\gamma x\in B(0,L)$, then $\gamma U_x\subset B(0, L+R)$, so by (7) we have $\gamma U_x\in (\eta \mathcal {T})\wedge B(0,L+R)$. There is $y\in D$ such that $\gamma U_x=\eta U_y$, so $\gamma x=\eta y\in \eta D$. We have shown $(\gamma D)\cap B(0,L)\subset \eta D$, and by symmetry we obtain $(\gamma D)\cap B(0,L)=(\eta D)\cap B(0,L)$. $\square$

## A Study of Abstract Patterns via Arrows I: the Case without $\mathrm{O}(d)$-Actions

In this section, we study the theory of pattern-equivariant functions in terms of local derivability, by studying the graph with abstract patterns as vertices and local derivability as edges. We prove that the space of all pattern-equivariant functions contains all of the information on the original abstract pattern up to MLD (Theorem 3.6). The pattern-equivariant functions with $\mathrm{O}(d)$-action for abstract patterns in ${\mathbb {R}}^d$ will be studied in Sect. 5.

### The Role of Maximal Elements

We start with a definition in an abstract setting:

#### Definition 3.1

Let $\Pi$ be an abstract pattern space over $(X,\Gamma )$ and $\Pi '$ an abstract pattern space over $(Y,\Gamma )$, where $\Gamma$ is a group which acts on metric spaces *X* and *Y* respectively as isometries. Let $\Sigma$ be a subshift of $\Pi '$. For each $\mathcal {P}\in \Pi$, we set

$$\begin{aligned} \Sigma _{\mathcal {P}}=\big \{\mathcal {Q}\in \Sigma \,{|}\,\mathcal {P}\overset{{\mathrm {LD}}}{\rightarrow }\mathcal {Q}\big \}. \end{aligned}$$

In order to study the relations between $\mathcal {P}$ and $\Sigma _{\mathcal {P}}$, the maximal elements of $\Sigma _{\mathcal {P}}$, that is, the elements $\mathcal {Q}\in \Sigma$ such that $\mathcal {P}\overset{{\mathrm {MLD}}}{\leftrightarrow }\mathcal {Q}$, are useful, as the following lemma shows:

#### Lemma 3.2

Let $\Pi _j$ be an abstract pattern space over $(X_j,\Gamma )$, for each $j=1,2,3$, where $X_j$ is a metric space on which a group $\Gamma$ acts as isometries. Suppose that a subshift $\Sigma$ of $\Pi _3$ satisfies the following condition:

- for each $\mathcal {P}_1\in \Pi _1$, there is $\mathcal {P}_1'\in \Sigma$ such that $\mathcal {P}_1\overset{{\mathrm {MLD}}}{\leftrightarrow }\mathcal {P}_1'$, and
- for each $\mathcal {P}_2\in \Pi _2$, there is $\mathcal {P}_2'\in \Sigma$ such that $\mathcal {P}_2\overset{{\mathrm {MLD}}}{\leftrightarrow }\mathcal {P}_2'$.

Then, for each $\mathcal {P}_1\in \Pi _1$ and $\mathcal {P}_2\in \Pi _2$, we have $\mathcal {P}_1\overset{{\mathrm {MLD}}}{\leftrightarrow }\mathcal {P}_2$ if and only if $\Sigma _{\mathcal {P}_1}=\Sigma _{\mathcal {P}_2}$.

#### Proof

Take $\mathcal {P}_1\in \Pi _1$ and $\mathcal {P}_2\in \Pi _2$. There are $\mathcal {P}_1',\mathcal {P}_2'\in \Sigma$ as in the condition above. If $\mathcal {P}_1\overset{{\mathrm {LD}}}{\rightarrow }\mathcal {P}_2$, then for each $\mathcal {Q}\in \Sigma _{\mathcal {P}_2}$, we have $\mathcal {P}_1\overset{{\mathrm {LD}}}{\rightarrow }\mathcal {Q}$ by the transitivity of local derivability, so $\mathcal {Q}\in \Sigma _{\mathcal {P}_1}$. Thus, if $\mathcal {P}_1\overset{{\mathrm {MLD}}}{\leftrightarrow }\mathcal {P}_2$, then $\Sigma _{\mathcal {P}_1}=\Sigma _{\mathcal {P}_2}$. On the other hand, if $\Sigma _{\mathcal {P}_1}=\Sigma _{\mathcal {P}_2}$, then $\mathcal {P}_1'\in \Sigma _{\mathcal {P}_2}$ and so $\mathcal {P}_2\overset{{\mathrm {LD}}}{\rightarrow }\mathcal {P}_1'\overset{{\mathrm {LD}}}{\rightarrow }\mathcal {P}_1$. By transitivity, we have $\mathcal {P}_2\overset{{\mathrm {LD}}}{\rightarrow }\mathcal {P}_1$. Similarly $\mathcal {P}_1\overset{{\mathrm {LD}}}{\rightarrow }\mathcal {P}_2$, so $\mathcal {P}_1\overset{{\mathrm {MLD}}}{\leftrightarrow }\mathcal {P}_2$. $\square$

### Pattern-Equivariant Functions without $\mathrm{O}(d)$-Actions and Their Generalizations

Next, we move on to the theory of pattern-equivariant functions. We will show that, for certain $\Sigma$ consisting of functions, $\Sigma _{\mathcal {P}}$ is the space of pattern-equivariant functions. First, we recall the definition of pattern-equivariant functions. Kellendonk [7] defined pattern-equivariant functions for tilings or Delone sets in order to study the cohomology of the tiling spaces. We recall the definitions here.

#### Definition 3.3

[7] Let *D* be a subset of ${\mathbb {R}}^d$ and *Y* be a set. A function $f:{\mathbb {R}}^d\rightarrow Y$ is said to be (strongly) *D-equivariant* if there is $R>0$ such that $x,y\in {\mathbb {R}}^d$ and $(D-x)\cap B(0,R)=(D-y)\cap B(0,R)$ imply $f(x)=f(y)$.

It is easy to show that this definition can be rephrased in terms of local derivability:

#### Lemma 3.4

Let *D* be a subset of ${\mathbb {R}}^d$, *Y* be a set, and $y_0\in Y$. Then, for any $f\in {{\,\mathrm{Map}\,}}({\mathbb {R}}^d,Y)$, *f* is *D*-equivariant if and only if $D\overset{{\mathrm {LD}}}{\rightarrow }f$. Here, we regard *D* as an element of $2^X$ (Example 2.30), which is an abstract pattern space over $({\mathbb {R}}^d,{\mathbb {R}}^d)$, and of as an element of ${{\,\mathrm{Map}\,}}({\mathbb {R}}^d,Y,y_0)$ (Example 2.31), which is an abstract pattern space over $({\mathbb {R}}^d,{\mathbb {R}}^d)$.

We generalize the definition of pattern-equivariant function as follows:

#### Definition 3.5

Let *X* be a (proper) metric space and $\Gamma$ be a group which acts on *X* as isometries. Let $\mathcal {P}$ be an abstract pattern in an abstract pattern space over $(X,\Gamma )$. A function *f* in an abstract pattern space ${{\,\mathrm{Map}\,}}(X,Y,y_0)$ over $(X,\Gamma )$, where *Y* is a set and $y_0\in Y$ (Example 2.31), is said to be $\mathcal {P}$-equivariant if $\mathcal {P}\overset{{\mathrm {LD}}}{\rightarrow }f$.

For a subset $\Sigma$ of ${{\,\mathrm{Map}\,}}(X,Y,y_0)$, the set $\Sigma _{\mathcal {P}}$ (Definition 3.1) is the set of all $\mathcal {P}$-equivariant functions in $\Sigma$.

The following theorem, which states that the space of pattern-equivariant function is an MLD-invariant and remembers the original abstract pattern, is now easy to prove:

#### Theorem 3.6

Let *X* be a (proper) metric space on which a group $\Gamma$ acts transitively as isometries. Let $\mathcal {P}_1$ and $\mathcal {P}_2$ be abstract patterns in (possibly different) abstract pattern spaces over $(X,\Gamma )$. Assume there are uniformly discrete multisets $D^{(1)}$ and $D^{(2)}$ of *X* with finite color (Example 2.23) such that $\mathcal {P}_j\overset{{\mathrm {MLD}}}{\leftrightarrow }D^{(j)}$ for each *j*. Let $\Sigma =C(X,\mathbb {C})$ be a subshift consisting of continuous functions of the abstract pattern space ${{\,\mathrm{Map}\,}}(X,\mathbb {C},0)$ over $(X,\Gamma )$ (Example 2.31). Then $\mathcal {P}_1\overset{{\mathrm {MLD}}}{\leftrightarrow }\mathcal {P}_2$ if and only if $\Sigma _{\mathcal {P}_1}=\Sigma _{\mathcal {P}_2}$ (that is, the space of continuous pattern-equivariant functions coincide).

#### Proof

By Lemma 3.2, it suffices to show that, for each uniformly discrete multiset $D=(D_i)_{i\in I}$ in ${{\,\mathrm{UD}\,}}^I(X)$ with finite *I*, there is $f\in \Sigma$ such that $D\overset{{\mathrm {MLD}}}{\leftrightarrow }f$. We may assume $D_i\cap D_j=\emptyset$ if $i\ne j$, since otherwise we may replace *D* with a uniformly discrete multiset with this condition, that is MLD with *D*. There is $r>0$ such that, if $x,y\in \bigcup _i D_i$ and $x\ne y$, then $\rho (x,y)>2r$. Take $x_0\in X$ and a continuous function $\varphi :X\rightarrow [0,1]$ such that

1. $${{\,\mathrm{supp}\,}}\varphi \subset B(x_0,r)$$,
2. $$\varphi (x)=1\iff x=x_0$$, and
3. for each $\gamma \in \Gamma _{x_0}$ and $x\in X$, we have $\varphi (\gamma x)=\varphi (x)$.

Define a continuous function $f:X\rightarrow \mathbb {C}$ as follows. We use complex numbers $c_i\in \mathbb {T}$, for each $i\in I$, such that $c_i\ne c_j$ if $i\ne j$. Take an $x\in X$, and we should determine the value of *f* at *x*. If there are $i\in I$ and $\gamma \in \Gamma$ such that $\gamma x_0\in D_i$ and $\rho (\gamma x_0,x)<r$, then put $f(x)=c_i\varphi (\gamma ^{-1}x)$. Otherwise set $f(x)=0$. In other words, we put the copy of $c_i\varphi$ on each point of $D_i$. It is easy to show that *f* is continuous and $D\overset{{\mathrm {MLD}}}{\leftrightarrow }f$. $\square$

#### Remark 3.7

The space $C(X,\mathbb {C})$ may be replaced with the space of smooth functions from *X* to $\mathbb {C}$, whenever that makes sense. Often we can take smooth $\varphi$ in the proof, so that we may prove that the space of smooth pattern-equivariant functions remembers the original abstract pattern up to MLD.

## Translation Theorem for Certain Abstract Patterns

### Setting 3

In this section, *X* is a (proper) metric space. The metric is denoted by $\rho$.

The goal of this section is to state Theorem 4.40. Since the proof is long and technical, we postpone it to Appendix A.

In this section, we answer the following question: given an abstract pattern $\mathcal {P}$ and a set $\Sigma$ of abstract patterns, can we find an abstract pattern $\mathcal {Q}\in \Sigma$ such that $\mathcal {P}\overset{{\mathrm {MLD}}}{\leftrightarrow }\mathcal {Q}$? There are canonical conversion rules that are studied in Sect. 2.3. We can often find an ad hoc way to find such a $\mathcal {Q}$. In this section, we find a *general* condition for such $\mathcal {Q}$ to exist (Theorem 4.40). An application of this theorem will be found in Sect. 5.

Here is the strategy to construct such $\mathcal {Q}$. Let us explain how to construct a Delone set *D* that is MLD with rotation (S-MLD in literature) with the following tiling $\mathcal {P}$. Let *T* be a tile in ${\mathbb {R}}^d$ that is obtained by removing finitely many points from $(0,1)^d$, and $\mathcal {P}=\{T+x\,{|}\, x\in \mathbb {Z}^d\}$. The set *D* of all punctures of the tiles in $\mathcal {P}$ is a Delone set that is locally derivable from $\mathcal {P}$. Take $R>0$ that is large enough. For each $x\in D$, the patch $\mathcal {P}\wedge B(x,R)$ describes the local behavior of $\mathcal {P}$. These patches are copies, by Euclidean motions, of one of finitely many patches $\mathcal {P}_1,\mathcal {P}_2,\ldots ,\mathcal {P}_n$. For each $i=1,2,\ldots , n$, collect all Euclidean motions $\gamma$ such that $\gamma 0\in D$ and $\mathcal {T}\wedge B(\gamma 0,R)=\gamma \mathcal {P}_i$. Denote the set by $P_i$. The tuple $(P_i)_i$, which is called the plan, describes how we reconstruct $\mathcal {P}$ from components $\{\mathcal {P}_1,\mathcal {P}_2,\ldots ,\mathcal {P}_n\}$. In fact, $\mathcal {P}$ is just the union $\bigcup _{i=1,2,\ldots , n, \gamma \in P_i}\gamma \mathcal {P}_i$. We expect that the plan $(P_i)_i$ and $\mathcal {P}$ have the same information; we can prove they are MLD.

Now, we replace each component $\mathcal {P}_i$ with an abstract pattern in another abstract pattern space. For example, we construct a uniformly discrete set $\mathcal {R}_i$ that has the same symmetry as $\mathcal {P}_i$. Then, we take the union $\mathcal {S}=\bigcup _{i=1,2,\ldots , n,\gamma \in P_i}\gamma \mathcal {R}_i$. Exactly as above, we expect $\mathcal {S}$ and $(P_i)_i$ to have the same information, and they are MLD for the same reason as above. Since $\mathcal {P}$ and $(P_i)_i$ are MLD, we see that $\mathcal {S}$ is MLD with $\mathcal {P}$. We have constructed a Delone set $\mathcal {S}$.

Here are the problems when doing the same trick in a general context:

1. Above, we take the union of patches and uniformly discrete sets. What is this operation in the abstract context?
2. Can we prove that the plan and the original abstract pattern are MLD, in a general context?
3. How can we construct abstract patterns like $\mathcal {R}_i$ above, for general abstract pattern spaces?

In this section, we first deal with the first problem. In Sect. 4.1 we study an order relation on each abstract pattern space. For example, for ${{\,\mathrm{Patch}\,}}(X)$ and ${{\,\mathrm{UD}\,}}(X)$ we have the relation of inclusion; for $C_c(X)^*$ we have an order relation which means one measure is a restriction of the other. We capture such relations by the framework of abstract pattern spaces. The union is just the supremum of a family of abstract patterns, with respect to this order.

Next, we define glueable abstract pattern spaces in Sect. 4.2. These are abstract pattern spaces in which we can “often” take union of abstract patterns. The definition is weak enough that many interesting examples are glueable; it is strong enough that we can prove interesting properties by using it. For example, by using this gluing operation, we can construct a desirable $\mathcal {S}\in \Sigma$ as above. (We can also prove that, on many subshifts, the local matching metrics are complete [10], by constructing the limit of a Cauchy sequence by gluing operation.)

In Sect. 4.3, we study decomposition of abstract patterns (the second problem in the list). In Proposition 4.28, we prove that the original abstract pattern and the plan are MLD.

In Sect. 4.4, we define family of building blocks, from which abstract patterns are constructed by juxtaposing copies of them. From such a family of building blocks, we can solve the third problem in the list.

In Sect. 4.5, we give a statement of Theorem 4.40, which states that, under a mild condition on the original abstract pattern $\mathcal {P}$ and a set $\Sigma$ of abstract patterns, we can always find $\mathcal {S}\in \Sigma$ that is MLD with $\mathcal {P}$. The proof is given in Appendix A.

### An Order on Abstract Pattern Spaces

Here, we introduce an order relation $\geqq$ on abstract pattern spaces, which captures “inclusion” between abstract patterns in a general context.

#### Definition 4.1

Let $\Pi$ be an abstract pattern space over *X*. We define a relation $\geqq$ on $\Pi$ as follows: for each $\mathcal {P},\mathcal {Q}\in \Pi$, we set $\mathcal {P}\geqq \mathcal {Q}$ if

$$\begin{aligned} \mathcal {P}\wedge {{\,\mathrm{supp}\,}}\mathcal {Q}=\mathcal {Q}. \end{aligned}$$

The following two lemmas are used throughout the article:

#### Lemma 4.2

1. If $$\mathcal {P}\geqq \mathcal {Q}$$, then $${{\,\mathrm{supp}\,}}\mathcal {P}\supset {{\,\mathrm{supp}\,}}\mathcal {Q}$$.
2. The relation $\geqq$ is an order on $\Pi$.

#### Proof

If $$\mathcal {P}\geqq \mathcal {Q}$$, then

$$\begin{aligned} \mathcal {Q}\wedge {{\,\mathrm{supp}\,}}\mathcal {P}=\mathcal {P}\wedge {{\,\mathrm{supp}\,}}\mathcal {P}\wedge {{\,\mathrm{supp}\,}}\mathcal {Q}=\mathcal {P}\wedge {{\,\mathrm{supp}\,}}\mathcal {Q}=\mathcal {Q}. \end{aligned}$$

Thus ${{\,\mathrm{supp}\,}}\mathcal {P}\supset {{\,\mathrm{supp}\,}}\mathcal {Q}$. Next we prove that $\geqq$ is an order. $\mathcal {P}\geqq \mathcal {P}$ is clear. If $\mathcal {P}\geqq \mathcal {Q}$ and $\mathcal {Q}\geqq \mathcal {P}$, then ${{\,\mathrm{supp}\,}}\mathcal {P}={{\,\mathrm{supp}\,}}\mathcal {Q}$ and $\mathcal {P}=\mathcal {P}\wedge {{\,\mathrm{supp}\,}}\mathcal {P}=\mathcal {P}\wedge {{\,\mathrm{supp}\,}}\mathcal {Q}=\mathcal {Q}$. Finally, if $\mathcal {P}\geqq \mathcal {Q}\geqq \mathcal {R}$, then ${{\,\mathrm{supp}\,}}\mathcal {P}\supset {{\,\mathrm{supp}\,}}\mathcal {Q}\supset {{\,\mathrm{supp}\,}}\mathcal {R}$ and $\mathcal {P}\wedge {{\,\mathrm{supp}\,}}\mathcal {R}=\mathcal {P}\wedge {{\,\mathrm{supp}\,}}\mathcal {Q}\wedge {{\,\mathrm{supp}\,}}\mathcal {R}=\mathcal {Q}\wedge {{\,\mathrm{supp}\,}}\mathcal {R}=\mathcal {R}$, so $\mathcal {P}\geqq \mathcal {R}$. $\square$

#### Lemma 4.3

1. If $\mathcal {P}\in \Pi$ and $C\in {{\,\mathrm{Cl}\,}}(X)$, then $\mathcal {P}\geqq \mathcal {P}\wedge C$.
2. If $\mathcal {P},\mathcal {Q}\in \Pi$, $C\in {{\,\mathrm{Cl}\,}}(X)$, and $\mathcal {P}\geqq \mathcal {Q}$, then $\mathcal {P}\wedge C\geqq \mathcal {Q}\wedge C$.

#### Proof

The statements follow from Lemma 2.5.

1. $$\mathcal {P}\wedge {{\,\mathrm{supp}\,}}(\mathcal {P}\wedge C)=\mathcal {P}\wedge {{\,\mathrm{supp}\,}}\mathcal {P}\wedge C\wedge {{\,\mathrm{supp}\,}}(\mathcal {P}\wedge C) =\mathcal {P}\wedge C\wedge {{\,\mathrm{supp}\,}}(\mathcal {P}\wedge C)=\mathcal {P}\wedge C.$$
2. $$\mathcal {P}\wedge C\wedge {{\,\mathrm{supp}\,}}(\mathcal {Q}\wedge C)=\mathcal {P}\wedge {{\,\mathrm{supp}\,}}\mathcal {Q}\wedge C\wedge {{\,\mathrm{supp}\,}}(\mathcal {Q}\wedge C) =\mathcal {Q}\wedge C$$. $$\square $$

The supremum of a subset $\Xi \subset \Pi$ with respect to the order $\geqq$ is the “union” of abstract patterns $\mathcal {P}\in \Xi$. It does not necessarily exist, but is an important concept. Below, we investigate elementary properties of supremum.

#### Definition 4.4

Let $\Xi$ be a subset of $\Pi$. If the supremum of $\Xi$ with respect to the order $\geqq$ defined in Definition 4.1 exists in $\Pi$, it is denoted by $\bigvee \Xi$.

We briefly discuss relations between supremum and support.

#### Lemma 4.5

If a subset $\Xi \subset \Pi$ admits the supremum $\bigvee \Xi$, then ${{\,\mathrm{supp}\,}}\bigvee \Xi =\overline{\bigcup _{\mathcal {P}\in \Xi }{{\,\mathrm{supp}\,}}\mathcal {P}}$.

#### Proof

Set $C=\overline{\bigcup _{\mathcal {P}\in \Xi }{{\,\mathrm{supp}\,}}\mathcal {P}}$. Since $\bigvee \Xi \geqq \mathcal {P}$ for any $\mathcal {P}\in \Xi$, by Lemma 4.3, ${{\,\mathrm{supp}\,}}\bigvee \Xi \supset {{\,\mathrm{supp}\,}}\mathcal {P}$ for each $\mathcal {P}\in \Xi$. Since the support is closed, we have ${{\,\mathrm{supp}\,}}\bigvee \Xi \supset C$.

If we assume that ${{\,\mathrm{supp}\,}}\bigvee \Xi$ is strictly larger than *C*, then we have the following contradiction: Since ${{\,\mathrm{supp}\,}}\bigl (\bigvee \Xi )\wedge C\bigl )\subset C\ne {{\,\mathrm{supp}\,}}\bigvee \Xi$, the two abstract patterns $\bigvee \Xi$ and $\bigl (\bigvee \Xi \bigl )\wedge C$ are different and $\bigvee \Xi \geqq \bigl (\bigvee \Xi \bigl )\wedge C$ by Lemma 4.3. On the other hand, $\bigl (\bigvee \Xi \bigl )\wedge C$ majorizes $\Xi$. These contradict the fact that $\bigvee \Xi$ is the supremum. $\square$

Note that the first part of the proof of Lemma 4.5 shows that, if $\mathcal {Q}$ is an upper bound for $\Xi$, then ${{\,\mathrm{supp}\,}}\mathcal {Q}\supset \overline{\bigcup _{\mathcal {P}\in \Xi }{{\,\mathrm{supp}\,}}\mathcal {P}}$.

The following example may be skipped on a first reading:

#### Example 4.6

It is not necessarily true that any element $\mathcal {P}_{0}$ in $\Pi$ that majorizes $\Xi$ and ${{\,\mathrm{supp}\,}}\mathcal {P}_{0}=\overline{\bigcup _{\mathcal {P}\in \Xi }{{\,\mathrm{supp}\,}}\mathcal {P}}$ is the supremum of $\Xi$. For example, let the abstract pattern space be ${{\,\mathrm{Map}\,}}([0,1],\mathbb {C},0)$ (Example 2.16). Set $\Xi =\{\delta _x\,{|}\, 0<x\leqq 1\}$, where $\delta _x$ is the Kronecker delta function. For each $a\in \mathbb {C}$ define the function $f_a$ via

$$\begin{aligned} f_a(x)={\left\{ \begin{array}{ll} a&{}\text {if } x=0,\\ 1&{}\text {if } x>0. \end{array}\right. } \end{aligned}$$

Then each $f_a$ is an upper bound for $\Xi$ with ${{\,\mathrm{supp}\,}}f_a=[0,1]$ but there is no order relation between $f_a$’s.

### Glueable Abstract Pattern Spaces

*In this subsection*$\Pi$*is an abstract pattern space overX*.

Often we want to “glue” or “take the union of” abstract patterns to obtain a larger abstract pattern. For example, suppose $\Xi$ is a collection of patches such that if $\mathcal {P},\mathcal {Q}\in \Xi$, $S\in \mathcal {P}$, and $T\in \mathcal {Q}$, then we have either $S=T$ or $S\cap T=\emptyset$. Then we can “glue” patches in $\Xi$; that is, we can take the union $\bigcup _{\mathcal {P}\in \Xi }\mathcal {P}$, which is also a patch. Abstract pattern spaces in which we can “glue” abstract patterns are called glueable abstract pattern spaces (Definition 4.11). We define glueable abstract pattern spaces after introducing necessary notions and proving a lemma. Examples are given at the end of this subsection.

#### Definition 4.7

For a set $\Xi$ of abstract patterns over *X* and $C\in {{\,\mathrm{Cl}\,}}(X)$, we set

8 $$\begin{aligned} \Xi \wedge C=\big \{\mathcal {P}\wedge C\,{|}\, \mathcal {P}\in \Xi \big \}. \end{aligned}$$

#### Definition 4.8

1. Two abstract patterns $\mathcal {P},\mathcal {Q}\in \Pi$ are said to be *compatible* if there is $\mathcal {R}\in \Pi$ such that $\mathcal {R}\geqq \mathcal {P}$ and $\mathcal {R}\geqq \mathcal {Q}$.
2. A subset $\Xi \subset \Pi$ is said to be *pairwise compatible* if any two elements $\mathcal {P},\mathcal {Q}\in \Pi$ are compatible.
3. A subset $\Xi \subset \Pi$ is said to be *locally finite* if, for any $x\in X$ and $r>0$, the set $\Xi \wedge B(x,r)$, which was defined in (8), is finite.

#### Remark 4.9

We will prove that, in many abstract pattern spaces, the locally finite and pairwise-compatible subsets admit supremums. Due to Example 4.6, in the space ${{\,\mathrm{Map}\,}}(X,Y,y_0)$ (Example 2.16), a pairwise-compatible $\Xi \subset {{\,\mathrm{Map}\,}}(X,Y,y_0)$ need not have supremum. We have to assume local finiteness for a subset $\Xi$ to admit supremum. Also, in order for a subset of an abstract pattern space to have the supremum, we have to assume pairwise compatibility, since this follows from the existence of supremum.

#### Lemma 4.10

Let $\Xi$ be a subset of $\Pi$ and take $C\in {{\,\mathrm{Cl}\,}}(X)$. Then the following hold:

1. If $\Xi$ is locally finite, then so is $\Xi \wedge C$.
2. If $\Xi$ is pairwise compatible, then so is $\Xi \wedge C$.

#### Proof

1. Suppose there are $x\in X$, $r>0$ such that $\Xi \wedge C\wedge B(x,r)$ is infinite. There are $\mathcal {P}_1,\mathcal {P}_2,\ldots$ in $\Xi$ such that $\mathcal {P}_n\wedge C\wedge B(x,r)$ are all distinct. However, by local finiteness of $\Xi$, there are distinct *n* and *m* such that $\mathcal {P}_n\wedge B(x,r)=\mathcal {P}_m\wedge B(x,r)$; this implies that $\mathcal {P}_n\wedge C\wedge B(x,r)=\mathcal {P}_m\wedge C\wedge B(x,r)$ and leads to a contradiction.

2. Take $\mathcal {P},\mathcal {Q}\in \Xi$ arbitrarily. By Definition 4.8, there is $\mathcal {R}\in \Xi$ such that $\mathcal {R}\geqq \mathcal {P}$ and $\mathcal {R}\geqq \mathcal {Q}$. By Lemma 4.3, we have $\mathcal {R}\wedge C\geqq \mathcal {P}\wedge C$ and $\mathcal {R}\wedge C \geqq \mathcal {Q}\wedge C$, and so $\mathcal {P}\wedge C$ and $\mathcal {Q}\wedge C$ are compatible. $\square$

#### Definition 4.11

An abstract pattern space $\Pi$ over a metric space *X* is said to be *glueable* if the following two conditions hold:

1. If $\Xi \subset \Pi$ is both locally finite and pairwise compatible, then there is the supremum $\bigvee {\Xi }$ for $\Xi$.
2. If $\Xi \subset \Pi$ is both locally finite and pairwise compatible, then for any $C\in {{\,\mathrm{Cl}\,}}(X)$,
  9 $$\begin{aligned} \bigvee (\Xi \wedge C)=\Bigl (\bigvee \Xi \Bigr )\wedge C. \end{aligned}$$

#### Remark 4.12

By Lemma 4.10, for $\Xi \subset \Pi$ which is locally finite and pairwise compatible and $C\in {{\,\mathrm{Cl}\,}}(X)$, the left-hand side of equation (9) makes sense. (The symbol $\Xi \wedge C$ is defined in (8).)

The first condition of this definition does not imply the second. Here is a sketch of the construction of a counterexample: for any abstract pattern space $\Pi$ and an element $\mathcal {P}\in \Pi$, there is the smallest abstract pattern subspace $\Pi (\mathcal {P})$ that contains $\mathcal {P}$. Let $\Pi$ be the abstract pattern space $\Pi ={{\,\mathrm{Pattern}\,}}(\mathbb {R})$ (Example 2.11). Consider the pattern

$$\begin{aligned} \mathcal {P}=\{(0,1)+n\,{|}\, n\in \mathbb {Z}\}\cup \Big \{\Big (\frac{1}{2},\frac{3}{2}\Big )+n \,{\Big |}\, n\in \mathbb {Z}\Big \}. \end{aligned}$$

Any subset $\Xi$ of $\Pi (\mathcal {P})$ admits the supremum, but for $\Xi =\{\{(0,1)\},\{(1,2)\}\}$, we have $\bigvee \Xi =\{(0,1),(1/2,3/2),(1,2)\}$, so $\bigl (\bigvee \Xi \bigr )\wedge [0,3/2]=\{(0,1),(1/2,3/2)\}$ but $\bigvee (\Xi \wedge [0,3/2])=\{(0,1)\}$.

Before listing examples of glueable abstract pattern spaces, we claim that the result of “two-step gluing” is the same as the result of “gluing once.” This will be proved in Appendix A.

#### Lemma 4.13

Let $\Pi$ be glueable and $\Lambda$ a set. For each $\lambda \in \Lambda$, let $\Xi _{\lambda }\subset \Pi$ be a subset and suppose $\bigcup _{\lambda }\Xi _{\lambda }$ is locally finite and pairwise compatible. Then, for each $\lambda$, the set $\Xi _{\lambda }$ is locally finite and pairwise compatible. Moreover, if we set $\mathcal {Q}_{\lambda }=\bigvee \Xi _{\lambda }$, the set $\{\mathcal {Q}_{\lambda }\,{|}\, \lambda \in \Lambda \}$ is locally finite and pairwise compatible and

$$\begin{aligned} \bigvee \bigcup _{\lambda }\Xi _{\lambda }=\bigvee \big \{\mathcal {Q}_{\lambda }\,{|} \,\lambda \in \Lambda \big \}. \end{aligned}$$

We defined the terms “locally finite,” “pairwise compatible,” and “glueable” for abstract pattern spaces over metric spaces in Definitions 4.8 and 4.11. Using these definitions, we define the following concepts:

#### Definition 4.14

Let *X* be a metric space and $\Gamma$ a group which acts on *X* as isometries. Let $\Pi$ be an abstract pattern space over $(X,\Gamma )$. We say $\Pi$ is a *glueable abstract pattern space over*$(X,\Gamma )$ if it is a glueable abstract pattern space over *X*. For a glueable abstract pattern space $\Pi$ over $(X,\Gamma )$, a subset $\Sigma$ of $\Pi$ such that $\bigvee \Xi \in \Sigma$ for any pairwise-compatible and locally finite subset $\Xi$ of $\Sigma$ is said to be *supremum-closed*. (Here, the supremum $\bigvee \Xi$ exists by assumption.)

We have introduced gluing in $\Gamma$-abstract pattern spaces, and it is natural to ask about the relation between the group action and the operation of taking supremum. We can show that $\bigvee$ and the group action are commutative; the proof is given in Appendix A.

#### Lemma 4.15

Let *X* be a metric space and $\Gamma$ a group which acts on *X* as isometries. Let $\Pi$ be a glueable abstract pattern space over $(X,\Gamma )$. If $\gamma \in \Gamma$ and $\Xi \subset \Pi$ is a subset which is both locally finite and pairwise compatible, then $\gamma \Xi =\{\gamma \mathcal {P}\,{|}\,\mathcal {P}\in \Xi \}$ is both locally finite and pairwise compatible. In this case, we have

$$\begin{aligned} \gamma \bigvee \Xi =\bigvee (\gamma \Xi ). \end{aligned}$$

We finish this subsection with examples.

#### Example 4.16

Consider $\Pi ={{\,\mathrm{Patch}\,}}(X)$ (Example 2.9). In this abstract pattern space, for two elements $\mathcal {P},\mathcal {Q}\in {{\,\mathrm{Patch}\,}}(X)$, the following statements hold:

1. $$\mathcal {P}\geqq \mathcal {Q}\iff \mathcal {P}\supset \mathcal {Q}$$.
2. $\mathcal {P}$ and $\mathcal {Q}$ are compatible if and only if, for any $T\in \mathcal {P}$ and $S\in \mathcal {Q}$, either $S=T$ or $S\cap T=\emptyset$ holds.

If $\Xi \subset {{\,\mathrm{Patch}\,}}(X)$ is pairwise compatible, then $\mathcal {P}_{\Xi }=\bigcup _{\mathcal {P}\in \Xi }\mathcal {P}$ is a patch, which is the supremum of $\Xi$. If $C\in {{\,\mathrm{Cl}\,}}(X)$, then

$$\begin{aligned} \Bigl (\bigvee \Xi \Bigr )\wedge C=\biggl (\,\bigcup _{\mathcal {P}\in \Xi }\mathcal {P}\biggr )\wedge C =\bigcup (\mathcal {P}\wedge C)=\bigvee (\Xi \wedge C). \end{aligned}$$

$${{\,\mathrm{Patch}\,}}(X)$$ is glueable.

#### Example 4.17

For the abstract pattern space $2^X$ in Example 2.15, two elements $A,B\in 2^X$ are compatible if and only if

10 $$\begin{aligned} \overline{A}\cap B\subset A\text { and }A\cap \overline{B}\subset B. \end{aligned}$$

Indeed, if *A* and *B* are compatible, then there is a majorant *C*. By $C\supset A\cup B$,

$$\begin{aligned} A\cup (\overline{A}\cap B)=(A\cup B)\cap \overline{A} =C\cap \overline{A}\cap (A\cup B) =A\cap (A\cup B)=A, \end{aligned}$$

and so $\overline{A}\cap B\subset A$. A similar argument shows that $\overline{B}\cap A\subset B$. Conversely, if condition (10) holds, then $(A\cup B)\cap \overline{A}=A\cup (B\cap \overline{A})=A$ and similarly $(A\cup B)\cap \overline{B}=B$, and so $A\cup B$ is a majorant for *A* and *B*.

Suppose that $\Xi \subset 2^X$ is locally finite and pairwise compatible. Note that $\bigcup _{A\in \Xi }\overline{A}=\overline{\bigcup _{A\in \Xi }A}$. Set $A_{\Xi }=\bigcup _{A\in \Xi }A$. For each $A\in \Xi$, $A_{\Xi }\cap \overline{A}=\bigcup _{B\in \Xi }(B\cap \overline{A})=A$; $A_{\Xi }$ is a majorant of $\Xi$. If *B* is also a majorant for $\Xi$, then

$$\begin{aligned} B\cap \overline{A_{\Xi }}=B\cap \biggl (\bigcup _{A\in \Xi }\overline{A}\biggr ) =\bigcup _{A\in \Xi }(B\cap \overline{A}) =\bigcup _{A\in \Xi }A=A_{\Xi }, \end{aligned}$$

and so $B\geqq A_{\Xi }$. It turns out that $A_{\Xi }$ is the supremum for $\Xi$. Moreover, if $C\in {{\,\mathrm{Cl}\,}}(X)$, then $A_{\Xi }\wedge C=\bigcup _{A\in \Xi }(A\cap C)=\bigvee (\Xi \wedge C)$. Thus $2^X$ is a glueable space.

#### Remark 4.18

Let $\Pi _0$ be a glueable abstract pattern space and $\Pi _1\subset \Pi _0$ an abstract pattern subspace. For any subset $\Xi \subset \Pi _1$, if it is pairwise compatible in $\Pi _1$, then it is pairwise compatible in $\Pi _0$. Moreover, whether a set is locally finite or not is independent of the ambient abstract pattern space in which the set is included.

For a subset $\Xi \subset \Pi _1$ which is locally finite and pairwise compatible in $\Pi _1$, since $\Pi _0$ is glueable, there is the supremum $\bigvee \Xi$ in $\Pi _0$. If this supremum in $\Pi _0$ is always included in $\Pi _1$, then $\Pi _1$ is glueable.

By this remark, it is easy to see that the abstract pattern spaces ${{\,\mathrm{Cl}\,}}(X)$ (Example 2.15), ${{\,\mathrm{LF}\,}}(X)$ (Example 2.13), and ${{\,\mathrm{UD}\,}}_r(X)$ (Example 2.14, *r* is an arbitrary positive number) are glueable.

However, ${{\,\mathrm{UD}\,}}(X)$ (Example 2.14) is not necessarily glueable. For example, set $X=\mathbb {R}$. Set $\mathcal {P}_n=\bigl \{ n, n + \frac{1}{n} \bigr \}$ for each integer $n\ne 0$. Each $\mathcal {P}_n$ is in ${{\,\mathrm{UD}\,}}(\mathbb {R})$; $\Xi =\{\mathcal {P}_n\,{|}\, n\ne 0\}$ is locally finite and pairwise compatible, but it does not admit the supremum.

The proof of the following proposition is given in Appendix A:

#### Proposition 4.19

Let *X* be a metric space, *Y* a set, and $y_0\in Y$. Then ${{\,\mathrm{Map}\,}}(X,Y,y_0)$ is glueable.

### Decomposition of Abstract Patterns by Delone Sets

#### Setting 4

For the rest of this section, $X={\mathbb {R}}^d$ and $\Gamma$ is a closed subgroup of  $\mathrm{E}(d)$ that contains ${\mathbb {R}}^d$. $\Pi$, $\Pi _1$, and $\Pi _2$ are glueable abstract pattern spaces over $({\mathbb {R}}^d,\Gamma )$.

Note that we endow $\Gamma$ with a metric $\rho _{\Gamma }$ given in Notation 1.1.

To explain the idea of this subsection, consider a tiling $\mathcal {T}$ in ${\mathbb {R}}^d$, where we only consider translations. Assume that the diameters of tiles are bounded from above. Suppose we pick one point $x_T$ from each $T\in \mathcal {T}$ in such a way that, if $S,T\in \mathcal {T}$ are translationally equivalent, then $x_T$ and $x_S$ are also translationally equivalent by the same vector. Then the set $D=\{x_T\,{|}\, T\in \mathcal {T}\}$ is a Delone set that is locally derivable from $\mathcal {T}$. Since the diameters of tiles in $\mathcal {T}$ are bounded, for $R>0$ large enough, we have

$$\begin{aligned} \mathcal {T}=\bigcup _{x\in D}\mathcal {P}\wedge B(x,R)=\bigvee \big \{\mathcal {P}\wedge B(x,R)\,{|}\, x\in D\big \}. \end{aligned}$$

In this way we can “decompose” $\mathcal {T}$ into a family of patches $\Xi =\{\mathcal {P}\wedge B(x,R)\,{|}\, x\in D\}$, from which we can reconstruct $\mathcal {T}$. Each element of $\Xi$ describes the behavior of $\mathcal {T}$ around *x*, and we can take a tuple $(\mathcal {P}_{\lambda })_{\lambda }$ of patches which are located around $0\in {\mathbb {R}}^d$ and such that, for each $x\in D$, there is one and only one $\mathcal {P}_{\lambda }$ such that $\mathcal {T}\wedge B(x,R)=\mathcal {P}_{\lambda }+x$. In other words, $(\mathcal {P}_{\lambda })_{\lambda }$ is the tuple of all possible behaviors of $\mathcal {T}$ around each $x\in D$. We can reconstruct $\mathcal {T}$ from “the tuple of components,” $(\mathcal {P}_{\lambda })_{\lambda }$, and the plan, that is, the information of “where translates of each $\mathcal {P}_{\lambda }$ appears,” just as a machine or building is constructed from its components and plans. We show that the original $\mathcal {T}$ and its plan are MLD (Proposition 4.28).

#### Definition 4.20

Take an abstract pattern $\mathcal {P}\in \Pi$. We say a pair (*D*, *R*) of a Delone set in *X* and a positive number $R>0$*decomposes*$\mathcal {P}$ if the following three conditions are satisfied:

1. $$\mathcal {P}\overset{{\mathrm {LD}}}{\rightarrow }D$$,
2. $$\mathcal {P}=\bigvee \{\mathcal {P}\wedge B(x,R)\,{|}\, x\in D\}$$, and
3. $$\sup _{x\in D}{{\,\mathrm{card}\,}}({{\,\mathrm{Sym}\,}}_{\Gamma _x}\mathcal {P}\wedge B(x,R))$$ is finite.

In this definition, the third condition is a technical one that only arises when we consider $\mathrm{O}(d)$-actions. We first investigate a relation between decomposition by a Delone set and a positive number, and the group action $\Gamma \curvearrowright \Pi$.

#### Lemma 4.21

If $(D,R_0)$ decomposes $\mathcal {P}$ and $\gamma \in \Gamma$, then $(\gamma D,R_0)$ decomposes $\gamma \mathcal {P}$.

*For the rest of this subsection*$\mathcal {P}$*is an element of*$\Pi$, *Da Delone set inX*, *and*$R_0$*a positive real number, and we assume that*$(D,R_0)$*decomposes*$\mathcal {P}$. We will use the following lemma to define tuple of components and plan:

#### Lemma 4.22

There exist a set $\Lambda$ and $\mathcal {P}_{\lambda }\in \Pi$ for each $\lambda \in \Lambda$ such that

1. for each $\lambda \in \Lambda$, we have ${{\,\mathrm{supp}\,}}\mathcal {P}_{\lambda }\subset B(0,R_0)$, and
2. for each $x\in D$ there are a unique $\lambda _x\in \Lambda$ and $\gamma \in \Gamma$ such that $\mathcal {P}\wedge B(x,R_0)=\gamma \mathcal {P}_{\lambda _x}$ and $x=\gamma 0$.

#### Proof

Define an equivalence relation $\sim$ on *D* as follows: we have $x\sim y$ if there is $\gamma \in \Gamma$ such that (1) $\gamma x=y$, and (2) $\gamma (\mathcal {P}\wedge B(x,R_0))=\mathcal {P}\wedge B(y,R_0)$. Then by taking one point from each equivalence class for $\sim$, we obtain a set $\Lambda$.

For each $x\in \Lambda$, take an element $\gamma _x\in \Gamma$ such that $\gamma _x 0=x$. Set $\mathcal {P}_x=\gamma _x^{-1}(\mathcal {P}\wedge B(x,R_0))$; then $\Lambda$ and $\mathcal {P}_x,x\in \Lambda$ satisfy the conditions. $\square$

#### Remark 4.23

By the second condition of Lemma 4.22, we see that ${{\,\mathrm{Sym}\,}}_{\Gamma _{0}}\mathcal {P}_{\lambda _x}$ is conjugate to ${{\,\mathrm{Sym}\,}}_{\Gamma _x}\mathcal {P}\wedge B(x,R_0)$. In particular, ${{\,\mathrm{card}\,}}{{\,\mathrm{Sym}\,}}_{\Gamma _{0}}\mathcal {P}_{\lambda }$, where $\lambda \in \Lambda$, is bounded from above.

#### Definition 4.24

Any tuple of abstract patterns $(\mathcal {P}_{\lambda })_{\lambda \in \Lambda }$ which satisfies the conditions in Lemma 4.22 is called a *tuple of components* for $\mathcal {P}$ with respect to $(D,R_0)$. For each $\lambda \in \Lambda$, set

$$\begin{aligned} P_{\lambda }= P_{\lambda }(\mathcal {P},D,R_0,(\mathcal {P}_{\lambda })_{\lambda }) =\bigl \{\gamma \in \Gamma \mid \gamma 0\in D\text { and }\mathcal {P}\wedge B(\gamma 0,R_0)=\gamma \mathcal {P}_{\lambda }\bigr \} \end{aligned}$$

and call the tuple $(P_{\lambda })_{\lambda }$ the *plan* for $\mathcal {P}$ with respect to $(D,R_0,(\mathcal {P}_{\lambda }))$.

#### Example 4.25

Define two labeled tiles $I_W$ and $I_B$ in ${\mathbb {R}}^d$ via $I_W=([0,1]^d,W)$ and $I_B=([0,1]^d,B)$. These are “black tile” and “white tile,” and we can consider a (labeled) tiling $\mathcal {T}$ in ${\mathbb {R}}^d$ like a checkerboard, that is, the set of all tiles $I_B+(z_1,z_2,\ldots ,z_d)$ with $z_j\in \mathbb {Z}$ and $z_1+z_2+\cdots +z_d\in 2\mathbb {Z}$ and $I_W+(z_1,z_2,\ldots ,z_d)$ with $z_j\in \mathbb {Z}$ and $z_1+z_2+\cdots +z_d\in 2\mathbb {Z}+1$. A Delone set $\mathbb {Z}^d$ is locally derivable from this tiling $\mathcal {T}$. For any large $R>0$, the tuple of components is two patches $\mathcal {P}_{R,B}$ and $\mathcal {P}_{R,W}$, where the former is nothing but $\mathcal {T}\wedge B(0,R)$ and the latter is obtained by reversing colors of tiles in the former. The plan for this tuple of components is $\bigl \{(z_1,z_2,\ldots ,z_d)\in \mathbb {Z}^d\,{|}\, \sum z_j\in 2\mathbb {Z}\bigr \}$ and $\bigl \{(z_1,z_2,\ldots ,z_d)\in \mathbb {Z}^d\,{|}\, \sum z_j\in 2\mathbb {Z}+1\bigr \}$. (Here we only consider translations, but if $\Gamma$ is larger than ${\mathbb {R}}^d$, the plan becomes bigger.)

The following lemma on a relation among the group action, tuple of components, and plan is easy to prove:

#### Lemma 4.26

Let $(\mathcal {P}_{\lambda })_{\lambda \in \Lambda }$ be a tuple of components for $\mathcal {P}$ with respect to $(D,R_0)$. Let $(P_{\lambda })_{\lambda \in \Lambda }$ be the plan for $\mathcal {P}$ with respect to $(D,R_0,(\mathcal {P}_{\lambda }))_{\lambda }$. For any $\gamma \in \Gamma$, $(\mathcal {P}_{\lambda })_{\lambda }$ is a tuple of components for $\gamma \mathcal {P}$ with respect to $(D,R_0)$ and $(\gamma P_{\lambda })_{\lambda }$ is the plan for $\gamma \mathcal {P}$ with respect to $(D,R_0,(\mathcal {P}_{\lambda }))_{\lambda }$.

#### Remark 4.27

Let $(\mathcal {P}_{\lambda })_{\lambda \in \Lambda }$ be a tuple of components for $\mathcal {P}$ with respect to $(D,R_0)$. Let $(P_{\lambda })$ be the plan for $\mathcal {P}$ with respect to $(D,R_0,(\mathcal {P}_{\lambda }))$. Then

$$\begin{aligned} \big \{\mathcal {P}\wedge B(x,R_0)\,{|}\, x\in D\big \}=\big \{\gamma \mathcal {P}_{\lambda }\,{|}\,\lambda \in \Lambda , \gamma \in P_{\lambda }\big \}. \end{aligned}$$

This implies that $\mathcal {P}=\bigvee \big \{\gamma \mathcal {P}_{\lambda }\,{|}\,\lambda \in \Lambda , \gamma \in P_{\lambda }\big \}$.

Now we claim the goal of this subsection. The proof is given in Appendix A.

#### Proposition 4.28

Let $(\mathcal {P}_{\lambda })_{\lambda \in \Lambda }$ be a tuple of components for $\mathcal {P}$ with respect to $(D,R_0)$ and $(P_{\lambda })$ the plan for $\mathcal {P}$ with respect to $(D,R_0,(\mathcal {P}_{\lambda }))$. If we regard $(P_{\lambda })$ as an abstract pattern of $\prod _{\lambda \in \Lambda }2^{\Gamma }$, which is an abstract pattern space over $(\Gamma ,\Gamma )$, (Lemma 2.27, Definition 2.28, Example 2.30), we have

$$\begin{aligned} \mathcal {P}\overset{{\mathrm {MLD}}}{\leftrightarrow }(P_{\lambda })_{\lambda }. \end{aligned}$$

#### Remark 4.29

For tilings and Delone sets we have the concept of finite local complexity (FLC). We can generalize this concept to many abstract patterns. If $\mathcal {P}$ has FLC, then the index set $\Lambda$ in Definition 4.24 is finite.

### Families of Building Blocks and Admissible Digits

In the previous subsection, we studied the decomposition of abstract patterns. Here, we study construction of abstract patterns from “building blocks.”

#### Setting 5

In this subsection we assume, in addition to Setting 4, that $\Sigma$ is a supremum-closed subshift inside $\Pi$.

Here we define and study “building blocks” and “admissible digits.” For example, for any $r>0$, consider a continuous positive function $\varphi$ on ${\mathbb {R}}^d$ the support of which is included in *B*(0, *r*). This is a building block for the set of all continuous functions, which is a subshift inside ${{\,\mathrm{Map}\,}}({\mathbb {R}}^d,\mathbb {C},0)$ (Example 2.31). We call this a building block, since if we take two $\gamma ,\eta \in \Gamma$ such that $\rho (\gamma 0,\eta 0)>4r$, we can juxtapose $\gamma \varphi$ and $\eta \varphi$ to obtain a new continuous map, that is, the map *f* such that

$$\begin{aligned} f(x)={\left\{ \begin{array}{ll} \gamma \varphi (x)&{}\text { if }x\in {{\,\mathrm{supp}\,}}\gamma \varphi ,\\ \eta \varphi (x)&{}\text { if }x\in {{\,\mathrm{supp}\,}}\eta \varphi ,\\ 0&{}\text { otherwise}. \end{array}\right. } \end{aligned}$$

Since the space of all continuous functions is a supremum-closed subshift inside a glueable abstract pattern space ${{\,\mathrm{Map}\,}}({\mathbb {R}}^d,\mathbb {C},0)$, in order to obtain a new function like *f* above, all we need is the compatibility of $\gamma \varphi$ and $\eta \varphi$. In general, a building block is an abstract pattern in a subshift that always satisfies such a compatibility condition. We sometimes require the symmetry group of the building block to be $\Gamma _0$; in this case the building block is said to be symmetric.

Given such a building block $\mathcal {P}$, a subset $\mathcal {D}$ of $\Gamma$ is called an admissible digit if $\gamma ,\eta \in \mathcal {D}$ and $\rho (\gamma 0,\eta 0)\leqq 4r$, then $\gamma \mathcal {P}=\eta \mathcal {P}$. If $\mathcal {D}$ is such a set, then $\{\gamma \mathcal {P}\,{|}\,\gamma \in \mathcal {D}\}$ is locally finite and pairwise compatible, and so we can take a supremum inside the subshift. This operation of taking supremum is used in the construction of $\mathcal {R}_{\lambda }$ that was explained at the beginning of this section.

#### Definition 4.30

Take a positive real number $r>0$ arbitrarily. An element $\mathcal {P}\in \Sigma$ is called a *symmetric building block forr* if the following conditions are satisfied:

1. $${{\,\mathrm{supp}\,}}\mathcal {P}\subset B(0,r)$$.
2. If $\gamma ,\eta \in \Gamma$ and $\rho (\gamma 0,\eta 0)>4r$, then $\gamma \mathcal {P}$ and $\eta \mathcal {P}$ are compatible.
3. $${{\,\mathrm{Sym}\,}}_{\Gamma }\mathcal {P}=\Gamma _0$$.

#### Example 4.31

If $\Sigma$ is the set of all *r*-uniformly discrete sets in ${\mathbb {R}}^d$, then the one-point set $\{0\}$ is a symmetric building block for any $r>0$.

#### Example 4.32

If $\Sigma$ is the set of all continuous functions inside ${{\,\mathrm{Map}\,}}({\mathbb {R}}^d,\mathbb {C},0)$ (Example 2.31), the bump function $\varphi$, which is defined via

$$\begin{aligned} \varphi (x)={\left\{ \begin{array}{ll} \mathrm {e}^{\frac{1}{\Vert x\Vert -r}}&{} \text { if }\Vert x\Vert <r,\\ 0 &{} \text { otherwise}, \end{array}\right. } \end{aligned}$$

is a symmetric building block for *r*.

To construct $\mathcal {R}_{i}$ at the beginning of this section, we first construct $\mathcal {E}$, see Step 1: construction of $\mathcal {E}$, in Appendix A (Fig. 1), which has trivial symmetry, by juxtaposing a symmetric building block $\mathcal {P}_r$. We then juxtapose $\mathcal {P}_r$ and $\mathcal {E}$ to construct $\mathcal {R}_{i}$, whose symmetry group is the same as that of $\mathcal {P}_{i}$. Figures 2 and 3 show $\mathcal {R}_{i}$ for the case where the symmetry group is the cyclic group or dihedral group and the dimension *d* of the Euclidean space is 2. Each black dot denotes a copy of the symmetric building block.

**Fig. 1:**
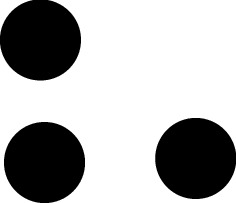
Image of ${\mathcal {E}}$. Each dot represents a copy of a symmetric building block ${\mathcal {P}}_r$

**Fig. 2:**
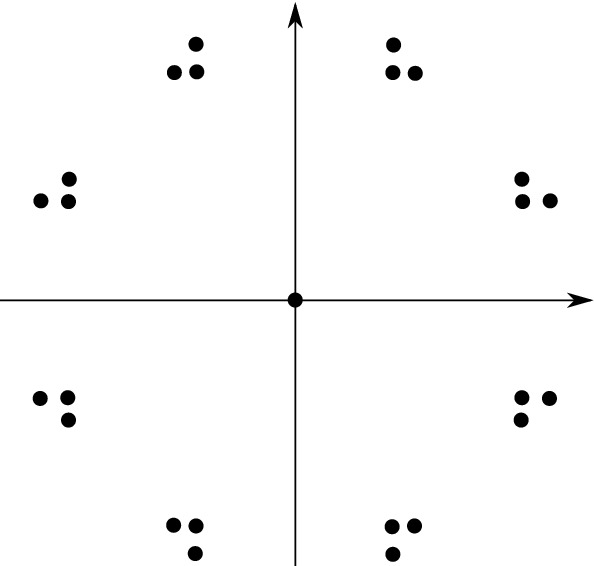
$\mathcal {R}_{i}$ if the symmetry group is $D_4$

**Fig. 3:**
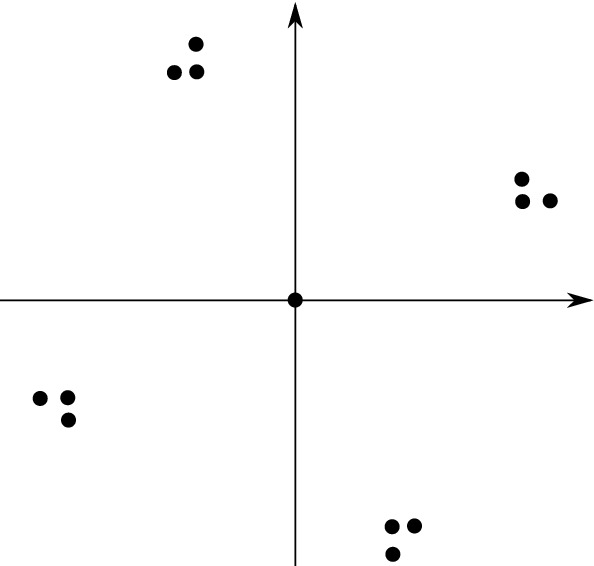
$\mathcal {R}_{i}$ if the symmetry group is $C_4$

The rest of this subsection will be used only in Appendix A, and so may be skipped on a first reading. We introduce the notion of family of building blocks. Each element of a family of building blocks is a building block.

#### Definition 4.33

Take a positive number $r>0$ arbitrarily. A subset $\mathfrak {F}\subset \Sigma$ is called a *family of building blocks of*$\Sigma$*forr* if the following three conditions are satisfied:

1. $\mathfrak {F}\ne \emptyset$ and $\emptyset \ne {{\,\mathrm{supp}\,}}\mathcal {P}\subset B(0,r)$ for each $\mathcal {P}\in \mathfrak {F}$.
2. If $\gamma ,\eta \in \Gamma$, $\mathcal {P},\mathcal {Q}\in \mathfrak {F}$, and $\rho (\gamma 0,\eta 0)>4r$, then $\gamma \mathcal {P}$ and $\eta \mathcal {Q}$ are compatible.
3. If $\mathcal {P},\mathcal {Q}\in \mathfrak {F}$, $\gamma \in \Gamma$, and $\gamma \mathcal {P}=\mathcal {Q}$, then $\mathcal {P}=\mathcal {Q}$ and $\gamma 0=0$.

The elements of $\mathfrak {F}$ are called *building blocks* for *r*. If a building block $\mathcal {P}$ for *r* additionally satisfies the condition

$$\begin{aligned} {{\,\mathrm{Sym}\,}}_{\Gamma }\mathcal {P}=\Gamma _{0}, \end{aligned}$$

then $\mathcal {P}$ is a symmetric building block for *r*.

Let $\mathfrak {F}$ be a family of building blocks of $\Sigma$ for *r*. Then, a tuple $(\mathcal {D}_{\mathcal {P}})_{\mathcal {P}\in \mathfrak {F}}$ of subsets $\mathcal {D}_{\mathcal {P}}\subset \Gamma$ is called an *admissible digit* if

$$\begin{aligned} \mathcal {P},\mathcal {Q}\in \mathfrak {F}, \quad \gamma \in \mathcal {D}_{\mathcal {P}}, \quad \eta \in \mathcal {D}_{\mathcal {Q}} \quad \text {and} \quad \rho (\gamma 0,\eta 0)\leqq 4r \end{aligned}$$

imply $$\mathcal {P}=\mathcal {Q}$$ and $$\gamma \mathcal {P}=\eta \mathcal {Q}$$.

#### Remark 4.34

A nonempty subset of a family of building blocks is again a family of building blocks.

We now give examples, then prove a lemma that will be useful later.

#### Example 4.35

Consider the abstract pattern space ${{\,\mathrm{Patch}\,}}({\mathbb {R}}^d)$ over $({\mathbb {R}}^d,{\mathbb {R}}^d)$ (Examples 2.9, 2.29). The set $\{(0,1)^d,(0,2)^d\}$ is an example of a family of building blocks for $2\sqrt{d}$, since if any element of one patch and any element of another patch are disjoint, those patches are compatible (Example 4.16).

#### Example 4.36

Consider the abstract pattern space ${{\,\mathrm{UD}\,}}_r({\mathbb {R}}^d)$ over $({\mathbb {R}}^d,\Gamma )$ (Example 2.30), where $\Gamma$ is as in Setting 4. The one-point set $\{0\}$ is a symmetric building block for *r*, as described above. For example, $(\mathcal {D}_{\{0\}})$, where $\mathcal {D}_{\{0\}}=5r\mathbb {Z}^d$, is an admissible digit.

#### Lemma 4.37

Let $\mathfrak {F}$ be a family of building blocks for *r* and $(\mathcal {D}_{\mathcal {P}})_{\mathcal {P}\in \mathfrak {F}}$ be an admissible digit. Then $\{\gamma \mathcal {P}\,{|}\,\mathcal {P}\in \mathfrak {F}, \gamma \in \mathcal {D}_{\mathcal {P}}\}$ is locally finite and pairwise compatible.

#### Proof

Clear by definition. $\square$

Since a family of building blocks is inside a supremum-closed subshift, there is a supremum $\bigvee \{\gamma \mathcal {P}\,{|}\,\mathcal {P}\in \mathfrak {F}, \gamma \in \mathcal {D}_{\mathcal {P}}\}$ inside $\Sigma$ under the same condition as in Lemma 4.37.

### The Translation Theorem

Here we state Theorem 4.40, which is the goal of this section. We use the following notion in order to state the theorem:

#### Definition 4.38

Let $\Pi$ be an abstract pattern space over $(X,\Gamma )$. $\mathcal {P}\in \Pi$ is said to be *Delone-deriving* if there is a Delone set *D* in *X* such that $\mathcal {P}\overset{{\mathrm {LD}}}{\rightarrow }D$.

#### Remark 4.39

Delone sets are Delone-deriving. If a tiling consists of finitely many types of tiles up to $\Gamma$ and each tile *T* admits a fixed point of its symmetry group ${{\,\mathrm{Sym}\,}}_{\Gamma }T$, then the tiling is Delone-deriving. On the other hand, constant functions are not Delone-deriving. Note that the symmetry group of a Delone set is discrete whereas the symmetry group of a constant function is not. If an abstract pattern is Delone-deriving, then it is “discrete” in a sense.

It is worth noting that, if $\mu \in C_c(X)^*$ and $|\mu |$ is its total variation, we have $\mu \overset{{\mathrm {LD}}}{\rightarrow }|\mu |\overset{{\mathrm {LD}}}{\rightarrow }{{\,\mathrm{supp}\,}}|\mu |$, where ${{\,\mathrm{supp}\,}}|\mu |$ coincides with the usual support of the positive measure $|\mu |$. In particular, if $\mu =\sum _{x\in D}w(x)\delta (x)$ for some function *w* and a Delone $D\subset X$, then $\mu \overset{{\mathrm {LD}}}{\rightarrow }D$, and $\mu$ is Delone-deriving.

#### Theorem 4.40

In addition to Setting 4, assume $\Sigma$ is a supremum-closed subshift of $\Pi _2$ that contains sufficiently many symmetric building blocks, which means that, for each $r>0$, there is a symmetric building block $\mathcal {P}_r$ for *r* (Definition 4.33). Let $\mathcal {P}$ be an abstract pattern in $\Pi _1$ which consists of bounded components (Definition 2.24) and is Delone-deriving. Then there is an abstract pattern $\mathcal {S}$ in $\Sigma$ such that $\mathcal {P}\overset{{\mathrm {MLD}}}{\leftrightarrow }\mathcal {S}$. Moreover, ${{\,\mathrm{supp}\,}}\mathcal {S}$ is relatively dense in ${\mathbb {R}}^d$.

#### Remark 4.41

This theorem holds if we replace $({\mathbb {R}}^d,\Gamma )$ with a pair $(X,\Gamma )$ of a proper metric space *X* and a group $\Gamma$ that acts on *X* transitively as isometries such that inequality (1) and Lemmas A.14, A.18, and A.19 hold if we replace 2 on the right-hand side of (1) with some positive number and $0\in {\mathbb {R}}^d$ in these assertions with some point in *X*.

By the same proof, we can prove a slightly stronger statement:

#### Theorem 4.42

In addition to Setting 4, assume $\Sigma$ is a subset of $\Pi _2$ such that, for any $r>0$, there is a supremum-closed subshift $\Sigma _r$ of $\Pi _2$ that is included in $\Sigma$ and that contains a symmetric building block $\mathcal {P}_r$ for *r*. Let $\mathcal {P}$ be an abstract pattern in $\Pi _1$ which consists of bounded components (Definition 2.24) and is Delone-deriving. Then there is an abstract pattern $\mathcal {S}$ in $\Sigma$ such that $\mathcal {P}\overset{{\mathrm {MLD}}}{\leftrightarrow }\mathcal {S}$. Moreover, ${{\,\mathrm{supp}\,}}\mathcal {S}$ is relatively dense in ${\mathbb {R}}^d$.

#### Corollary 4.43

Under the same assumption as in Theorem 4.42 on $\mathcal {P}$, there is a Delone set *D* in ${\mathbb {R}}^d$ that is MLD with $\mathcal {P}$.

#### Proof

If $\Sigma ={{\,\mathrm{UD}\,}}({\mathbb {R}}^d)$, for each $r>0$, $\Sigma _r={{\,\mathrm{UD}\,}}_r(X)$ is supremum-closed and the one-point set $\mathcal {P}=\{0\}$ is a symmetric building block for *r*. This $\Sigma$ satisfies the condition in Theorem 4.42.$\square$

Note that, if $\Gamma$ is bigger than ${\mathbb {R}}^d$, our “MLD” means “S-MLD” in [4].

#### Remark 4.44

In Sect. 5, we give sufficient conditions for a subshift of functions to have sufficiently many symmetric building blocks. We will be able to apply Theorem 4.40 when $\Sigma$ is a space of certain functions under a mild condition.

## A Study of Abstract Patterns via Arrows II: the Cases with $\mathrm{O}(d)$-Actions

Rand [11] generalized the definition of pattern-equivariant functions in another way to incorporate rotations and flips in the two-dimensional cases. Recall the notation $\mathcal {T}\sqcap C$ for a tiling $\mathcal {T}$ and a closed $C\subset {\mathbb {R}}^d$ defined in Notation 2.12.

### Definition 5.1

[11] Let $\mathcal {T}$ be a tiling of ${\mathbb {R}}^d$, $\Gamma$ a closed subgroup of $\mathrm{E}(d)$ that contains ${\mathbb {R}}^d$, *G* an Abelian group, and $\phi :\Gamma _0\rightarrow {{\,\mathrm{Aut}\,}}(G)$ a group homomorphism. Here, ${{\,\mathrm{Aut}\,}}(G)$ is the group of automorphisms of *G*. We say a function $f:{\mathbb {R}}^d\rightarrow G$ is $\mathcal {T}$*-equivariant* with representation $\phi$, or is $\phi$-invariant, if there is $R>0$ such that $x,x'\in {\mathbb {R}}^d$, $\gamma \in \Gamma _0$, and

$$\begin{aligned} (\mathcal {T}\sqcap B(x',R))-x'=\gamma (\mathcal {T}\sqcap B(x,R)-x) \end{aligned}$$

imply $$f(x')=\phi (\gamma )(f(x))$$.

In this case, we can also capture pattern-equivariant functions in terms of local derivability. For what follows, let $\pi :\Gamma \ni (a,A)\mapsto A\in \Gamma _0$ be the projection.

### Lemma 5.2

Let $\mathcal {T}$ be a tiling which consists of bounded components. Let $\Gamma$ be a closed subgroup of $\mathrm{E}{(d)}$ that contains ${\mathbb {R}}^d$, *G* an Abelian group, and $\phi :\Gamma _0\rightarrow {{\,\mathrm{Aut}\,}}(G)$ a group homomorphism. Then, for any $f\in {{\,\mathrm{Map}\,}}({\mathbb {R}}^d,G)$, *f* is $\mathcal {T}$-equivariant with representation $\phi$ if and only if $\mathcal {T}\overset{{\mathrm {LD}}}{\rightarrow }f$. Here, $\mathcal {T}$ is regarded as an element of ${{\,\mathrm{Patch}\,}}({\mathbb {R}}^d)$ (Example 2.29), which is an abstract pattern space over $({\mathbb {R}}^d,\Gamma )$, and *f* is regarded as an element of ${{\,\mathrm{Map}\,}}_{\phi \circ \pi }({\mathbb {R}}^d,G,e)$ (Example 2.31), which is an abstract pattern space over $({\mathbb {R}}^d,\Gamma )$.

For Rand’s definition, it may be that there is no maximal pattern-equivariant functions, but Theorem 4.40 gives us a sufficient condition for $\mathcal {P}$ and $\Sigma$ to admit maximal elements. Thus we obtain a result (Theorem 5.5) similar to Theorem 3.6, which says that the space of pattern-equivariant functions has all the information on the original object up to MLD. Here is the setting for the rest of this section: *let*$\Gamma$*be a closed subgroup of*$\mathrm{E}(d)$*that contains*${\mathbb {R}}^d$. *Take a group homomorphism*$\phi :\Gamma _0\rightarrow GL_m(\mathbb {C})$. *Let*$C^{\infty }_{\phi \circ \pi }({\mathbb {R}}^d,\mathbb {C}^m,0)$*be the subshift of*${{\,\mathrm{Map}\,}}_{\phi \circ \pi }({\mathbb {R}}^d,\mathbb {C}^m,0)$*consisting of all smooth elements of*${{\,\mathrm{Map}\,}}_{\phi \circ \pi }({\mathbb {R}}^d,\mathbb {C}^m,0)$. (*We say a map*$f:{\mathbb {R}}^d\rightarrow \mathbb {C}^m$*is smooth if*$\langle f(\cdot ),v\rangle$*is smooth for any*$v\in \mathbb {C}^m$, *where*$\langle \cdot ,\cdot \rangle$*is the standard inner product.*) In order to apply Lemma 3.2 to $\Sigma =C^{\infty }_{\phi \circ \pi }({\mathbb {R}}^d,G,0)$, we need to show that $\Sigma$ admits sufficiently many symmetric building blocks. We show in two cases that there are sufficiently many building blocks (Lemmas 5.3 and 5.4).

### Lemma 5.3

Suppose there is $v\in \mathbb {C}^m\setminus \{0\}$ such that $\phi (\gamma ) v=v$ for each $\gamma \in \Gamma _0$. Then $C^{\infty }_{\phi \circ \pi }({\mathbb {R}}^d,\mathbb {C}^m,0)$ has sufficiently many symmetric building blocks: in other words, for any $r>0$, there is a symmetric building block $g_r$ for (0, *r*).

### Proof

For each $r>0$, set

$$\begin{aligned} f_r(x)={\left\{ \begin{array}{ll} \exp \bigl (-\frac{1}{r^2-\Vert x\Vert ^2}\bigr ) &{}\text {if }\Vert x\Vert <r,\\ 0 &{}\text {otherwise} \end{array}\right. } \end{aligned}$$

for each $x\in {\mathbb {R}}^d$. Then $f_r$ is a smooth real-valued function on ${\mathbb {R}}^d$. Set $g_r(x)=f_r(x)v$. Then $\emptyset \ne {{\,\mathrm{supp}\,}}g_r\subset B(0,r)$. Moreover, if $\gamma ,\eta \in \Gamma$ and $\rho (\gamma 0,\eta 0)>4r$, then $\gamma g_r$ and $\eta g_r$ are compatible since

$$\begin{aligned} g(x)= {\left\{ \begin{array}{ll} \gamma g_r(x)&{}\text {if }x\in B(\gamma 0,r),\\ \eta g_r(x) &{}\text {if }x\in B(\eta 0,r),\\ 0 &{}\text {otherwise} \end{array}\right. } \end{aligned}$$

is a majorant. Finally ${{\,\mathrm{Sym}\,}}_{\Gamma }g_r=\Gamma _0$. $\square$

### Lemma 5.4

Suppose $\Gamma _0$ is finite. Then $C^{\infty }_{\phi \circ \pi }({\mathbb {R}}^d,\mathbb {C}^m,0)$ has sufficiently many building blocks.

### Proof

For any $r>0$, take $x\in {\mathbb {R}}^d$ and $r_1\in (0,r/4)$ such that $\Vert x\Vert <r/2$ and, if $A\in \Gamma _0$ and $A\ne I$, then $\Vert Ax-x\Vert >4r_1$. Take $v\in \mathbb {C}^m$ and set $f(x)=f_{r_1}(x)v$ (we defined $f_{r_1}$ in the proof of Lemma 5.3). Set $h=\bigvee \{(A,Ax)f\,{|}\, A\in \Gamma _0\}$. Then *h* is a symmetric building block. $\square$

### Theorem 5.5

Assume the same assumption as in Lemma 5.3 or in Lemma 5.4. Let $\Pi$ and $\Pi '$ be glueable abstract pattern spaces over $({\mathbb {R}}^d,\Gamma )$ and take $\mathcal {P}$ and $\mathcal {P}'$ from $\Pi$ and $\Pi '$, respectively. Assume that $\mathcal {P}$ and $\mathcal {P}'$ are both Delone-deriving and consist of bounded components. Set $\Sigma =C^{\infty }_{\phi \circ \pi }({\mathbb {R}}^d,\mathbb {C}^m,0)$. Then $\mathcal {P}\overset{{\mathrm {MLD}}}{\leftrightarrow }\mathcal {Q}$ if and only if $\Sigma _{\mathcal {P}}=\Sigma _{\mathcal {Q}}$.

### Proof

By Theorem 4.40, for each $\mathcal {P}\in \Pi \cup \Pi '$, there is $f\in \Sigma$ such that $\mathcal {P}\overset{{\mathrm {MLD}}}{\leftrightarrow }f$. The claim follows from Lemma 3.2. $\square$

Theorems 3.6 and 5.5 show that, in many cases, in order to study abstract patterns, it suffices to study the space $\Sigma _{\mathcal {P}}$ of certain pattern-equivariant functions.

## Appendix A: A Proof of Theorem 4.40

### Zero Element and Its Uniqueness

Here we discuss “zero elements.” Often there is only one zero element (Lemma A.3), and this fact plays an important role later. (See Lemma A.20.)

#### Definition A.1

Let $\Pi$ be an abstract pattern space over a topological space *X*. An element $\mathcal {P}\in \Pi$ such that ${{\,\mathrm{supp}\,}}\mathcal {P}=\emptyset$ is called a *zero element* of $\Pi$. If there is only one zero element in $\Pi$, it is denoted by 0.

#### Remark A.2

Zero elements always exist. In fact, take an arbitrary element $\mathcal {P}\in \Pi$. Then by Lemma 2.5, ${{\,\mathrm{supp}\,}}(\mathcal {P}\wedge \emptyset )=\emptyset$ and so $\mathcal {P}\wedge \emptyset$ is a zero element.

Zero elements need not be unique in general. For example, any set $\Pi$ is an abstract pattern space over any topological space *X* by defining a cutting-off operation via $\mathcal {P}\wedge C=\mathcal {P}$ for any $\mathcal {P}\in \Pi$ and $C\in {{\,\mathrm{Cl}\,}}(X)$. In this abstract pattern space, any element $\mathcal {P}\in \Pi$ is a zero element. The disjoint union of two abstract pattern spaces also has two zero elements.

#### Lemma A.3

If $\Pi$ is a glueable abstract pattern space over a topological space *X*, there is only one zero element in $\Pi$.

#### Proof

The subset $\emptyset$ of $\Pi$ is locally finite and pairwise compatible. Set $\mathcal {P}=\bigvee \emptyset$. By Lemma 4.5, $\mathcal {P}$ is a zero element. If $\mathcal {Q}$ is a zero element, then since $\mathcal {Q}$ is a majorant for $\emptyset$, we see $\mathcal {Q}\geqq \mathcal {P}$. We have $\mathcal {Q}=\mathcal {Q}\wedge \emptyset =\mathcal {P}$.$\square$

We finish this subsection by proving that zero elements play no role when we take supremum.

#### Lemma A.4

Let $\Pi$ be a glueable abstract pattern space over a topological space *X*. Take a locally finite and pairwise-compatible subset $\Xi$ of $\Pi$. Then $\bigvee (\Xi \cup \{0\})$ exists and $\bigvee (\Xi \cup \{0\})=\bigvee \Xi$.

#### Proof

For any $\mathcal {P}\in \Pi$, the abstract pattern $\mathcal {P}\wedge \emptyset$ is a zero element and, by the uniqueness of zero element (Lemma A.3), $\mathcal {P}\wedge \emptyset =0$ and $\mathcal {P}\geqq 0$. The existence and the value of the supremum will not be changed by adding the minimum element 0. $\square$

### A Supplementary Lemma for Local Derivability

#### Lemma A.5

Let $\Pi _1$ be an abstract pattern space over $(X,\Gamma )$ and $\Pi _2$ an abstract pattern space over $(Y,\Gamma )$. For two abstract patterns $\mathcal {P}_1\in \Pi _1$ and $\mathcal {P}_2\in \Pi _2$, consider the following three conditions:

1. There exist $x_{0}\in X$, $y_0\in Y$, and $R_0\geqq 0$ such that, if $\gamma ,\eta \in \Gamma$, $R\geqq 0$, and
  $$\begin{aligned} (\gamma \mathcal {P}_1)\wedge B(x_0,R+R_0)=(\eta \mathcal {P}_1)\wedge B(x_0,R+R_0), \end{aligned}$$
  then
  $$\begin{aligned} (\gamma \mathcal {P}_2)\wedge B(y_0,R)=(\eta \mathcal {P}_2)\wedge B(y_0,R). \end{aligned}$$
2. For any $x_{1}\in X$ and $y_1\in Y$ there exists $R_1\geqq 0$ such that, if $\gamma ,\eta \in \Gamma$, $R\geqq 0$, and
  $$\begin{aligned} (\gamma \mathcal {P}_1)\wedge B(x_1,R+R_1)=(\eta \mathcal {P}_1)\wedge B(x_1,R+R_1), \end{aligned}$$
  then
  $$\begin{aligned} (\gamma \mathcal {P}_2)\wedge B(y_1,R)=(\eta \mathcal {P}_2)\wedge B(y_1,R). \end{aligned}$$
3. $$\mathcal {P}_1\overset{{\mathrm {LD}}}{\rightarrow }\mathcal {P}_2$$.

Then, condition 1. and 2. are always equivalent, and condition 1. and 2. always imply condition 3. If the action $\Gamma \curvearrowright Y$ is transitive, $\Pi _2$ is glueable, $X=Y$, and $\mathcal {P}_2$ consists of bounded components (Definition 2.24), then condition 3. implies condition 1. and 2.

#### Proof

In order to prove the equivalence of 1. and 2. it suffices to prove the implication 1.$\Rightarrow$2. If we assume 1. there are $x_0$, $y_0$, and $R_0$ that satisfy the condition in 1. Take $x_1\in X$ and $y_1\in Y$ arbitrarily. Set $R_1=R_0+\rho _X(x_0,x_1)+\rho _Y(y_0,y_1)$, where $\rho _X,\rho _Y$ are the metrics for *X* and *Y*, respectively. Take $\gamma ,\eta \in \Gamma$ and $R>0$ arbitrarily and suppose

11 $$\begin{aligned} (\gamma \mathcal {P}_1)\wedge B(x_1,R_1+R)=(\eta \mathcal {P}_1)\wedge B(x_1,R_1+R). \end{aligned}$$

Since $B(x_0,R+\rho _Y(y_0,y_1)+R_0)\subset B(x_1, R_1+R)$, by taking cutting-off operation for both sides of (11), we obtain

$$\begin{aligned} (\gamma \mathcal {P}_1)\wedge B(x_0, R+\rho _{Y}(y_0,y_1)+R_0)= (\eta \mathcal {P}_1)\wedge B(x_0, R+\rho _{Y}(y_0,y_1)+R_0), \end{aligned}$$

and so

$$\begin{aligned} (\gamma \mathcal {P}_2)\wedge B(y_0, R+\rho _{Y}(y_0,y_1))= (\eta \mathcal {P}_2)\wedge B(y_0, R+\rho _{Y}(y_0,y_1)). \end{aligned}$$

By $$B(y_1,R)\subset B(y_0,R+\rho _Y(y_0,y_1))$$,

$$\begin{aligned} (\gamma \mathcal {P}_2)\wedge B(y_1, R)= (\eta \mathcal {P}_2)\wedge B(y_1, R). \end{aligned}$$

Next, we show that conditions 1. and 2. imply condition 3. If we assume condition (1), there exists $R_0$ as in the condition for some $x_0\in X$ and $y_0\in Y$. Take a compact $K_2\subset Y$. We can take $R>0$ such that $K_2\subset B(y_0,R)$. Set $K_1=B(x_0,R+R_0)$, then the definition of local derivability is satisfied.

Finally, we assume that $\mathcal {P}_2$ consists of bounded components, $\Pi _2$ is glueable, $X=Y$, the action $\Gamma \curvearrowright X$ is transitive, and condition 3. holds. We claim that condition 1. holds. Since $\mathcal {P}_2$ consists of bounded components, we can take $R_{\mathcal {P}_2}>0$ as in Definition 2.24. To prove condition 1., take $x_0\in X$. Set $K=B(x_0,2R_{\mathcal {P}_2})$. We can take a compact $K'\subset X$ as in the definition of local derivability. By compactness, there exists $R_0>0$ such that $K'\subset B(x_0,R_0)$. To prove that this $R_0$ has the desired property, take arbitrary $\gamma ,\eta \in \Gamma$ and $R\geqq 0$, and assume

$$\begin{aligned} (\gamma \mathcal {P}_1)\wedge B(x_0,R+R_0)=(\eta \mathcal {P}_1)\wedge B(x_0,R+R_0). \end{aligned}$$

Since $B(x_0,R)$ is compact, we can take its finite subset *F* such that $B(x_0,R)\subset \bigcup _{y\in F}B(y,R_{\mathcal {P}_2})$. By transitivity, for each $y\in F$, we can take $\gamma _y\in \Gamma$ such that $\gamma _yx_0=y$. Moreover, we have $\rho (\gamma ^{-1}_yx_0,x_0)=\rho (y,x_0)\leqq R$, and so

$$\begin{aligned} (\gamma ^{-1}_y\gamma \mathcal {P}_1)\wedge B(x_0,R_0)=(\gamma ^{-1}_y\eta \mathcal {P}_1) \wedge B(x_0,R_0), \end{aligned}$$

which implies that

$$\begin{aligned} (\gamma _y^{-1}\gamma \mathcal {P}_2)\wedge B(x_0,2R_{\mathcal {P}_2}) =(\gamma ^{-1}_y\eta \mathcal {P}_2)\wedge B(x_0,2R_{\mathcal {P}_2}) \end{aligned}$$

and

$$\begin{aligned} (\gamma \mathcal {P}_2)\wedge B(y,2R_{\mathcal {P}_2})=(\eta \mathcal {P}_2)\wedge B(y,2R_{\mathcal {P}_2}). \end{aligned}$$

Now, the set $\{(\gamma \mathcal {P}_2)\wedge B(x_0,R)\wedge B(y,2R_{\mathcal {P}_2})\,{|}\, y\in F\}$ is locally finite and pairwise compatible, and since $\Pi _2$ is glueable, this set admits a supremum $\mathcal {Q}$ and $\mathcal {Q}\leqq (\gamma \mathcal {P}_2)\wedge B(x_0,R)$. By the definition of $R_{\mathcal {P}_2}$, ${{\,\mathrm{supp}\,}}\mathcal {Q}={{\,\mathrm{supp}\,}}((\gamma \mathcal {P}_2)\wedge B(x_0,R))$ and so $(\gamma \mathcal {P}_2)\wedge B(x_0,R)=\mathcal {Q}$. The same argument holds for $\eta$. Hence

$$\begin{aligned} (\gamma \mathcal {P}_2)\wedge B(x_0,R)&= \bigvee \big \{(\gamma \mathcal {P}_2)\wedge B(x_0,R)\wedge B(y,2R_{\mathcal {P}_2})\,{|}\, y\in F\big \}\\&= \bigvee \big \{(\eta \mathcal {P}_2)\wedge B(x_0,R)\wedge B(y,2R_{\mathcal {P}_2})\,{|}\, y\in F\big \}\\&=(\eta \mathcal {P}_2)\wedge B(x_0,R), \end{aligned}$$

which completes the proof. $\square$

### Proofs for Claims in Sect. 4.2

#### Proof of Lemma 4.13

Set $\mathcal {P}=\bigvee \bigcup _{\lambda }\Xi _{\lambda }$. For each $\lambda \in \Lambda$ and $\mathcal {Q}\in \Xi _{\lambda }$, we have $\mathcal {P}\geqq \mathcal {Q}$ and so $\mathcal {P}\geqq \mathcal {Q}_{\lambda }$. This, in particular, shows that $\{\mathcal {Q}_{\lambda }\,{|}\,\lambda \}$ is pairwise compatible. Moreover, since for each $x\in X$ and $r>0$,

12 $$\begin{aligned} \{\mathcal {Q}_{\lambda }\wedge B(x,r)\,{|}\,\lambda \in \Lambda \}= \Bigl \{\bigvee (\Xi _{\lambda }\wedge B(x,r))\mid \lambda \in \Lambda \Bigr \}, \end{aligned}$$

$\Xi _{\lambda }\wedge B(x,r)\subset \bigl (\bigcup _{\lambda }\Xi _{\lambda }\bigr )\wedge B(x,r)$ and $\bigl (\bigcup \Xi _{\lambda }\bigr )\wedge B(x,r)$ is finite by assumption, the set (12) is finite: the set $\{\mathcal {Q}_{\lambda }\,{|}\,\lambda \}$ is locally finite.

If $\mathcal {P}'$ is a majorant for $\{\mathcal {Q}_{\lambda }\,{|}\,\lambda \}$, then $\mathcal {P}'\geqq \mathcal {Q}$ for each $\lambda \in \Lambda$ and $\mathcal {Q}\in \Xi _{\lambda }$, and so $\mathcal {P}'\geqq \mathcal {P}$. As mentioned above, $\mathcal {P}$ is a majorant for $\{\mathcal {Q}_{\lambda }\,{|}\,\lambda \}$, and so it is its supremum. $\square$

#### Proof of Lemma 4.15

If $\mathcal {P}\in \Xi$ and $\mathcal {Q}\in \Xi$, then there is $\mathcal {R}\in \Pi$ such that $\mathcal {R}\geqq \mathcal {P}$ and $\mathcal {R}\geqq \mathcal {Q}$. By Lemma 2.26, we see $\gamma \mathcal {R}\geqq \gamma \mathcal {P}$ and $\gamma \mathcal {R}\geqq \gamma \mathcal {Q}$, and so $\gamma \mathcal {P}$ and $\gamma \mathcal {Q}$ are compatible. If $x\in X$ and $r>0$ then, since $\gamma$ is an isometry, $\gamma ^{-1}B(x,r)=B(\gamma ^{-1}x,r)$. By

$$\begin{aligned} \big \{\gamma \mathcal {P}\wedge B(x,r)\,{|}\, \mathcal {P}\in \Xi \big \}= \gamma \big \{\mathcal {P}\wedge B(\gamma ^{-1}x,r)\,{|}\, \mathcal {P}\in \Xi \big \}, \end{aligned}$$

we see that this set is finite. We have proved that $\gamma \Xi$ is both pairwise compatible and locally finite.

Next, we show the latter statement. We use Lemma 2.26 several times. For any $\mathcal {P}\in \Xi$, $\gamma \bigvee \Xi \geqq \gamma \mathcal {P}$. This means that $\gamma \bigvee \Xi$ is a majorant for $\gamma \Xi$. To show this is the supremum, take a majorant $\mathcal {R}$ for $\gamma \Xi$. Then $\gamma ^{-1}\mathcal {R}$ is a majorant for $\Xi$ and so $\gamma ^{-1}\mathcal {R}\geqq \bigvee \Xi$. We have $\mathcal {R}\geqq \gamma \bigvee \Xi$, and so $\gamma \bigvee \Xi$ is the supremum for $\gamma \Xi$. $\square$

In the rest of this subsection, we show that ${{\,\mathrm{Map}\,}}(X,Y,y_0)$ (Example 2.16) is glueable, where *X* is a metric space, *Y* a set, and $y_0\in Y$. This is proved after proving preliminary technical lemmas.

#### Lemma A.6

Two maps $f,g\in {{\,\mathrm{Map}\,}}(X,Y,y_0)$ are compatible if and only if

$$\begin{aligned} f|_{{{\,\mathrm{supp}\,}}f\cap {{\,\mathrm{supp}\,}}g}=g|_{{{\,\mathrm{supp}\,}}f\cap {{\,\mathrm{supp}\,}}g}. \end{aligned}$$

#### Proof

Suppose *f* and *g* are compatible. Take a majorant $h\in {{\,\mathrm{Map}\,}}(X,Y,y_0)$. For each $x\in {{\,\mathrm{supp}\,}}f\cap {{\,\mathrm{supp}\,}}g$,

$$\begin{aligned} f(x)=(h\wedge {{\,\mathrm{supp}\,}}f)(x)=h(x)=(h\wedge {{\,\mathrm{supp}\,}}g)(x)=g(x). \end{aligned}$$

Conversely, suppose $f|_{{{\,\mathrm{supp}\,}}f\cap {{\,\mathrm{supp}\,}}g}=g|_{{{\,\mathrm{supp}\,}}f\cap {{\,\mathrm{supp}\,}}g}$. Define a map $h\in {{\,\mathrm{Map}\,}}(X,Y,y_0)$ by

$$\begin{aligned} h(x)= {\left\{ \begin{array}{ll} f(x)&{}\text {if }x\in {{\,\mathrm{supp}\,}}f,\\ g(x)&{}\text {if }x\in {{\,\mathrm{supp}\,}}g,\\ y_0 &{}\text {otherwise}. \end{array}\right. } \end{aligned}$$

This is well defined. Next, $h\geqq f$ because

$$\begin{aligned} (h\wedge {{\,\mathrm{supp}\,}}f)(x)=&{\left\{ \begin{array}{ll} h(x)&{}\text {if }x\in {{\,\mathrm{supp}\,}}f,\\ y_0&{}\text {if }x\notin {{\,\mathrm{supp}\,}}f \end{array}\right. }\\ =&{\left\{ \begin{array}{ll} f(x)&{}\text {if }x\in {{\,\mathrm{supp}\,}}f,\\ y_0&{}\text {if }x\notin {{\,\mathrm{supp}\,}}f \end{array}\right. }\\ =&f(x) \end{aligned}$$

for any $x\in X$. Similarly $h\geqq g$, and so *f* and *g* are compatible.$\square$

#### Lemma A.7

For $f\in {{\,\mathrm{Map}\,}}(X,Y,y_0)$, $x\in X$, and two positive numbers $r>s>0$, we have ${{\,\mathrm{supp}\,}}(f\wedge B(x,r))\supset ({{\,\mathrm{supp}\,}}f)\cap B(x,s)$. Consequently, $({{\,\mathrm{supp}\,}}(f\wedge B(x,r)))\cap B(x,s)=({{\,\mathrm{supp}\,}}f)\cap B(x,s)$.

#### Proof

Take $x'\in ({{\,\mathrm{supp}\,}}f)\cap B(x,s)$. For any $\varepsilon >0$, there is $x''\in B(x',\varepsilon )$ such that $f(x'')\ne y_0$. If $\varepsilon$ is small enough, this $x''$ is in *B*(*x*, *r*) and so $(f\wedge B(x,r))(x'')\ne y_0$. Since $\varepsilon$ was arbitrary, $x'\in {{\,\mathrm{supp}\,}}(f\wedge B(x,r))$. $\square$

#### Lemma A.8

Let $\Xi$ be a subset of ${{\,\mathrm{Map}\,}}(X,Y,y_0)$. Take $x\in X$ and two numbers *r*, *s* such that $r>s>0$. If $\Xi \wedge B(x,r)$ is finite, then $\{({{\,\mathrm{supp}\,}}f)\cap B(x,s)\,{|}\, f\in \Xi \}$ is finite.

#### Proof

Clear by Lemma A.7. $\square$

#### Lemma A.9

If $\Xi \subset {{\,\mathrm{Map}\,}}(X,Y,y_0)$ is locally finite, then $\bigcup _{f\in \Xi }{{\,\mathrm{supp}\,}}f$ is closed.

#### Proof

Take $x\in X\setminus \bigl (\bigcup _{f\in \Xi }{{\,\mathrm{supp}\,}}f\bigr )$. Since $\Xi \wedge B(x,1)$ is finite, by Lemma A.8, $\bigl \{({{\,\mathrm{supp}\,}}f)\cap B\bigl (x,\frac{1}{2}\bigr )\,{|}\, f\in \Xi \bigr \}$ is finite. There is $r>0$ such that $B(x,r)\cap {{\,\mathrm{supp}\,}}f=\emptyset$ for any $f\in \Xi$. $\square$

#### Proof of Proposition 4.19

Suppose $\Xi \subset {{\,\mathrm{Map}\,}}(X,Y,y_0)$ is locally finite and pairwise compatible. Set

$$\begin{aligned} f_{\Xi }(x)&= {\left\{ \begin{array}{ll} f(x)&{}\text {if there is }f\in \Xi \text { such that }x\in {{\,\mathrm{supp}\,}}f,\\ y_0&{}\text {otherwise} \end{array}\right. }\\&= {\left\{ \begin{array}{ll} f(x)&{}\text {if there is }f\in \Xi \text { such that }f(x)\ne y_0,\\ y_0&{}\text {otherwise}. \end{array}\right. } \end{aligned}$$

This is well defined by Lemma A.6. For each $f\in \Xi$ and $x\in X$,

$$\begin{aligned} (f_{\Xi }\wedge {{\,\mathrm{supp}\,}}f)(x)=&{\left\{ \begin{array}{ll} f_{\Xi }(x)&{}\text {if }x\in {{\,\mathrm{supp}\,}}f,\\ y_0&{}\text {if }x\notin {{\,\mathrm{supp}\,}}f \end{array}\right. }\\ =&{\left\{ \begin{array}{ll} f(x)&{}\text {if }x\in {{\,\mathrm{supp}\,}}f,\\ y_0&{}\text {if }x\notin {{\,\mathrm{supp}\,}}f \end{array}\right. }\\ =&f(x), \end{aligned}$$

and so $f_{\Xi }\geqq f$. In other words, $f_{\Xi }$ is a majorant for $\Xi$.

Next, we prove that $f_{\Xi }$ is the supremum for $\Xi$. To this end, we first claim ${{\,\mathrm{supp}\,}}f_{\Xi }=\bigcup _{f\in \Xi }{{\,\mathrm{supp}\,}}f$. It is clear that $\{x\in X\,{|}\, f_{\Xi }(x)\ne y_0\}\subset \bigcup _{f\in \Xi }{{\,\mathrm{supp}\,}}f$ because, if $f_{\Xi }(x)\ne y_0$, then there is $f\in \Xi$ such that $f(x)\ne y_0$. Together with Lemma A.9, we see ${{\,\mathrm{supp}\,}}f_{\Xi }\subset \bigcup _{f\in \Xi }{{\,\mathrm{supp}\,}}f$. Since $f_{\Xi }$ is a majorant for $\Xi$, the reverse inclusion is clear, and so ${{\,\mathrm{supp}\,}}f_{\Xi }=\bigcup _{f\in \Xi }{{\,\mathrm{supp}\,}}f$.

To prove that $f_{\Xi }$ is the supremum, we next take an arbitrary majorant *g* for $\Xi$. Since for $f\in \Xi$ and $x\in {{\,\mathrm{supp}\,}}f$, we have $g(x)=(g\wedge {{\,\mathrm{supp}\,}}f)(x)=f(x)$, we obtain

$$\begin{aligned} g\wedge \Big (\bigcup _{f\in \Xi }{{\,\mathrm{supp}\,}}f\Big )(x)=&{\left\{ \begin{array}{ll} g(x)&{}\text {if there is }f\in \Xi \text { such that }x\in {{\,\mathrm{supp}\,}}f,\\ y_0&{}\text {otherwise} \end{array}\right. }\\ =&{\left\{ \begin{array}{ll} f(x)&{}\text {if there is }f\in \Xi \text { such that }x\in {{\,\mathrm{supp}\,}}f,\\ y_0&{}\text {otherwise} \end{array}\right. }\\ =&f_{\Xi }(x) \end{aligned}$$

for each $x\in X$, and so $g\wedge ({{\,\mathrm{supp}\,}}f_{\Xi })=f_{\Xi }$, namely $g\geqq f_{\Xi }$. We have shown that $f_{\Xi }$ is the supremum for $\Xi$; $f_{\Xi }=\bigvee \Xi$.

It remains to show that $f_{\Xi }\wedge C$ is equal to $\bigvee (\Xi \wedge C)$ for each $C\in {{\,\mathrm{Cl}\,}}(X)$. This is the case because

$$\begin{aligned} (f_{\Xi }\wedge C)=&{\left\{ \begin{array}{ll} f_{\Xi }(x)&{}\text {if }x\in C,\\ y_0 &{}\text {otherwise} \end{array}\right. }\\ =&{\left\{ \begin{array}{ll} f(x)&{}\text {if }x\in C\text { and }f(x)\ne y_0\text { for some }f\in \Xi ,\\ y_0&{}\text {otherwise} \end{array}\right. }\\ =&{\left\{ \begin{array}{ll} (f\wedge C)(x)&{}\text {if there is }f\in \Xi \text { such that } (f\wedge C)(x)\ne y_0,\\ y_0&{}\text {otherwise}. \end{array}\right. } \end{aligned}$$

$$\square $$

### A Proof for Proposition 4.28 in Sect. 4.3

#### Proof for Proposition 4.28

$\underline{\hbox {Step 1: We prove }\mathcal {P}\overset{{\mathrm {LD}}}{\rightarrow }(P_{\lambda })_{\lambda }.}$ Let $R_1>0$ be a constant for the local derivation $\mathcal {P}\overset{{\mathrm {LD}}}{\rightarrow }D$ for points 0 and 0 which appears in Lemma A.5, condition 2. Let $L_0$ be an arbitrary positive real number. Set $L_1=L_0+R_0+R_1$. We assume $\gamma ,\eta \in \Gamma$ and

13 $$\begin{aligned} (\gamma \mathcal {P})\wedge B(0,L_1)=(\eta \mathcal {P})\wedge B(0,L_1) \end{aligned}$$

and show

14 $$\begin{aligned} (\gamma P_{\lambda })\cap B(e,L_0)=(\eta P_{\lambda })\cap B(e,L_0) \end{aligned}$$

for each $$\lambda \in \Lambda $$.

Take $\lambda \in \Lambda$ and fix it. By (13), we see

$$\begin{aligned} (\gamma D)\cap B(0,L_0+R_0)=(\eta D)\cap B(0,L_0+R_0). \end{aligned}$$

Let $\zeta$ be an element of $P_{\lambda }$ such that $\gamma \zeta \in B(e,L_0)$. We claim that $\gamma \zeta \in \eta P_{\lambda }$. By the definition of the plan, $\zeta 0\in D$ and $\zeta \mathcal {P}_{\lambda }=\mathcal {P}\wedge B(\zeta 0,R_0)$. Since $\rho (\gamma \zeta 0,0)\leqq \rho _{\Gamma }(\gamma \zeta ,e)\leqq L_0$, $\gamma \zeta 0\in (\gamma D)\cap B(0,L_0)=(\eta D)\cap B(0,L_0)$, and so there is $y\in D$ such that $\eta y=\gamma \zeta 0$. Now

$$\begin{aligned} \gamma \zeta \mathcal {P}_{\lambda }&=(\gamma \mathcal {P})\wedge B(\gamma \zeta 0,R_0)\\&=(\gamma \mathcal {P})\wedge B(0,L_1)\wedge B(\gamma \zeta 0,R_0)\\&=(\eta \mathcal {P})\wedge B(0,L_1)\wedge B(\eta y,R_0)\\&=\eta (\mathcal {P}\wedge B(y,R_0)). \end{aligned}$$

We have proved $\eta ^{-1}\gamma \zeta 0\in D$ and $\eta ^{-1}\gamma \zeta \mathcal {P}_{\lambda }=\mathcal {P}\wedge B(\eta ^{-1}\gamma \zeta 0,R_0)$, and so $\eta ^{-1}\gamma \zeta \in P_{\lambda }$, by which we proved the claim. Thus $(\gamma P_{\lambda })\cap B(e,L_0)\subset (\eta P_{\lambda })\cap B(e,L_0)$, and by symmetry we have shown (14).

$\underline{\hbox {Step 2: We prove }(P_{\lambda })_{\lambda }\overset{{\mathrm {LD}}}{\rightarrow }\mathcal {P}.}$ Let $L_0>0$ be an arbitrary positive number and set $L_1=L_0+R_0+2$. Assume $\gamma ,\eta \in \Gamma$ and

15 $$\begin{aligned} (\gamma P_{\lambda })\cap B(e,L_1)=(\eta P_{\lambda })\cap B(e,L_1) \end{aligned}$$

holds for each $\lambda \in \Lambda$. For each $\lambda \in \Lambda$ and $\xi \in P_{\lambda }$, if we have $(\gamma \xi \mathcal {P}_{\lambda })\wedge B(0,L_0)\ne 0$, then $B(\gamma \xi 0,R_0)\cap B(0,L_0)\ne \emptyset$. This implies $\rho (\gamma \xi 0,0)\leqq L_0+R_0$ and $\rho _{\Gamma }(\gamma \xi ,e)\leqq L_0+R_0+2=L_1$. We have the same observation if we replace $\gamma$ with $\eta$. Thus

$$\begin{aligned}&\bigl \{(\gamma \xi \mathcal {P}_{\lambda })\wedge B(0,L_0) \mid \lambda \in \Lambda , \xi \in P_{\lambda }\bigr \}\cup \{0\}\\&\quad =\bigl \{(\gamma \xi \mathcal {P}_{\lambda })\wedge B(0,L_0))\mid \lambda \in \Lambda , \xi \in P_{\lambda }\text { and }\gamma \xi \in B(e,L_1)\bigr \}\cup \{0\}\\&\quad =\bigl \{(\eta \zeta \mathcal {P}_{\lambda })\wedge B(0,L_0)\mid \lambda \in \Lambda , \zeta \in P_{\lambda }\text { and }\eta \zeta \in B(e,L_1)\bigr \}\cup \{0\}\\&\quad =\bigl \{(\eta \zeta \mathcal {P}_{\lambda })\wedge B(0,L_0)\mid \lambda \in \Lambda ,\zeta \in P_{\lambda }\bigr \} \cup \{0\}. \end{aligned}$$

We obtain the desired result by Lemmas A.4 and 4.15:

$$\begin{aligned} (\gamma \mathcal {P})\wedge B(0,L_0)&=\bigvee \bigl \{(\gamma \xi \mathcal {P}_{\lambda })\wedge B(0,L_0) \mid \lambda \in \Lambda , \xi \in P_{\lambda }\bigr \}\cup \{0\}\\&=\bigvee \bigl \{(\eta \zeta \mathcal {P}_{\lambda })\wedge B(0,L_0) \mid \lambda \in \Lambda , \zeta \in P_{\lambda }\bigr \}\cup \{0\}\\&=(\eta \mathcal {P})\wedge B(0,L_0).\square \end{aligned}$$

### Preliminary Lemmas

Here we prove some technical lemmas which are used in the next subsection.

#### Lemma A.10

Let $(X,\rho )$ be a metric space and $\Pi$ be an abstract pattern space over *X*. Let $F_j$ be a finite subset of *X* for $j=1,2$. Take a positive real number *r* such that, for each $j=1,2$, any two distinct elements $x,y\in F_j$ satisfy $\rho (x,y)>4r$. Suppose that, for each *j* and $x\in F_j$, there corresponds $\mathcal {P}_{x}^j\in \Pi$ such that $\emptyset \ne {{\,\mathrm{supp}\,}}\mathcal {P}_{x}^j\subset B(x,r)$. Suppose also that there is $Q^j=\bigvee \{\mathcal {P}_{x}^j\,{|}\, x\in F_j\}$ for $j=1,2$. Then the following statements hold:

1. If ${{\,\mathrm{supp}\,}}\mathcal {Q}^1\subset {{\,\mathrm{supp}\,}}\mathcal {Q}^2$, then for each $x\in F_1$ there is a unique $y\in F_2$ such that ${{\,\mathrm{supp}\,}}\mathcal {P}_{x}^1\cap {{\,\mathrm{supp}\,}}\mathcal {P}_{y}^2\ne \emptyset$. In this case ${{\,\mathrm{supp}\,}}\mathcal {P}_{x}^1\subset {{\,\mathrm{supp}\,}}\mathcal {P}_{y}^2$ holds.
2. If ${{\,\mathrm{supp}\,}}\mathcal {Q}^1={{\,\mathrm{supp}\,}}\mathcal {Q}^2$, then for each $x\in F_1$ there is a unique $y\in F_2$ such that ${{\,\mathrm{supp}\,}}\mathcal {P}_{x}^1={{\,\mathrm{supp}\,}}\mathcal {P}_{y}^2$.
3. If $\mathcal {Q}^1=\mathcal {Q}^2$, then for each $x\in F_1$ there is a unique $y\in F_2$ such that $\mathcal {P}_{x}^1=\mathcal {P}_{y}^2$.

#### Proof

(1) By Lemma 4.5, ${{\,\mathrm{supp}\,}}\mathcal {Q}^j=\bigcup _{x\in F_j}{{\,\mathrm{supp}\,}}\mathcal {P}^{j}_{x}$ for each $j=1,2$. For each $x\in F_1$, there is $y\in F_2$ such that ${{\,\mathrm{supp}\,}}\mathcal {P}_{x}^1\cap {{\,\mathrm{supp}\,}}\mathcal {P}_y^2\ne \emptyset$. If there is another $y'\in F_2$ such that ${{\,\mathrm{supp}\,}}\mathcal {P}_x^1\cap {{\,\mathrm{supp}\,}}\mathcal {P}_{y'}^2\ne \emptyset$, then $B(x,r)\cap B(y,r)\ne \emptyset$ and $B(x,r)\cap B(y',r)\ne \emptyset$ and so $\rho (y,y')\leqq 4r$. By definition of *r*, we have $y=y'$. This shows the uniqueness of *y*. Uniqueness implies the last statement.

(2) By (1), for each $x\in F_1$ there is $y\in F_2$ such that ${{\,\mathrm{supp}\,}}\mathcal {P}_x^1\subset {{\,\mathrm{supp}\,}}\mathcal {P}_y^2$. Applying (1) again, there is $x'\in F_1$ such that ${{\,\mathrm{supp}\,}}\mathcal {P}_y^2\subset {{\,\mathrm{supp}\,}}\mathcal {P}_{x'}^1$. We have ${{\,\mathrm{supp}\,}}\mathcal {P}_x^1\subset {{\,\mathrm{supp}\,}}\mathcal {P}_{x'}^1$ and, by applying uniqueness in (1), we see $x=x'$.

(3) By (2), for each $x\in F_1$ there is $y\in F_2$ such that ${{\,\mathrm{supp}\,}}\mathcal {P}_x^1={{\,\mathrm{supp}\,}}\mathcal {P}_y^2$. Then

$$\begin{aligned} \mathcal {P}_x^1=\mathcal {Q}^1\wedge {{\,\mathrm{supp}\,}}\mathcal {P}_1^x= \mathcal {Q}^2\wedge {{\,\mathrm{supp}\,}}\mathcal {P}_2^y =\mathcal {P}_y^2. \end{aligned}$$

Uniqueness follows from uniqueness in (1). $\square$

By using this we can prove the following lemma:

#### Lemma A.11

Let $\Pi$ be an abstract pattern space over $({\mathbb {R}}^d,\Gamma )$, where ${\mathbb {R}}^d\subset \Gamma \subset {\mathbb {R}}^d\rtimes \mathrm{O}(d)$. Let $\Sigma$ be a subshift of $\Pi$. Take $r>0$ arbitrarily. Let $\mathfrak {F}$ be a family of building blocks for *r*. Take two admissible digits $(\mathcal {D}_{\mathcal {P}}^1)_{\mathcal {P}\in \mathfrak {F}}$ and $(\mathcal {D}_{\mathcal {P}}^2)_{\mathcal {P}\in \mathfrak {F}}$. Suppose both $\bigcup _{\mathcal {P}}\mathcal {D}_{\mathcal {P}}^1$ and $\bigcup _{\mathcal {P}}\mathcal {D}_{\mathcal {P}}^2$ are finite. Suppose also that

$$\begin{aligned} \bigvee \big \{\gamma \mathcal {P}\,{|}\, \mathcal {P}\in \mathfrak {F}, \gamma \in \mathcal {D}_{\mathcal {P}}^1\big \} = \bigvee \big \{\gamma \mathcal {P}\,{|}\, \mathcal {P}\in \mathfrak {F},\gamma \in \mathcal {D}_{\mathcal {P}}^2\big \}. \end{aligned}$$

Then, for any $\mathcal {P}\in \mathfrak {F}$ and $\gamma \in \mathcal {D}_{\mathcal {P}}^1$, there is $\eta \in \mathcal {D}_{\mathcal {P}}^2$ such that $\gamma \mathcal {P}=\eta \mathcal {P}$.

#### Proof

Consider two finite sets

$$\begin{aligned} F_1= \biggl \{\gamma 0 \ {\big |} \ \gamma \in \bigcup _{\mathcal {P}}\mathcal {D}_{\mathcal {P}}^1\biggr \} \end{aligned}$$

and

$$\begin{aligned} F_2 = \biggl \{\gamma 0 \ {\big |} \ \gamma \in \bigcup _{\mathcal {P}}\mathcal {D}_{\mathcal {P}}^2\biggr \}. \end{aligned}$$

For each $x\in F_1$, there are $\mathcal {P}\in \mathfrak {F}$ and $\gamma \in \Gamma _{\mathcal {P}}^1$ such that $x=\gamma 0$. Set $\mathcal {P}_x^1=\gamma \mathcal {P}$. This is independent of the choice of $\mathcal {P}$ and $\gamma$. Define $\mathcal {P}_x^2$ for each $x\in F_2$ in a similar way. The claim follows from Lemma A.10.$\square$

The following lemma will be useful when we construct the family of building blocks $\{\mathcal {E},\mathcal {P}_0\}$ and $\{\mathcal {R}_{\lambda }\,{|}\, \lambda \in \Lambda \}$ in the next subsection.

#### Lemma A.12

Let $\Pi$ be an abstract pattern space over $({\mathbb {R}}^d,\Gamma )$, where ${\mathbb {R}}^d\subset \Gamma \subset {\mathbb {R}}^d\rtimes \mathrm{O}(d)$. Let $\Sigma$ be a subshift of $\Pi$. Let $\mathfrak {F}$ be a family of building blocks for *r*. Take a real number $r'>2r$ arbitrarily. Let $(\mathcal {D}_{\mathcal {P}}^{\lambda })_{\mathcal {P}\in \mathfrak {F}}$ be an admissible digit for each $\lambda$, where $\lambda$ belongs to an index set $\Lambda$, such that

1. for each $\lambda \in \Lambda$, we have $\bigcup _{\mathcal {P}\in \mathfrak {F}}\mathcal {D}_{\mathcal {P}}^{\lambda }\ne \emptyset$, and
2. for each $\lambda$ and $\mathcal {P}$, any element $\gamma \in \mathcal {D}_{\mathcal {P}}^{\lambda }$ satisfies a condition
  16 $$\begin{aligned} \rho (0,\gamma 0)<r'-2r. \end{aligned}$$

Set $\mathcal {Q}_{\lambda }=\bigvee \{\gamma \mathcal {P}\,{|}\, \mathcal {P}\in \mathfrak {F}, \gamma \in \mathcal {D}_{\mathcal {P}}^{\lambda }\}$ for each $\lambda \in \Lambda$. Then the family $\{\mathcal {Q}_{\lambda }\,{|}\, \lambda \in \Lambda \}$ satisfies the first two conditions of the definition of family of building blocks for $r'$ (Definition 4.33).

#### Proof

*The first condition*: Take $\lambda \in \Lambda$ and fix it. Since ${{\,\mathrm{supp}\,}}\mathcal {Q}_{\lambda }= \overline{ \bigcup _{\mathcal {P}\in \mathfrak {F},\gamma \in \mathcal {D}_{\mathcal {P}}^{\lambda }}{{\,\mathrm{supp}\,}}\gamma \mathcal {P}}$, it is nonempty. We have moreover ${{\,\mathrm{supp}\,}}\gamma \mathcal {P}\subset B(\gamma 0,r)\subset B(0,r')$ by (16), for each $\mathcal {P}\in \mathfrak {F}$ and $\gamma \in \mathcal {D}_{\mathcal {P}}^{\lambda }$, and so ${{\,\mathrm{supp}\,}}\mathcal {Q}_{\lambda }\subset B(0,r')$.

*The second condition*: Take $\lambda ,\mu \in \Lambda$ and $\gamma ,\eta \in \Gamma$ such that $\rho (\gamma 0,\eta 0)>4r'$. We show that $\gamma \mathcal {Q}_{\lambda }$ and $\eta \mathcal {Q}_{\mu }$ are compatible. For each $\mathcal {P},\mathcal {Q}\in \mathfrak {F}$, $\xi \in \mathcal {D}_{\mathcal {P}}^{\lambda }$, and $\zeta \in \mathcal {D}_{\mathcal {Q}}^{\mu }$, by (16), we have $\rho (\gamma \xi 0,\eta \zeta 0)>4r$. Thus $\gamma \xi \mathcal {P}$ and $\eta \zeta \mathcal {Q}$ are compatible and so together with Lemma 4.37, the set $\Xi _1\cup \Xi _2$ is locally finite and pairwise compatible. Here,

$$\begin{aligned} \Xi _1=\big \{\gamma \xi \mathcal {P}\,{|}\, \mathcal {P}\in \mathfrak {F},\xi \in \Gamma _{\mathcal {P}}^{\lambda }\big \} \end{aligned}$$

and

$$\begin{aligned} \Xi _2=\big \{\eta \zeta \mathcal {Q}\,{|}\, \mathcal {Q}\in \mathfrak {F},\zeta \in \Gamma _{\mathcal {Q}}^{\mu }\big \}. \end{aligned}$$

By Lemma 4.13 and the facts that $\gamma \mathcal {Q}_{\lambda }=\bigvee \Xi _1$ and $\eta \mathcal {Q}_{\mu }=\bigvee \Xi _2$, we see that $\gamma \mathcal {Q}_{\lambda }$ and $\eta \mathcal {Q}_{\mu }$ are compatible. $\square$

#### Remark A.13

In Lemma A.12, the third condition of the definition of family of building blocks (Definition 4.33) is not always satisfied. When we use this lemma in Sect. A.6, we prove the third condition in an ad hoc way.

The following lemmas (Lemmas A.14 and A.18) will be used when we construct $\mathcal {E}$ in the next subsection.

#### Lemma A.14

Let $\mathfrak {G}$ be a set of subgroups of $\Gamma _0$ which is at most countable. Suppose $\max _{G\in \mathfrak {G}}{{\,\mathrm{card}\,}}G<\infty$. Then, for each two numbers *r*, *s* such that $r>s>0$, there are $\varepsilon >0$ and a point $y_G\in B(0,r)^{\circ }\setminus B(0,s)$ for each $G\in \mathfrak {G}$ such that

1. if $G\in \mathfrak {G}$ and $\gamma \in G\setminus \{e\}$, then $\rho (y_G,\gamma y_G)>\varepsilon$, and
2. if $$G\ne H$$, then $$\rho (0,y_G)\ne \rho (0,y_H)$$.

To prove Lemma A.14, we prepare the following notation:

#### Notation A.15

For any $A\in \mathrm{O}(d)$, $r>0$, and $\varepsilon \geqq 0$, set

$$\begin{aligned} S_{A,\varepsilon ,r}=\big \{x\in B(0,r)\,{|}\, \rho (Ax,x)\leqq \varepsilon \big \}. \end{aligned}$$

We prove two lemmas beforehand in order to prove Lemma A.14.

#### Lemma A.16

If the order of an element $A\in \mathrm{O}(d)$ is less than an integer *m*, then $S_{A,\varepsilon ,r}\subset S_{A,0,r}+B\bigl (0,\frac{m}{2}\varepsilon \bigr )$.

#### Proof

Take an element $x\in S_{A,\varepsilon ,r}$. Let *k* be the order of *A*. Set $y=\frac{1}{k}\sum _{j=0}^{k-1}A^j x$. By convexity of *B*(0, *r*), *y* is in *B*(0, *r*), and so $y\in S_{A,0,r}$. Moreover,

$$\begin{aligned} \rho (x,y)&=\biggl \Vert \frac{1}{k}\sum (A^jx-x)\biggr \Vert \\&\leqq \frac{1}{k}\sum _{j=0}^{k-1}\sum _{i=0}^{j-1}\Vert A^ix-A^{i+1}x\Vert \\&\leqq \frac{1}{k}\sum _{j=0}^{k-1}j\varepsilon \leqq \frac{m}{2}\,\varepsilon .\square \end{aligned}$$

#### Lemma A.17

Let *m* be a positive integer and *r* be a positive real number. We have $\lim _{\varepsilon \rightarrow 0}\mu (S_{A,\varepsilon ,r})=0$ uniformly for all $A\in \mathrm{O}(d)\setminus \{e\}$ such that the order of *A* is less than *m*.

#### Proof

For each such *A* there is a $d-1$-dimensional vector subspace $V_A$ of ${\mathbb {R}}^d$ such that $S_{A,0,r}\subset V_A\cap B(0,r)$, and so $S_{A,\varepsilon ,r}\subset (V_A\cap B(0,r))+B\bigl (0,\frac{m}{2}\varepsilon \bigr )$. For any $d-1$-dimensional vector subspace *V* of ${\mathbb {R}}^d$, the limit $\lim _{\varepsilon \rightarrow 0}\mu \bigl ((V\cap B(0,r))+B\bigl (0,\frac{m}{2}\varepsilon \bigr )\bigr )$ converges uniformly to 0.$\square$

#### Proof of Lemma A.14

If $\varepsilon$ is small enough, for any $A\in \mathrm{O}(d)\setminus \{e\}$ of order at most *m*, $m\mu (S_{A,\varepsilon ,r})<\mu (B(0,r)^{\circ }\setminus B(0,s))$. This implies that $B(0,r)^{\circ }\setminus B(0,s)$ is not included in $\bigcup _{A\in G,A\ne e}S_{A,\varepsilon ,r}$ for any $G\in \mathfrak {G}$. To take each $y_G$, we enumerate $\mathfrak {G}$ as $\mathfrak {G}=\{G_1,G_2,\ldots \}$. First take $y_{G_1}\in B(0,r)^{\circ }\setminus \bigl (B(0,s)\cup \bigcup _{A\in G_1,A\ne e}S_{A,\varepsilon ,r}\bigr )$. If we have taken $y_{G_1}, y_{G_2},\ldots , y_{G_{n-1}}$, we can take $y_{G_n}\in B(0,r)^{\circ }\setminus \bigl (B(0,s)\cup \bigcup _{A\in G_n,A\ne e}S_{A,\varepsilon ,r}\bigr )$ such that $\Vert y_{G_n}\Vert \ne \Vert y_{G_j}\Vert$ for each $j=1,2,\ldots , n-1$. In this way, we can take $y_{G_1},y_{G_2},\ldots$ with the desired condition.$\square$

We finish this subsection by proving two lemmas, which we will use to prove Theorem 4.40. For each $j=1,2,\ldots , d$, let $e_j\in {\mathbb {R}}^d$ be the vector whose *i*th component is 0 for $i\ne j$ and whose *j*th component is 1.

#### Lemma A.18

For any $r>0$ there is a subset $F\subset B(0,r)$ such that

- $$1<{{\,\mathrm{card}\,}}F<\infty $$, and
- $${{\,\mathrm{Sym}\,}}_{\Gamma }F=\{e\}$$.

#### Proof

Take for each $j=1,2,\ldots , d$ a positive number $r_j>0$. Set $F=\{0,r_1e_1,r_2e_2,\ldots ,r_de_d\}$. If any two $r_j$’s are different but all close to 1, then 0 is the only vector in *F* such that the distances from any other vectors are close to 1. Thus if $\gamma \in \Gamma$ and $\gamma F=F$, then $\gamma 0=0$. Since the $r_j$’s are all different, $\gamma r_je_j=r_je_j$ for each *j*, and since $\{r_1e_1,\ldots ,r_de_d\}$ is a basis for ${\mathbb {R}}^d$, $\gamma$ must be *e*.$\square$

#### Lemma A.19

For any $r>0$ and $R>0$ there are $R'>0$ and $C_1>0$ such that, if $x\in {\mathbb {R}}^d$ and *D* is a Delone set of ${\mathbb {R}}^d$ which is *R*-relatively dense and *r*-uniformly discrete, then

17 $$\begin{aligned} {{\,\mathrm{card}\,}}({{\,\mathrm{Sym}\,}}_{\Gamma _x}D\cap B(x,R'))<C_1. \end{aligned}$$

#### Proof

Take $R'>0$ large enough so that, if $e'_1,e'_2,\ldots , e'_d\in {\mathbb {R}}^d$ and $\Vert e_j-e'_j\Vert <\frac{R}{R'-R}$ for each *j*, then $\{e'_1,e'_2,\ldots , e'_d\}$ is linear independent. Set $C_1>k!$, where *k* is an integer such that $k>\frac{\mu (B(0,R'+r))}{\mu (B(0,\frac{r}{2}))}$.

Take (*R*, *r*)-Delone set *D* and $x\in {\mathbb {R}}^d$ arbitrarily. For each $j=1,2,\ldots , d$, there is $x_j\in D\cap B(x+(R'-R)e_j,R)$. Then, for each *j*, we have $\Vert \frac{1}{R'-R}(x_j-x)-e_j\Vert <\frac{R}{R'-R}$ and so the set of vectors $\{x_j-x\,{|}\, j=1,2,\ldots , d\}$ is a basis for ${\mathbb {R}}^d$.

If $\gamma \in \Gamma _x$ and $\gamma (y)=y$ for each $y\in D\cap B(x,R')$, then since $\gamma$ fixes $x,x_1,x_2,\ldots , x_d$, $\gamma =e$. Thus we have an embedding of ${{\,\mathrm{Sym}\,}}_{\Gamma _x}D\cap B(x,R')$ into the permutation group of the set $D\cap B(x,R')$. Since, for any two distinct $y,z\in D\cap B(x,R')$, we have $B(y,r/2)\cap B(z,r/2)=\emptyset$, we see that $\mu (B(0,r/2)){{\,\mathrm{card}\,}}D\cap B(x,R')\leqq \mu (B(0,R'+r))$. The order of the permutation group is less than $C_1$ which we took above. We thus see the inequality (17). $\square$

### Proof of Translation Theorem

We now start the proof of Theorem 4.40. Let $\mathcal {P}\in \Pi _1$ be an abstract pattern that consists of bounded components (Definition 2.24). Suppose $\mathcal {P}$ is Delone-deriving; that is, there is a Delone set *D* in ${\mathbb {R}}^d$ such that $\mathcal {P}\overset{{\mathrm {LD}}}{\rightarrow }D$. We will use Proposition 4.28, so we first prove the following:

#### Lemma A.20

There exists $R_0>0$ such that $(D,R_0)$ decomposes $\mathcal {P}$ (Definition 4.20).

#### Proof

The set *D* is Delone so that it is $R_D$-relatively dense for a positive $R_D>0$ and $r_D$-uniformly discrete for some $r_D>0$. For these $R_D$ and $r_D$, there are $R'$ and $C_1$ as in Lemma A.19. The abstract pattern $\mathcal {P}$ consists of bounded components so that there is $R_{\mathcal {P}}$ as in Definition 2.24. Since *D* is locally derivable from $\mathcal {P}$, there is a constant $R_{LD}>0$ as in condition 2. in Lemma A.5 for $x_1=y_1=0$. Take $R_0>R_D+R_{\mathcal {P}}+R_{LD}+R'$.

The first condition of Definition 4.20 is satisfied by the assumption.

*The Second Condition of Definition* 4.20. First, we show that $\{\mathcal {P}\wedge B(x,R_0)\,{|}\, x\in D\}$ is locally finite and pairwise compatible. For each $x\in {\mathbb {R}}^d$ and $r>0$, we have an inclusion

$$\begin{aligned} \big \{y\in D\,{|}\, B(y,R_0)\cap B(x,r)\ne \emptyset \big \}\subset D\cap B(x,R_0+r) \end{aligned}$$

and the latter is finite. Hence $\{\mathcal {P}\wedge B(y,R_0)\wedge B(x,r)\,{|}\, y\in D\}$ is finite, since it is a zero element except for finitely many *y*’s, and by Lemma A.3, zero element is unique. On the other hand, pairwise compatibility is clear since, for each $\mathcal {P}\wedge B(x,R_0)$, $\mathcal {P}$ is a majorant.

Since $\Pi _1$ is glueable, there is the supremum $\mathcal {Q}=\bigvee \{\mathcal {P}\wedge B(x,R_0)\,{|}\, x\in D\}$. On the one hand, we see by Lemma 4.5 that ${{\,\mathrm{supp}\,}}\mathcal {Q}=\overline{\bigcup _{x\in D}{{\,\mathrm{supp}\,}}(\mathcal {P}\wedge B(x,R_0))} \subset {{\,\mathrm{supp}\,}}\mathcal {P}$; on the other hand, if $y\in {{\,\mathrm{supp}\,}}\mathcal {P}$, then

$$\begin{aligned} y\in {{\,\mathrm{supp}\,}}(\mathcal {P}\wedge B(y,R_{\mathcal {P}}))\subset {{\,\mathrm{supp}\,}}(\mathcal {P}\wedge B(x,R_0)) \subset {{\,\mathrm{supp}\,}}\mathcal {Q}\end{aligned}$$

for some $x\in D$, and so ${{\,\mathrm{supp}\,}}\mathcal {P}\subset {{\,\mathrm{supp}\,}}\mathcal {Q}$. Therefore ${{\,\mathrm{supp}\,}}\mathcal {P}={{\,\mathrm{supp}\,}}\mathcal {Q}$. Since $\mathcal {P}\geqq \mathcal {P}\wedge B(x,R_0)$ for each $x\in D$ and $\mathcal {Q}$ is the supremum of such abstract patterns, we have $\mathcal {P}\geqq \mathcal {Q}$. Thus $\mathcal {P}=\mathcal {P}\wedge {{\,\mathrm{supp}\,}}\mathcal {P}=\mathcal {P}\wedge {{\,\mathrm{supp}\,}}\mathcal {Q}=\mathcal {Q}$ by the definition of order $\geqq$ (Definition 4.1).

*The Third Condition of Definition* 4.20. For each $x\in D$, take $\gamma \in {{\,\mathrm{Sym}\,}}_{\Gamma _x}\mathcal {P}\wedge B(x,R_0)$. Then

$$\begin{aligned} (\gamma \mathcal {P})\wedge B(x,R_0)=\gamma (\mathcal {P}\wedge B(x,R_0))=\mathcal {P}\wedge B(x,R_0), \end{aligned}$$

and since $\mathcal {P}\overset{{\mathrm {LD}}}{\rightarrow }D$ with respect to the constant $R_{LD}$, we have

$$\begin{aligned} \gamma (D\cap B(x,R'))=(\gamma D)\cap B(x,R')=D\cap B(x,R'). \end{aligned}$$

This means that $\gamma \in {{\,\mathrm{Sym}\,}}_{\Gamma _x}D\cap B(x,R')$. By definition of $C_1$, ${{\,\mathrm{card}\,}}{{\,\mathrm{Sym}\,}}_{\Gamma _x}\mathcal {P}\wedge B(x,R_0)\leqq {{\,\mathrm{card}\,}}{{\,\mathrm{Sym}\,}}_{\Gamma _x}D\cap B(x,R')<C_1$. $\square$

By Lemma A.20, there is $R_0>0$ such that $(D,R_0)$ decomposes $\mathcal {P}$, and so we can find a tuple of components and plan, as follows.

By Lemma 4.22, there is a set $\Lambda$ and a tuple of components $(\mathcal {P}_{\lambda })_{\lambda \in \Lambda }$. Let $(C_{\lambda })_{\lambda \in \Lambda }$ be the plan for $\mathcal {P}$ with respect to $(D,R_0,(\mathcal {P}_{\lambda })_{\lambda })$. Then we have the following:

- $\Lambda$ is a set which is at most countable.
- Since each $\mathcal {P}_{\lambda }$ is a copy of an abstract pattern of the form $\mathcal {P}\wedge B(x,R_0)$ ($x\in D$) by an element $\gamma \in \Gamma$ such that $\gamma x=0$, by Definition 4.20 we have the following: $G_{\lambda }={{\,\mathrm{Sym}\,}}_{\Gamma _{0}}\mathcal {P}_{\lambda }$ is a finite group, for each $\lambda \in \Lambda$, and $\max _{\lambda }{{\,\mathrm{card}\,}}G_{\lambda }<\infty$.
- For each $\lambda \in \Lambda$, $C_{\lambda }$ is a subset of $\Gamma$ such that
  18 $$\begin{aligned} C_{\lambda }G_{\lambda }= C_{\lambda }. \end{aligned}$$
- *D* is a Delone set such that
  19 $$\begin{aligned} D=\big \{\gamma 0\,{|}\, \lambda \in \Lambda , \gamma \in C_{\lambda }\big \}. \end{aligned}$$
- There is $r_0>0$ such that
  20 $$\begin{aligned}&\text { if }\lambda ,\mu \in \Lambda , \gamma \in C_{\lambda }, \eta \in C_{\mu }\text { and }\rho (\gamma 0,\eta 0)\leqq 4r_0,\text { then } \gamma 0=\eta 0,\nonumber \\&\text {and so }\lambda =\mu \text { and }\gamma ^{-1}\eta \in G_{\lambda }. \end{aligned}$$

By Proposition 4.28, we have $\mathcal {P}\overset{{\mathrm {MLD}}}{\leftrightarrow }(C_{\lambda })_{\lambda }$. To prove $\mathcal {S}\overset{{\mathrm {MLD}}}{\leftrightarrow }\mathcal {P}$ for some $\mathcal {S}\in \Sigma$, we construct an abstract pattern $\mathcal {S}$ in $\Sigma$ such that $\mathcal {S}\overset{{\mathrm {MLD}}}{\leftrightarrow }(C_{\lambda })_{\lambda }$. It consists of three steps. ($\mathcal {E}$ and $\mathcal {R}_{\lambda }$ are shown in Figs. 2 and 3.)

$\underline{\hbox {Step 1: construction of }\mathcal {E}.}$ As described in the beginning of Sect. 4, we will construct a family of building blocks $(\mathcal {R}_{\lambda })_{\lambda }$. In order to construct this, we first construct a building block $\mathcal {E}$, from which each $\mathcal {R}_{\lambda }$ is constructed.

By Lemma A.14, there are $y_{\lambda }\in B\bigl (0,\frac{3}{4}r_0\bigr )\setminus B\bigl (0,\frac{1}{2}r_0\bigr )$ for each $\lambda \in \Lambda$ and $r_1\in \bigl (0,\frac{1}{8}r_0\bigr )$ such that

- $$\inf \{\rho (\gamma y_{\lambda },y_{\lambda })\,{|}\, \lambda \in \Lambda , \gamma \in G_{\lambda }\setminus \{e\}\}>4r_1>0$$, and
- if $\lambda ,\mu$ are two distinct elements of $\Lambda$, then we have $\rho (0,y_{\lambda })\ne \rho (0,y_{\mu })$.

By Lemma A.18, there are $F\subset B\bigl (0,\frac{1}{2}r_1\bigr )$ and $r_2\in \bigl (0,\frac{1}{4}r_1\bigr )$ such that

- If $x,y\in F$ and $x\ne y$, then $\rho (x,y)>4r_2$,
- $${{\,\mathrm{Sym}\,}}_{\Gamma }F=\{e\}$$, and
- $$\infty>{{\,\mathrm{card}\,}}F>1$$.

Take $\gamma _x\in \Gamma$, for each $x\in {\mathbb {R}}^d$, such that $\gamma _x0=x$.

#### Notation A.21

Let $\mathcal {P}_0$ be a symmetric building block of $\Sigma$ for $r_2$. (Its existence is assumed.) Set $\mathcal {E}=\bigvee \{\gamma _x\mathcal {P}_0\,{|}\, x\in F\}$.

#### Remark A.22

Since points of *F* are separated by the distance $4r_2$, by the definition of building block the set $\{\gamma _x\mathcal {P}_0\,{|}\, x\in F\}$ is pairwise compatible. Since it is a finite set, it is locally finite. Its supremum exists.

If $\Sigma$ is the set of all *r*-uniformly discrete sets in ${\mathbb {R}}^d$, this $\mathcal {E}$ is nothing but *F* itself, which has trivial symmetry. In general cases, we can also prove that $\mathcal {E}$ has trivial symmetry:

#### Lemma A.23

$${{\,\mathrm{Sym}\,}}_{\Gamma }\mathcal {E}=\{e\}$$.

#### Proof

Take $\gamma \in \Gamma$ such that $\gamma \mathcal {E}=\mathcal {E}$. Since $\gamma \mathcal {E}=\bigvee \{\gamma \gamma _x\mathcal {P}_0\,{|}\, x\in F\}$, by Lemma A.11, for each $x\in F$, there is $y\in F$ such that $\gamma \gamma _x\mathcal {P}_0=\gamma _y\mathcal {P}_0$. By the definition of building block (Definition 4.33), we have $\gamma \gamma _x0=\gamma _y0$ and $\gamma x=y$. This implies that $\gamma F\subset F$ and $\gamma F=F$, which implies that $\gamma =e$. $\square$

We use $\{\mathcal {P}_0,\mathcal {E}\}$ to construct $\mathcal {R}_{\lambda }$’s. To this aim, we need to show the following:

#### Lemma A.24

The set $\{\mathcal {P}_0,\mathcal {E}\}$ is a family of building blocks of $\Sigma$ for $r_1$.

#### Proof

We apply Lemma A.12. The sets $\{e\}$ and $\{\gamma _x\,{|}\, x\in F\}$ play the role of admissible digits. If $x\in F$, then

$$\begin{aligned} \rho (\gamma _x 0,0)=\rho (x,0)\leqq \frac{1}{2}~r_1<r_1-2r_2, \end{aligned}$$

and so by Lemma A.12 the first two axioms for family of building blocks are satisfied.

Since $\mathcal {P}_0$ is a building block, we have ${{\,\mathrm{Sym}\,}}_{\Gamma }\mathcal {P}_0\subset \Gamma _{0}$. Moreover, ${{\,\mathrm{Sym}\,}}_{\Gamma }\mathcal {E}=\{e\}\subset \Gamma _{0}$. Finally, we never have $\gamma \mathcal {P}_0=\mathcal {E}$ for any $\gamma \in \Gamma$. If this holds, we have, by Lemma A.11, $\gamma _x\mathcal {P}_0=\gamma \mathcal {P}_0$ for any $x\in F$, and this implies $x=\gamma _x0=\gamma 0$ for each $x\in F$. This contradicts the fact that ${{\,\mathrm{card}\,}}F>1$. $\square$

$\underline{\hbox {Step 2: construction of }\mathcal {R}_{\lambda }.}$ For each $\lambda \in \Lambda$, set

$$\begin{aligned} \mathcal {R}_{\lambda }=\bigvee \big \{\mathcal {P}_0\}\cup \{\gamma \gamma _{y_{\lambda }}\mathcal {E}\,{|}\, \gamma \in G_{\lambda }\big \}. \end{aligned}$$

We use $\mathcal {R}_{\lambda }$’s to construct $\mathcal {S}$. To this aim, we need to show the following:

#### Lemma A.25

The set $\{\mathcal {R}_{\lambda }\,{|}\, \lambda \in \Lambda \}$ is a family of building blocks for $r_0$.

#### Proof

Since $\gamma 0=0$, we have, for each $\gamma \in G_{\lambda }$,

$$\begin{aligned} \rho (\gamma \gamma _{y_{\lambda }}0,0)=\rho (0,y_{\lambda })>\frac{1}{2}~r_0>4r_1, \end{aligned}$$

and by definition of $y_{\lambda }$’s, for each distinct $\gamma ,\eta \in G_{\lambda }$,

$$\begin{aligned} \rho (\gamma \gamma _{y_{\lambda }}0,\eta \gamma _{y_{\lambda }}0)= \rho (\eta ^{-1}\gamma y_{\lambda },y_{\lambda })>4r_1 \end{aligned}$$

we see that the pair of $\{e\}$ and $\{\gamma \gamma _{y_{\lambda }}\,{|}\, \gamma \in G_{\lambda }\}$ forms an admissible digit for each $\lambda \in \Lambda$. Moreover, by

$$\begin{aligned} \rho (\gamma \gamma _{y_{\lambda }}0,0)=\rho (0,y_{\lambda }) \leqq \frac{3}{4}~r_0<r_0-2r_1, \end{aligned}$$

we see, by Lemma A.12, that the first two axioms for the building blocks are satisfied.

Suppose $\lambda ,\mu \in \Lambda$, $\gamma _0\in \Gamma$, and $\gamma _0\mathcal {R}_{\lambda }=\mathcal {R}_{\mu }$. By Lemma A.11, we have $\gamma _0\mathcal {P}_0=\mathcal {P}_0$ and so $\gamma _0 0=0$. Again by Lemma A.11, there is $\gamma \in G_{\mu }$ such that $\gamma _0\gamma _{y_{\lambda }}\mathcal {E}=\gamma \gamma _{y_{\mu }}\mathcal {E}$, and so by Lemma A.23, $\gamma _0\gamma _{y_{\lambda }}=\gamma \gamma _{y_{\mu }}$. This implies that (since $\gamma _0$ and $\gamma$ fix 0)

$$\begin{aligned} \rho (0,y_{\lambda })=\rho (0,\gamma _0\gamma _{y_{\lambda }}0) =\rho (0,\gamma \gamma _{y_{\mu }}0) =\rho (0,y_{\mu }) \end{aligned}$$

and so $$\lambda =\mu $$. $$\square $$

We need the fact that $\mathcal {R}_{\lambda }$ has the same symmetry group as $\mathcal {P}_{\lambda }$:

#### Lemma A.26

$${{\,\mathrm{Sym}\,}}_{\Gamma }\mathcal {R}_{\lambda }=G_{\lambda }$$ for each $$\lambda $$.

#### Proof

Take $\gamma _0\in {{\,\mathrm{Sym}\,}}_{\Gamma }\mathcal {R}_{\lambda }$. By Lemma A.11, there is $\gamma \in G_{\lambda }$ such that $\gamma _0\gamma _{y_{\lambda }}\mathcal {E}=\gamma \gamma _{y_{\lambda }}\mathcal {E}$ and so by Lemma A.23 we have $\gamma _0=\gamma \in G_{\lambda }$.

On the other hand, if $\gamma _0\in G_{\lambda }$, then

$$\begin{aligned} \gamma _0\mathcal {R}_{\lambda }=&\bigvee \big \{\gamma _0\mathcal {P}_0\}\cup \{\gamma _0\gamma \gamma _{y_{\lambda }}\mathcal {E}\,{|}\, \gamma \in G_{\lambda }\big \}\\ =&\bigvee \big \{\mathcal {P}_0\}\cup \{\gamma \gamma _{y_{\lambda }}\mathcal {E}\,{|}\, \gamma \in G_{\lambda }\big \}\\ =&\mathcal {R}_{\lambda }, \end{aligned}$$

since $\mathcal {P}_0$ is a symmetric building block.$\square$

$$\underline{\hbox {Step 3: Construction of }\mathcal {S}\hbox { and its property}.}$$

Here we construct $\mathcal {S}\in \Sigma$ by using $\mathcal {R}_{\lambda }$’s in exactly the same way as $\mathcal {P}$ is constructed from $\mathcal {P}_{\lambda }$’s, that is, with respect to the plan $(C_{\lambda })_{\lambda }$. We show that $\mathcal {S}$ and $(C_{\lambda })_{\lambda }$ are MLD (Theorem A.31) by using Proposition 4.28, and this shows that $\mathcal {P}$ and $\mathcal {S}$ are MLD since $\mathcal {P}$ and $(C_{\lambda })_{\lambda }$ are also MLD by again using Proposition 4.28.

Define

$$\begin{aligned} \mathcal {S}=\bigvee \big \{\gamma \mathcal {R}_{\lambda }\,{|}\, \lambda \in \Lambda , \gamma \in C_{\lambda }\big \}. \end{aligned}$$

By (20), $(C_{\lambda })_{\lambda }$ is an admissible digit for $(\mathcal {R}_{\lambda })_{\lambda \in \Lambda }$ and so $\mathcal {S}$ is well defined. We first prove that $(D,r_0)$ decomposes $\mathcal {S}$ (Lemma A.29), then prove that $(C_{\lambda })_{\lambda \in \Lambda }$ is the plan (Lemma A.30). By these we can use Proposition 4.28.

In the following two lemmas we check the conditions in Definition 4.20.

#### Lemma A.27

$$\mathcal {S}\overset{{\mathrm {LD}}}{\rightarrow }D$$.

#### Proof

Let *R* be an arbitrary positive real number. Set $L=R+3r_0$. Assume $\gamma ,\eta \in \Gamma$ and

21 $$\begin{aligned} (\gamma \mathcal {S})\wedge B(0,L)=(\eta \mathcal {S})\wedge B(0,L). \end{aligned}$$

Set $\Xi =\{\xi \mathcal {R}_{\lambda }\,{|}\, \lambda \in \Lambda , \xi \in C_{\lambda }\}$, then by (21) and Lemma 4.15, we see

$$\begin{aligned} \bigvee (\gamma \Xi \wedge B(0,L))=\bigvee (\eta \Xi \wedge B(0,L)). \end{aligned}$$

Consider the following two finite sets:

$$\begin{aligned} F_1=\big \{\gamma \xi 0\,{|}\, \lambda \in \Lambda ,\xi \in C_{\lambda }, \gamma \xi \mathcal {R}_{\lambda }\wedge B(0,L)\ne 0\big \} \end{aligned}$$

and

$$\begin{aligned} F_2=\big \{\eta \zeta 0\,{|}\, \lambda \in \Lambda ,\zeta \in C_{\lambda }, \eta \zeta \mathcal {R}_{\lambda }\wedge B(0,L)\ne 0\big \}. \end{aligned}$$

For each $x=\gamma \xi 0\in F_1$, we consider an abstract pattern $\mathcal {P}_x^1=\gamma \xi \mathcal {R}_{\lambda }\wedge B(0,L)$. This is included in $B(\gamma \xi 0,r_0)$. For $F_2$ we define $\mathcal {P}_x^2$’s in a similar way. We can apply Lemma A.10 and obtain the following: if $\lambda \in \Lambda$, $\xi \in C_{\lambda }$, and $\gamma \xi \mathcal {R}_{\lambda } \wedge B(0,L)\ne 0$, there are $\mu \in \Lambda$ and $\zeta \in C_{\mu }$ such that

$$\begin{aligned} (\gamma \xi \mathcal {R}_{\lambda })\wedge B(0,L)=(\eta \zeta \mathcal {R}_{\mu })\wedge B(0,L). \end{aligned}$$

Now we prove $(\gamma D)\cap B(0,R)\subset (\eta D)\cap B(0,R)$. Take an element $\gamma \xi 0$ from the left-hand side set, where $\xi \in C_{\lambda }$ for some $\lambda$ and $\gamma \xi 0\in B(0,R)$. Then ${{\,\mathrm{supp}\,}}\gamma \xi R_{\lambda }\subset B(0,R+r_0)$. As in the previous paragraph, there are $\mu \in \Lambda$ and $\zeta \in C_{\mu }$ such that $\gamma \xi \mathcal {R}_{\lambda }=(\eta \zeta \mathcal {R}_{\mu })\wedge B(0,L)$. The support of this abstract pattern is included in $B(0,R+r_0)$, and the support of $\eta \zeta \mathcal {R}_{\mu }$ has a diameter less than $2r_0$; we have ${{\,\mathrm{supp}\,}}(\eta \zeta \mathcal {R}_{\mu })\subset B(0,L)$ and so $\gamma \xi \mathcal {R}_{\lambda }=\eta \zeta \mathcal {R}_{\mu }$. Since $(\mathcal {R}_{\lambda })_{\lambda }$ is a family of building blocks, we see that $\lambda =\mu$ and $\gamma \xi 0=\eta \zeta 0\in \eta D$. We have proved $(\gamma D)\cap B(0,R)\subset (\eta D)\cap B(0,R)$, and by symmetry the reverse inclusion is true. $\square$

#### Lemma A.28

For each $\lambda \in \Lambda$ and $\gamma \in C_{\lambda }$, we have

$$\begin{aligned} \mathcal {S}\wedge B(\gamma 0,r_0)=\gamma \mathcal {R}_{\lambda }. \end{aligned}$$

#### Proof

If $\mu \in \Lambda$, $\eta \in C_{\mu }$, and $\eta \mathcal {R}_{\mu }\wedge B(\gamma 0,r_0)\ne 0$, then $\rho (\gamma 0,\eta 0)\leqq 2r_0$ and so by (20), $\lambda =\mu$ and $\gamma \mathcal {R}_{\lambda }=\eta \mathcal {R}_{\mu }$. Hence

$$\begin{aligned} \mathcal {S}\wedge B(\gamma 0,r_0)&=\bigvee \big \{\eta \mathcal {R}_{\mu }\wedge B(\gamma 0,r_0)\,{|}\, \mu \in \Lambda , \eta \in C_{\mu }\big \}\\&=\bigvee \big \{\gamma \mathcal {R}_{\lambda }\wedge B(\gamma 0 ,r_0)\big \}\\&=\gamma \mathcal {R}_{\lambda }. \square \end{aligned}$$

#### Lemma A.29

The pair $(D,r_0)$ decomposes $\mathcal {S}$.

#### Proof

Clear by the definition of $\mathcal {S}$ and Lemmas A.28, A.27, and A.26.$\square$

#### Lemma A.30

$(\mathcal {R}_{\lambda })_{\lambda }$ is a tuple of components for $\mathcal {S}$ with respect to $(D,r_0)$, and $(C_{\lambda })$ is the plan for $\mathcal {S}$ with respect to $(D,r_0,(\mathcal {R}_{\lambda })_{\lambda \in \Lambda })$.

#### Proof

Take $x\in D$ arbitrarily. By (19), there are $\lambda \in \Lambda$ and $\gamma \in C_{\lambda }$ such that $x=\gamma 0$, and by Lemma A.28, $\mathcal {S}\wedge B(x,r_0)=\mathcal {S}\wedge B(\gamma 0,r_0)=\gamma \mathcal {R}_{\lambda }$. The uniqueness of such $\lambda$ is clear since $(\mathcal {R}_{\mu })_{\mu \in \Lambda }$ is a family of building blocks. We have shown that $(\mathcal {R}_{\mu })$ is a tuple of components for $\mathcal {P}_0$ with respect to $(D,r_0)$.

Next we show that $(C_{\lambda })_{\lambda }$ is the plan. If $\mu \in \Lambda$ and $\gamma \in C_{\mu }$, then $\gamma 0\in D$ by (19) and $\mathcal {S}\wedge B(\gamma 0,r_0)=\gamma \mathcal {R}_{\mu }$ by Lemma A.28, and so $\gamma \in P_{\mu }(\mathcal {S},D,r_0,(\mathcal {R}_{\lambda }))$. Conversely, if $\gamma \in P_{\mu }(\mathcal {S},D,r_0,(\mathcal {R}_{\lambda }))$, then $\gamma 0\in D$ and $\gamma \mathcal {R}_{\mu }=\mathcal {S}\wedge B(\gamma 0,r_0)$. By (19), there are $\nu \in \Lambda$ and $\eta \in C_{\nu }$ such that $\gamma 0=\eta 0$, and so by Lemma A.28, $\eta \mathcal {R}_{\nu }=\mathcal {S}\wedge B(\eta 0,r_0)$. This implies that $\gamma \mathcal {R}_{\mu }=\eta \mathcal {R}_{\nu }$, and so $\mu =\nu$ and $\eta ^{-1}\gamma \in {{\,\mathrm{Sym}\,}}_{\Gamma }\mathcal {R}_{\mu }=G_{\mu }$. By (18), we see $\gamma =\eta \eta ^{-1}\gamma \in C_{\mu }G_{\mu }=C_{\mu }$. We have proved $C_{\mu }=P_{\mu }(\mathcal {S},D,r_0,(\mathcal {R}_{\lambda }))$ for any $\mu \in \Lambda$. $\square$

#### Theorem A.31

$$\mathcal {S}\overset{{\mathrm {MLD}}}{\leftrightarrow }(C_{\lambda })_{\lambda }$$.

#### Proof

Clear from Lemma A.30 and Proposition 4.28. $\square$

#### Corollary A.32

$$\mathcal {P}\overset{{\mathrm {MLD}}}{\leftrightarrow }\mathcal {S}$$.

#### Proof

By Proposition 4.28, $\mathcal {P}\overset{{\mathrm {MLD}}}{\leftrightarrow }(C_{\lambda })$ because $(C_{\lambda })$ is a plan for $\mathcal {P}$. Combined with Theorem A.31 we have $\mathcal {P}\overset{{\mathrm {MLD}}}{\leftrightarrow }\mathcal {S}$. $\square$

#### Lemma A.33

${{\,\mathrm{supp}\,}}\mathcal {S}$ is relatively dense.

#### Proof

For any $x\in {\mathbb {R}}^d$ there is $y\in D$ near *x*. By (19), there are $\lambda \in \Lambda$ and $\gamma \in C_{\lambda }$ such that $y=\gamma 0$. Since ${{\,\mathrm{supp}\,}}\gamma \mathcal {R}_{\lambda }\subset B(y,r_0)$, any point in ${{\,\mathrm{supp}\,}}\gamma \mathcal {R}_{\lambda }$, which is a point in ${{\,\mathrm{supp}\,}}\mathcal {S}$, is near *x*.$\square$

This lemma completes the proof of Theorem 4.40.

## Footnotes

1 Note that every second-countable locally compact group admits a left-invariant proper metric [13].

2 In [2] patterns are assumed to be nonempty, but it is useful to include the empty set as a pattern.
